# A Community-Based Culture Collection for Targeting Novel Plant Growth-Promoting Bacteria from the Sugarcane Microbiome

**DOI:** 10.3389/fpls.2017.02191

**Published:** 2018-01-04

**Authors:** Jaderson Silveira Leite Armanhi, Rafael Soares Correa de Souza, Natália de Brito Damasceno, Laura M. de Araújo, Juan Imperial, Paulo Arruda

**Affiliations:** ^1^Centro de Biologia Molecular e Engenharia Genética, Universidade Estadual de Campinas, Campinas, Brazil; ^2^Departamento de Genética e Evolução, Instituto de Biologia, Universidade Estadual de Campinas, Campinas, Brazil; ^3^Centro de Biotecnología y Genómica de Plantas, Universidad Politécnica de Madrid, Instituto Nacional de Investigación y Tecnología Agraria y Alimentaria, Madrid, Spain; ^4^Consejo Superior de Investigaciones Científicas, Madrid, Spain

**Keywords:** community-based culture collection (CBC), maize, microbiome, plant growth-promoting (PGP), sugarcane, synthetic community

## Abstract

The soil-plant ecosystem harbors an immense microbial diversity that challenges investigative approaches to study traits underlying plant-microbe association. Studies solely based on culture-dependent techniques have overlooked most microbial diversity. Here we describe the concomitant use of culture-dependent and -independent techniques to target plant-beneficial microbial groups from the sugarcane microbiome. The community-based culture collection (CBC) approach was used to access microbes from roots and stalks. The CBC recovered 399 unique bacteria representing 15.9% of the rhizosphere core microbiome and 61.6–65.3% of the endophytic core microbiomes of stalks. By cross-referencing the CBC (culture-dependent) with the sugarcane microbiome profile (culture-independent), we designed a synthetic community comprised of naturally occurring highly abundant bacterial groups from roots and stalks, most of which has been poorly explored so far. We then used maize as a model to probe the abundance-based synthetic inoculant. We show that when inoculated in maize plants, members of the synthetic community efficiently colonize plant organs, displace the natural microbiota and dominate at 53.9% of the rhizosphere microbial abundance. As a result, inoculated plants increased biomass by 3.4-fold as compared to uninoculated plants. The results demonstrate that abundance-based synthetic inoculants can be successfully applied to recover beneficial plant microbes from plant microbiota.

## Introduction

Microbial community profiling by culture-independent techniques has transformed our knowledge of the diversity and extent of plant-associated microbiomes (Bulgarelli et al., 2012, 2013, 2015; Lundberg et al., 2012; Edwards et al., 2015; Panke-Buisse et al., 2015; Zarraonaindia et al., 2015; Coleman-Derr et al., 2016; Castrillo et al., 2017). Culturing representative microbes of plant-associated microbiota and performing direct inoculation experiments are therefore crucial steps toward understanding the mechanisms involved in growth promotion, interspecies interactions and community assemblage (Zengler et al., 2002; Vartoukian et al., 2010; Bai et al., 2015; Lebeis et al., 2015; Castrillo et al., 2017; Finkel et al., 2017). Several studies have reported the beneficial contributions of key microorganisms or microbial communities in plant development by conducting inoculation assays under sterile conditions (Bai et al., 2015; Timm et al., 2016; Castrillo et al., 2017). However, the isolation of plant-associated microorganisms has been driven by the screening of known traits for plant growth promotion, using group-specific culture media, which may restrict discovery of novel mechanisms and neglect dominant microbial groups associated to plants. The development of new approaches to target microorganisms based on microbial ecology data, such as assemblage pattern and abundance, may help unravel novel mechanisms and traits associated with the plant-microbe interaction.

Root-colonizing bacteria that exert beneficial effects such as nitrogen fixation and phytohormone production have been identified in sugarcane in the past decades (Döbereiner, 1961; Döbereiner et al., 1972; Cavalcante and Döbereiner, 1988; Olivares et al., 1996; Bastián et al., 1998; Fuentes-Ramirez and Caballero-Mellado, 2005). However, the organ distribution and abundance of these bacterial groups compared with other microorganisms inhabiting the sugarcane plant remained unknown until recently, when the diversity, organ-specific assemblages, and abundance of bacterial and fungal communities during plant development have been investigated (de Souza et al., 2016). It was found that a core microbiome comprised of <~20% of the total microbial diversity represented over ~90% of the relative abundance of bacterial and fungal OTUs (operational taxonomic units) assembled in the different plant organs. Surprisingly, the commonly investigated microbial groups in association to sugarcane comprise a small fraction of the total diversity. On the other hand, highly abundant microbial groups inhabiting sugarcane are comprised by understudied microbes (de Souza et al., 2016).

Microorganisms have traditionally been isolated from plants as axenic cultures, using culture media specific to microbial groups (Brown et al., 2012; Bai et al., 2015; Castrillo et al., 2017). These methods require successive rounds of picking and streaking to obtain pure cultures. Although this approach has allowed the identification of plant-beneficial bacteria, the medium selectivity prevents the growth of most plant-associated microbes (Schloss and Handelsman, 2005; Lebeis et al., 2012; Stewart, 2012; Turner et al., 2013). Moreover, some microorganisms might not be amenable to isolation due to strict mutual dependencies among microbes (Schink, 2002; Barea et al., 2005). We have recently introduced the concept of community-based culture collection (CBC) (Armanhi et al., 2016) as an alternative method for large-scale isolation of microorganisms of plant-associated microbiota. The CBC approach is based on picking non-confluent colonies from primary platings regardless of whether they comprise single or multiple microorganisms, therefore, allowing culturing communities instead of solely pure colonies.

Here, we present a strategy to target and investigate plant growth-promoting (PGP) bacteria based on microbial community profile of plant organs. We use the CBC approach (Armanhi et al., 2016) to annotate our entire sugarcane culture collection and identify representatives of dominant microbial groups associated to sugarcane. A synthetic community comprising highly abundant bacteria from root and stalk core microbiomes was assembled as an inoculant. Then the abundance-based synthetic inoculant was probed using maize as a plant model. The results are discussed in the context of using relative abundance profile of plant microbiota to target beneficial microbes.

## Materials and methods

### The sugarcane community-based culture collection (CBC)

A CBC representative of the sugarcane microbiome was constructed by sampling the rhizosphere, endophytic root, and endophytic stalks of mature sugarcane (*Saccharum* sp.) variety SP 80-3280. Plants were harvested from the same site where samples were collected to profile sugarcane microbiome by culture-independent methods (de Souza et al., 2016). Rhizosphere community was obtained by washing roots in ice-cold 1 × PBS (137 mM NaCl, 2.7 mM KCl, 10 mM Na_2_HPO_4_, 2 mM KH_2_PO_4_, and pH 7.0) with 0.05% Tween 20 solution. Microorganisms from endophytic root and stalk were collected by homogenizing plant tissue in PBS. For each plant organ, an enriched microbial sample was obtained by centrifugation (Supplementary Figure S1). Enriched microbial samples were plated on half-strength Luria-Bertani (LB) medium, supplemented with sugarcane juice containing 8 or 35 g l^−1^ of total reducing sugars (TRS; Supplementary Table S1), and yeast-peptone-dextrose (YPD) medium. Microbial isolates were recovered by picking colonies from these primary platings regardless of whether they contain single or multiple microbes. Isolates were grown in liquid media, tested for growth viability (Supplementary Figure S2) and stored in 96-well plates (see “Construction of the sugarcane CBC” in Supplementary Methods S1).

### Dna extraction, 16S rRNA gene sequencing for microbe identification, and taxonomic classification

Microbial identification was performed by the 16S rRNA gene multiplex amplicon sequencing by PacBio (Armanhi et al., 2016) using 8f and 1492r primers to target the V1–V9 regions (Supplementary Table S2). Raw data were deposited at Sequence Read Archive (SRA) database under the accession number SRP126483. The assembly of raw sequences into CCSs (circular consensus sequences), CCS demultiplexing, quality filtering, and clustering into OTUs were carried out as previously described (Armanhi et al., 2016). The CCSs were firstly clustered into well-OTUs (wOTUs, i.e., OTUs obtained after CCS clustering within the wells). Then, wOTUs sequences were reclustered into collection-OTUs (cOTUs, i.e., OTUs obtained after wOTU clustering among the wells; Supplementary Figure S3A and “Sequencing and data processing” in Supplementary Methods S1). The taxonomic prediction was assigned for cOTUs using “utax” in USEARCH v8.1 (Edgar, 2010) and RDP (Cole et al., 2005) database (www.drive5.com/utax/rdp_16s.fa) with a confidence score of 0.9.

Phylogenetic analysis was performed by multiple alignments of cOTU sequences using Clustal Omega v1.2.1 (Sievers et al., 2011) with 1,000 iterations. A phylogenetic tree was constructed by maximum likelihood using QIIME v1.8.0 (Caporaso et al., 2010) and FastTree v2.1.3 (Price et al., 2009). The tree was visualized using GraPhlAn v0.9.7 (Asnicar et al., 2015).

### Cross-referencing of CBC (culture-dependent) data with the sugarcane microbiome (culture-independent) data

The CBC dataset was cross-referenced with the sugarcane microbiome dataset (de Souza et al., 2016) by sequence alignment (Supplementary Figures S3B–D). Initially, the sugarcane profile dataset was filtered by aligning the microbiome-OTUs (mOTUs, i.e., OTUs obtained by community assemblage analysis of the sugarcane microbiome) with the Greengenes database (DeSantis et al., 2006) using “usearch_global” in USEARCH v8.1. Sequences with <75% identity were discarded (Supplementary Figure S3B). The cross-referencing was performed by sequence alignment of the filtered mOTUs (culture-independent method) with the CCSs from the microbe identification dataset of the CBC (culture-dependent method; Supplementary Figure S3C). The link between mOTUs and cOTUs were made by mapping back the CCSs to its respective cOTUs (Supplementary Figures S3C,D and “Cross-referencing” in Supplementary Methods S1). Stalk regions were divided in bottom, medium and upper stalk by similar number of internodes as previously described (de Souza et al., 2016).

### Assemblage and inoculation of an abundance-based synthetic community

A total of 17 wells containing highly abundant bacteria from the sugarcane core microbiome was selected to construct a synthetic community. Bacteria from each well were individually grown in liquid culture media to late exponential phase, and equivalent optical density (OD) were mixed to reach the final OD_620 nm_ of 0.6. The culture mix was centrifuged and the pelleted was resuspended in 0.1 × Hoagland's solution (Hoagland and Arnon, 1950).

The abundance-based synthetic inoculum was probed using maize as a plant model. Seeds of the commercial maize (*Zea mays* L.) hybrid DKB 177 (DeKalb; Monsanto, Brazil) were surface-sterilized by firstly rinsing twice in a solution of 0.1% (v/v) Tween 20 (Sigma, Saint Louis, MO, USA) for 5 min under constant agitation. After washing seeds twice in sterile distilled deionized water to remove remaining Tween 20, seeds were soaked in 15% (v/v) commercial bleach solution for 10 min under constant agitation. Seeds were then seven times washed in distilled deionized water to eliminate remaining Tween 20 and bleach solutions. Surface-sterilized seeds were properly arranged in sterile filter papers rolls, maintained moist and sterile. Seeds were pre-germinated in the dark at 28°C for 3 d.

Seedlings were aseptically dissected from their endosperm and scutellum to limit the nutrient availability and force plants to acquire nutrients from the substrate. Endosperm-free seedlings were planted in bottom-holed pots of 25 cm height and 10 cm diameter filled with vermiculite. Plants were subjected to three inoculation events: germinated embryo axes were soaked in the inoculum for 30 min right before planting and 1 mL of inoculum was pipetted to each planted seedling, and two further direct applications to the plant base at 2 d and 1 week after planting. Plants were maintained well-watered with sterile distilled deionized water and irrigated every 3 d with 50 mL of modified Hoagland's nutrient solution (see “Maize growth conditions” in Supplementary Methods S1). Thirteen replicates were used for each treatment in a randomized experimental design. Eight 4-week-old plants per treatment were harvested and had their fresh and dry weight measured. To assess dry weight plants were dried at 65°C for 7 d.

### Microbiota profiling of inoculated and uninoculated plants

Five inoculated and five uninoculated 4-week-old plants were harvested, and their leaves, stems and roots microbial communities sampled by methods adapted from previously described protocol (de Souza et al., 2016). DNA was extracted from enriched microbial samples and used for library preparation for 16S rRNA gene sequencing as previously described (de Souza et al., 2016). Libraries were sequenced in the HiSeq 2500 Illumina sequencer. All raw data were deposited at SRA database under the accession number SRP116051. The bioinformatics pipeline used USEARCH v9.2 commands (Edgar, 2010) unless otherwise specified (Supplementary Figure S4). Sequences were filtered using maximum expected error 0.25 (Edgar and Flyvbjerg, 2015), and by size, from 230 to 270 nucleotides. Plastid sequences were removed using the DUK (Li et al., 2011). OTUs were obtained by sequence clustering at 97% identity using an UPARSE-based (Edgar, 2013) pipeline (see “Microbiota profiling” in Supplementary Methods S1).

### Analysis of the colonization pattern of the abundance-based synthetic community

The OTUs from inoculated and uninoculated plants were identified using SINTAX (Edgar, 2016) in USEARCH v9.2 and the SINTAX-compatible SILVA 16S rRNA gene database (www.drive5.com/sintax/silva_16s_v123.fa.gz). The Bray–Curtis dissimilarity matrix was calculated and employed for principal coordinates analysis (PCoA) and analysis of similarity (ANOSIM) using QIIME. The differential relative abundance of each OTU among samples was determined with a Kruskal–Wallis test with *P* < 0.05 (see “Colonization analysis” in Supplementary Methods S1).

## Results

### The sugarcane CBC targeted a significant portion of the sugarcane core microbiome

We used the multiplex amplicon sequencing method (Armanhi et al., 2016) to elucidate the bacterial composition of each well of our sugarcane CBC. The raw sequences were assembled into 205,411 CCSs that were filtered by coverage (≥2×) and reliability. We considered a CCS as reliable when it was above a threshold of similarity to a sequence deposited in a curated database and/or above a threshold of similarity to any other sequence of the CBC dataset (Armanhi et al., 2016). After filtering, 80,959 CCSs were recovered, allowing the identification of the bacterial composition of 2,942 wells (Supplementary Table S3). Among these, 1,717 wells were obtained from colonies of rhizosphere enriched microbiota, 923 from endophytic root and 302 from endophytic stalk of sugarcane.

**Figure 1:**
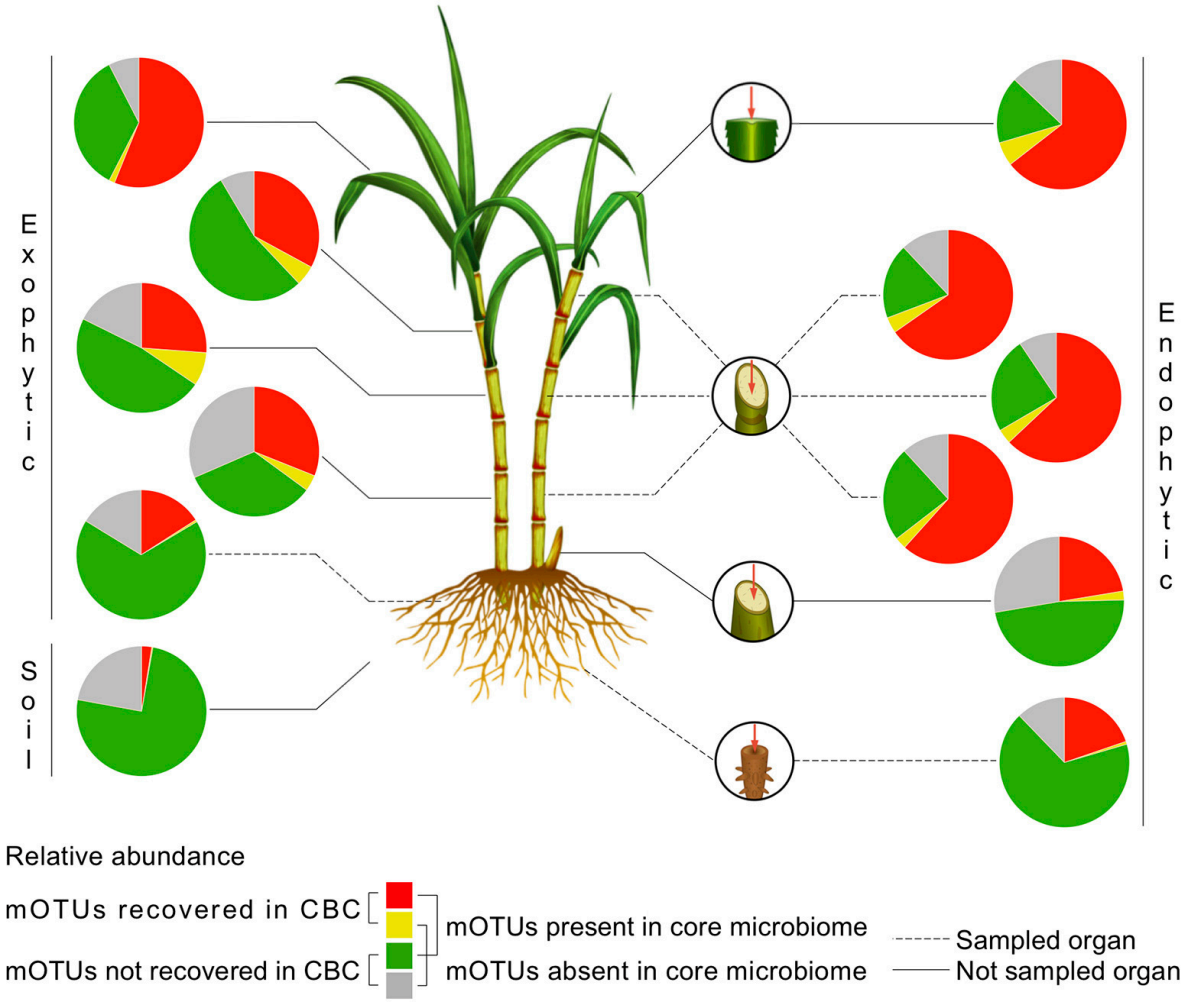
Estimated recovery of bacterial groups from sugarcane organs accessed by the CBC. Recovery estimates in terms of abundance for roots, stalks (bottom, medium, and upper), and leaves were calculated by cross-referencing the CBC with the sugarcane community profile. Pie charts separately display the relative abundance of mOTUs (OTUs obtained by community assemblage analysis of the sugarcane microbiome). The CBC contains representative sets of bacterial members of the sugarcane core microbiomes, stable, and highly abundant microbial communities with putative impact on plant development.

To investigate whether the sugarcane CBC targeted a representative set of the sugarcane microbiome, we cross-referenced the CBC with the sugarcane microbiome profile (de Souza et al., 2016). We found that 48, 40, and 33 cOTUs from the CBC are representatives of groups that colonize the endophytic bottom, medium and upper stalk, respectively. These cOTUs matched a total of 66, 56, 48 mOTUs from the sugarcane microbiome and accounted for 61.6, 63, 65.3% of the total relative abundance in the core microbiomes of these respective organs. Although the microbial diversity is higher in the root compartments than other plant organs (de Souza et al., 2016), the sugarcane CBC recovered 75 cOTUs that matched 128 mOTU in the sugarcane microbiome and accounted for 15.9% of relative abundance in the rhizosphere core microbiome. The microbes from the leaves were not sampled for the microbial collection, however, the culture collection targeted unrelated representatives that accounted for 56.1 and 64.5% of the exophytic and endophytic leaf core microbiomes, respectively (Figure 1 and Supplementary Table S4).

### Bacterial composition of the sugarcane CBC plate wells

**Figure 2:**
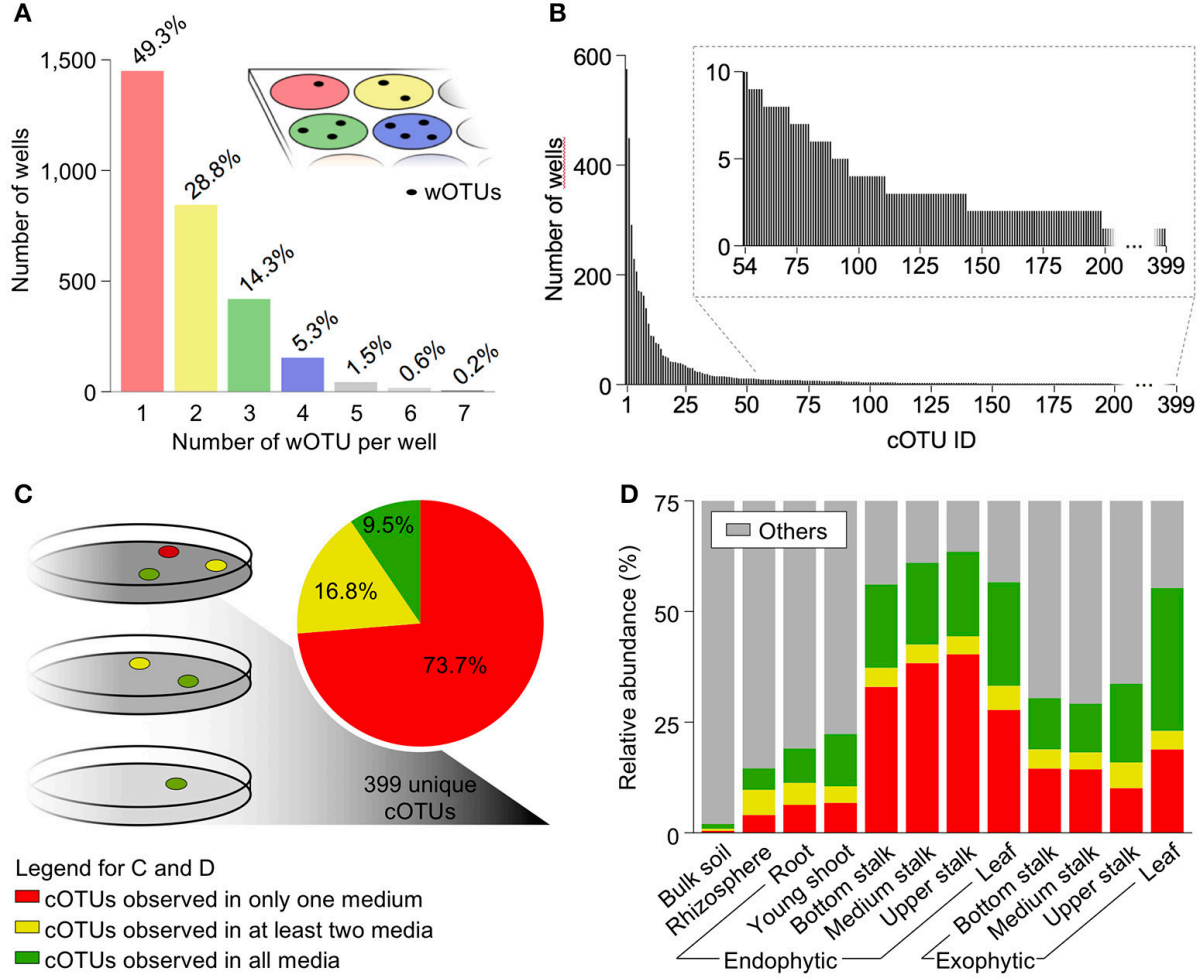
Characterization of OTU composition from isolated communities in sugarcane CBC. **(A)** Number of OTUs per well (wOTUs) as determined by clustering sequences within wells. **(B)** Redundancy of OTUs in the CBC determined by clustering wOTU sequences into cOTUs. The cOTUs represent unique OTUs in the CBC. **(C)** cOTU preference for culture media. Results are derived by cOTUs that were grown in only one medium, two media, or all used culture media. **(D)** Relative abundance of cOTUs per their preference for culture medium.

The bacterial composition of each CBC plate well were accessed by individually clustering the CCSs from each well into wOTUs. This analysis revealed that 1,450 wells (49.3%) contained single bacterial wOTUs, and the remaining 1,492 wells (50.7%) harbored multiple bacterial wOTUs (Figure 2A). The clustering of the wOTU sequences revealed a total of 399 unique cOTUs. The most redundant cOTU was present in 575 CBC wells. Conversely, 303 of the 399 cOTUs were found in less than five wells, and among these, 200 were found in single wells of the entire culture collection (Figure 2B and Supplementary Table S5).

We then investigated if the nutrient composition of the different culture media was selective for some bacterial groups. Among the 399 unique cOTUs, only 38 (9.5%) grew in all culture media. Contrastingly, 294 cOTUs (74%) showed culture media specificity, with 160 growing only in half-strength LB supplemented with sugarcane juice at 8 g l^−1^ TRS, 103 growing in half-strength LB supplemented with sugarcane juice at 35 g l^−1^ TRS, and 31 growing in only YPD medium (Figure 2C). The bottom and medium stalk bacterial groups that grew in a single culture medium showed higher relative abundance compared to those growing in all media. Interestingly, more than one-third of the total stalk bacterial endophytes grew specifically in one of the three culture media used, regardless of the type of media. Contrastingly, in both exophytic upper stalk and top leaves, the microbes that grew in all media were those which together presented a higher relative abundance (Figure 2D).

**Figure 3:**
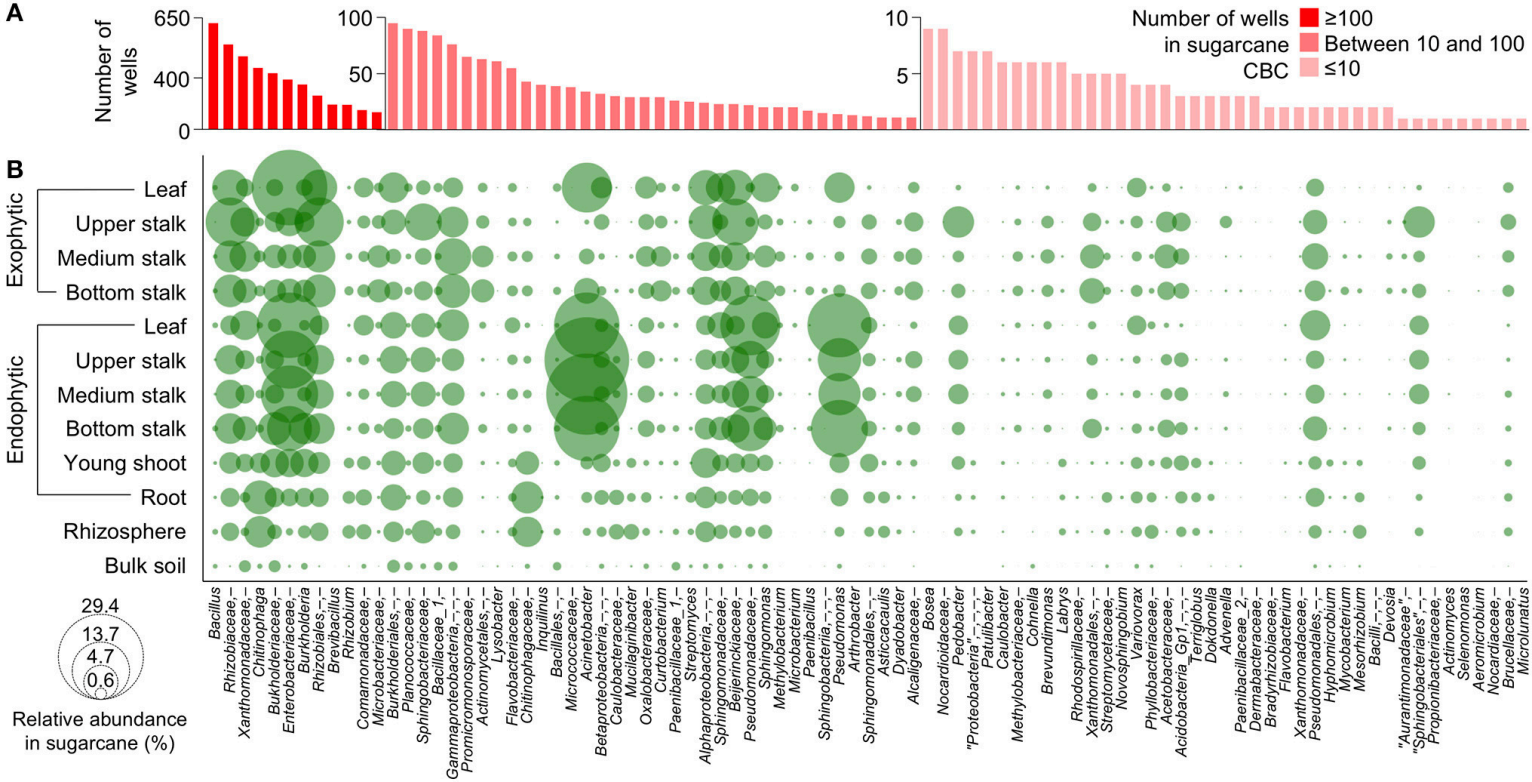
Correlation of genera relative abundance in plant organs and their occurrence in culture media. **(A)** The occurrence of genera in the sugarcane community-based culture collection (CBC) calculated by the number of wells containing at least one member of a given genus. **(B)** Relative abundance of genera in sugarcane roots, stalks (bottom, medium, and upper), and leaves of sugarcane based on their community profiles. Genera are represented by green circles, and circle sizes are proportional to the relative abundance of genera in a given organ. Taxonomic levels considered “unknown” were collapsed to the deepest common levels, which are comma-separated, and “unknown” taxa are represented as “−”.

The correlation between the relative abundances of the bacterial groups of the sugarcane CBC with those in the sugarcane organs (de Souza et al., 2016) was investigated by grouping cOTUs at the deepest known taxonomic level and displaying them according to their number of representatives in the CBC and their relative abundance in the sugarcane microbiome. Overall, a positive correlation was maintained, as the most represented groups in the CBC were also present at high abundances in the sugarcane organs. For example, members of the families *Rhizobiaceae, Xanthomonadaceae, Burkholderiaceae*, and *Enterobacteriaceae*, which are highly abundant in the sugarcane organs, were also highly redundant in the CBC, although *Acinetobacter* and *Pseudomonas*, which are highly abundant in sugarcane organs, were less represented in the CBC (Figure 3 and Supplementary Table S6).

### CBC representation of the sugarcane microbiome

**Figure 4:**
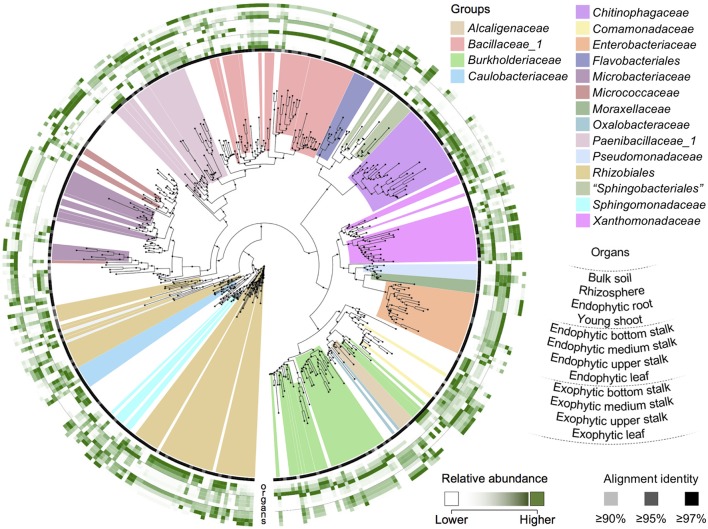
Phylogenetic tree of cOTUs of the sugarcane CBC. Outer rings show OTU relative abundance in sugarcane organs by color scale, from white (lower abundance) to strong green (higher abundance). Microbial groups representing the sugarcane core microbiome were highlighted for their putative beneficial activities or organ-colonization features. The relative abundances of cOTUs in each organ were colored from the lowest to the highest value for a given cOTU.

The predominant phylum in the CBC was “*Proteobacteria”*, found in 2,106 wells (Table 1). The representatives of families *Rhizobiaceae, Xanthomonadaceae, Burkholderiaceae, Enterobacteriaceae, Comamonadaceae, Caulobacteraceae, Sphingomonadaceae, Moraxellaceae, Pseudomonadaceae, Oxalobacteraceae*, and *Bradyrhizobiaceae* were found among this phylum (Figure 4). At the family level, *Rhizobiaceae* was the second most represented family (found in 634 wells), followed by *Burkholderiaceae* and *Xanthomonadaceae*, found in 539 and 482 wells, respectively. *Firmicutes* is the second most represented phylum in the CBC (found in 832 wells). Within this phylum, 661 wells contained members of *Bacillaceae_1*, followed by *Paenibacillaceae_1*, identified in 184 wells. “*Bacteroidetes”*, “*Actinobacteria”*, and “*Acidobacteria”* are the less represented phyla in the CBC. Interestingly, from the 566 wells identified as “*Bacteroidetes”*, 389 wells harbored members of the family *Chitinophagaceae*. Within this phylum, *Sphingobacteriaceae* and *Flavobacteriaceae* families were identified in 122 and 56 wells, respectively. CBC also harbors 146 wells containing members of the family *Microbacteriaceae*, included in the phylum “*Actinobacteria”* (Figure 4 and Table 1).

**Table 1:** Taxonomic prediction and number of wells of wOTUs per taxonomic level in the sugarcane CBC.

**Phylum**	**No. of wells**	**Class**	**No. of wells**	**Order**	**No. of wells**	**Family**	**No. of wells**	**Genus**	**No. of well**
*Acidobacteria*	6	*Acidobacteria_Gp1*	6	u_*Acidobacteria_Gp1*	6	u_*Acidobacteria_Gp1*	6	Terriglobus	3
								u_*Acidobacteria_Gp1*	3
*Actinobacteria*	353	*Actinobacteria*	353	*Actinomycetales*	346	*Actinomycetaceae*	1	*Actinomyces*	1
						*Dermabacteraceae*	3	u_*Dermabacteraceae*	3
						*Intrasporangiaceae*	3	u_*Intrasporangiaceae*	3
						*Microbacteriaceae*	146	*Curtobacterium*	29
								*Microbacterium*	20
								u_*Microbacteriaceae*	100
						*Micrococcaceae*	50	*Arthrobacter*	13
								u_*Micrococcaceae*	38
						*Mycobacteriaceae*	2	*Mycobacterium*	2
						*Nocardiaceae*	1	u_*Nocardiaceae*	1
						*Nocardioidaceae*	10	*Aeromicrobium*	1
								u_*Nocardioidaceae*	9
						*Promicromonosporaceae*	65	u_*Promicromonosporaceae*	65
						*Propionibacteriaceae*	2	*Microlunatus*	1
								u_*Propionibacteriaceae*	1
						*Streptomycetaceae*	29	*Streptomyces*	25
								u_*Streptomycetaceae*	5
						u_*Actinomycetales*	63	u_*Actinomycetales*	63
				*Solirubrobacterales*	8	*Patulibacteraceae*	8	*Patulibacter*	7
								u_*Patulibacteraceae*	1
				u_*Actinobacteria*	1	u_*Actinobacteria*	1	u_*Actinobacteria*	1
*Bacteroidetes*	566	*Cytophagia*	11	*Cytophagales*	11	*Cytophagaceae*	11	*Dyadobacter*	11
		*Flavobacteriia*	57	“*Flavobacteriales”*	57	*Flavobacteriaceae*	56	*Flavobacterium*	2
								u_*Flavobacteriaceae*	55
						u_“*Flavobacteriales”*	1	u_“*Flavobacteriales”*	1
		*Sphingobacteriia*	511	“*Sphingobacteriales”*	496	*Chitinophagaceae*	389	*Chitinophaga*	359
								u_*Chitinophagaceae*	43
						*Sphingobacteriaceae*	122	*Mucilaginibacter*	29
								*Pedobacter*	7
								u_*Sphingobacteriaceae*	88
						u_“*Sphingobacteriales”*	1	u_“*Sphingobacteriales”*	1
				u_*Sphingobacteriia*	15	u_*Sphingobacteriia*	15	u_*Sphingobacteriia*	15
*Proteobacteria*	2,106	*Alphaproteobacteria*	941	*Caulobacterales*	51	*Caulobacteraceae*	51	*Asticcacaulis*	11
								*Brevundimonas*	6
								*Caulobacter*	6
								u_*Caulobacteraceae*	30
				*Rhizobiales*	818	“*Aurantimonadaceae”*	1	u_“*Aurantimonadaceae”*	1
						*Beijerinckiaceae*	23	u_*Beijerinckiaceae*	23
						*Bradyrhizobiaceae*	10	*Bosea*	9
								u_*Bradyrhizobiaceae*	2
						*Brucellaceae*	1	u_*Brucellaceae*	1
						*Hyphomicrobiaceae*	4	*Devosia*	2
								*Hyphomicrobium*	2
						*Methylobacteriaceae*	23	*Methylobacterium*	20
								u_*Methylobacteriaceae*	6
						*Phyllobacteriaceae*	6	*Mesorhizobium*	2
								u_*Phyllobacteriaceae*	4
						*Rhizobiaceae*	634	*Rhizobium*	143
								u_*Rhizobiaceae*	496
						*Xanthobacteraceae*	8	*Labrys*	6
								u_*Xanthobacteraceae*	2
						u_*Rhizobiales*	197	u_*Rhizobiales*	197
				*Rhodospirillales*	45	*Acetobacteraceae*	4	u_*Acetobacteraceae*	4
						*Rhodospirillaceae*	41	*Inquilinus*	40
								u_*Rhodospirillaceae*	5
				*Sphingomonadales*	60	*Sphingomonadaceae*	48	*Novosphingobium*	5
								*Sphingomonas*	20
								u_*Sphingomonadaceae*	23
						u_*Sphingomonadales*	12	u_*Sphingomonadales*	12
				u_*Alphaproteobacteria*	24	u_*Alphaproteobacteria*	24	u_*Alphaproteobacteria*	24
		*Betaproteobacteria*	754	*Burkholderiales*	739	*Alcaligenaceae*	14	*Advenella*	3
								u_*Alcaligenaceae*	11
						*Burkholderiaceae*	539	*Burkholderia*	262
								*Pandoraea*	1
								u_*Burkholderiaceae*	328
						*Comamonadaceae*	118	*Delftia*	1
								*Variovorax*	4
								u_*Comamonadaceae*	113
						*Oxalobacteraceae*	29	u_*Oxalobacteraceae*	29
						u_*Burkholderiales*	95	u_*Burkholderiales*	95
				u_*Betaproteobacteria*	32	u_*Betaproteobacteria*	32	u_*Betaproteobacteria*	32
		*Gammaproteobacteria*	869	“*Enterobacteriales”*	291	*Enterobacteriaceae*	291	u_*Enterobacteriaceae*	291
				*Pseudomonadales*	69	*Moraxellaceae*	34	*Acinetobacter*	34
								u_*Moraxellaceae*	1
						*Pseudomonadaceae*	34	*Pseudomonas*	14
								u_*Pseudomonadaceae*	22
						u_*Pseudomonadales*	2	u_*Pseudomonadales*	2
				*Xanthomonadales*	486	*Xanthomonadaceae*	482	*Dokdonella*	3
								*Lysobacter*	61
								u_*Xanthomonadaceae*	427
						u_*Xanthomonadales*	5	u_*Xanthomonadales*	5
				u_*Gammaproteobacteria*	76	u_*Gammaproteobacteria*	76	u_*Gammaproteobacteria*	76
		u_“*Proteobacteria”*	7	u_“*Proteobacteria”*	7	u_“*Proteobacteria”*	7	u_“*Proteobacteria”*	7
*Firmicutes*	832	*Bacilli*	831	*Bacillales*	830	*Bacillaceae_1*	661	*Bacillus*	620
								u_*Bacillaceae_1*	84
						*Paenibacillaceae_1*	184	*Brevibacillus*	145
								*Cohnella*	6
								*Paenibacillus*	17
								u_*Paenibacillaceae_1*	26
						*Paenibacillaceae_2*	3	u_*Paenibacillaceae_2*	3
						*Planococcaceae*	90	u_*Planococcaceae*	90
						*Staphylococcaceae*	2	*Staphylococcus*	2
						u_*Bacillales*	39	u_*Bacillales*	39
				u_*Bacilli*	2	u_*Bacilli*	2	u_*Bacilli*	2
		*Clostridia*	1	u_*Clostridia*	1	u_*Clostridia*	1	u_*Clostridia*	1
		*Negativicutes*	1	*Selenomonadales*	1	*Veillonellaceae*	1	*Selenomonas*	1
u_*Bacteria*	1	u_*Bacteria*	1	u_*Bacteria*	1	u_*Bacteria*	1	u_*Bacteria*	1

*Taxonomic prediction was obtained using “utax” in USEARCH and RDP database. Taxon names were kept the same as described in the database. Prefix “u_” was added when prediction is below the minimum confidence score of 0.9*.

### Probing the plant beneficial impact of an abundance-based synthetic community assembled from the sugarcane CBC

**Figure 5:**
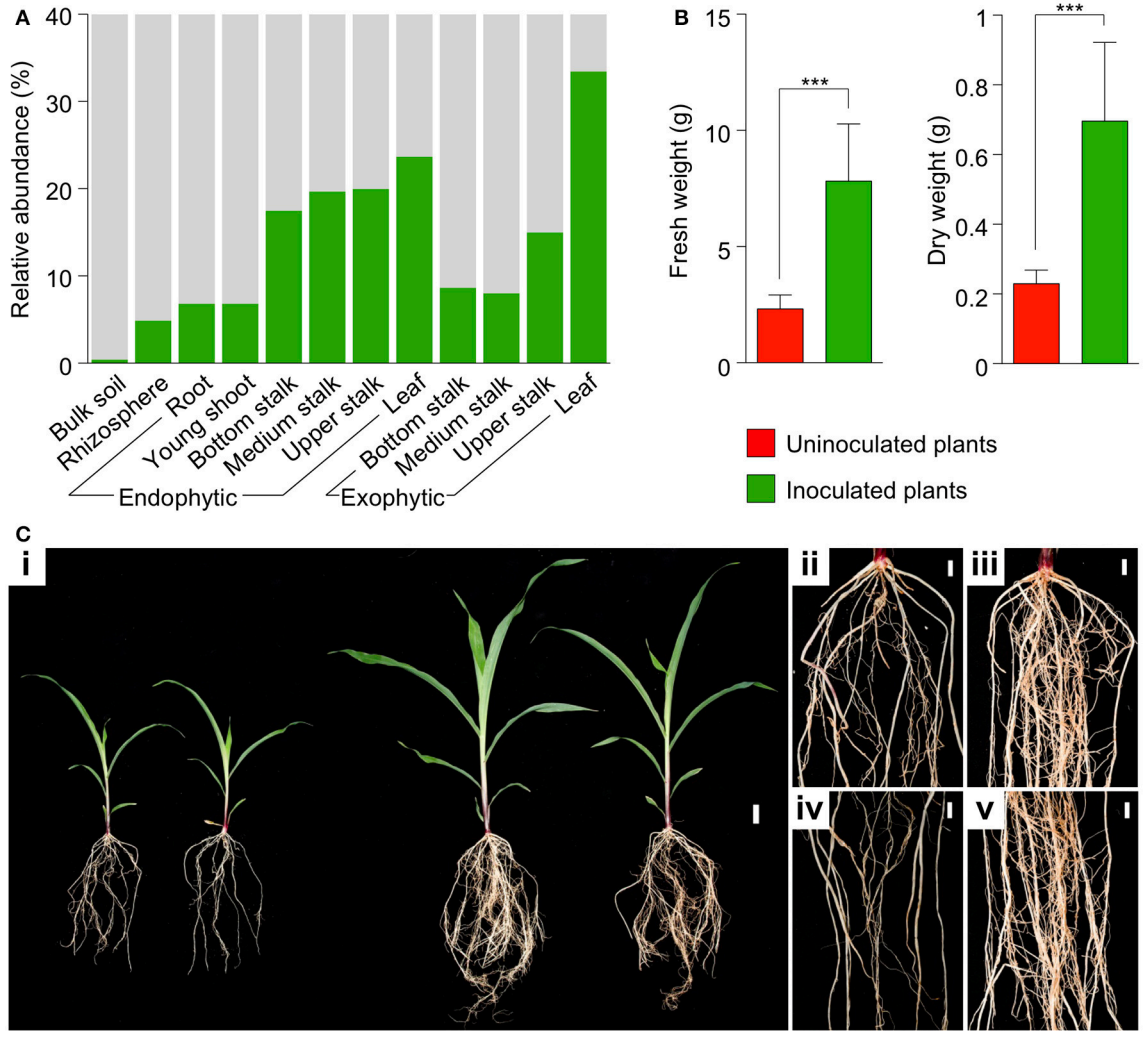
A synthetic community composed of dominant microbial groups isolated from sugarcane induces maize plant growth. **(A)** Relative abundance of the microbial groups comprising members of the synthetic community. **(B)** Effect of inoculation in fresh and dry weight of maize seedlings after 4 weeks of growth. Inoculated plants showed more than three times the biomass compared with uninoculated plants. **(C)** Plant growth and biomass of plants treated with a nutrient solution in the absence (**i**, left) or presence (**i**, right) of the synthetic community. The effect on root growth in uninoculated plants **(ii,iv)** and inoculated plants **(iii,v)**. ^***^p ≤ 0.001. Scale bars: 3 cm **(i)** and 1 cm **(ii–v)**.

A synthetic community was assembled by using 17 wells of the sugarcane CBC comprising 20 OTUs with the top high relative abundances in the sugarcane root and stalk core microbiomes (de Souza et al., 2016) (Supplementary Figure S5). These OTUs accounted for 4.9 and 6.8% of the rhizosphere and endophytic root, and 17.5–20% of the sugarcane endophytic stalk core microbiomes, respectively (Figure 5A). The abundance-based synthetic community was inoculated in maize plants grown in vermiculite and irrigated with Hoagland's nutrient solution. The inoculated plants increased their fresh weight by 3.4 × (average of 7.82 g per plant) compared with uninoculated plants (2.31 g per plant). Similar differences were observed for the plant dry weights (0.7 g per plant for inoculated and 0.23 g per plant for uninoculated plants; Figure 5B and Supplementary Table S7). Compared with uninoculated plants, the inoculated ones were more vigorous, with dark green leaves and presented increased branched root systems with an increased number of lateral roots (Figure 5C).

### Members of the abundance-based synthetic community efficiently colonizes plants and displaces its natural microbiota

**Figure 6:**
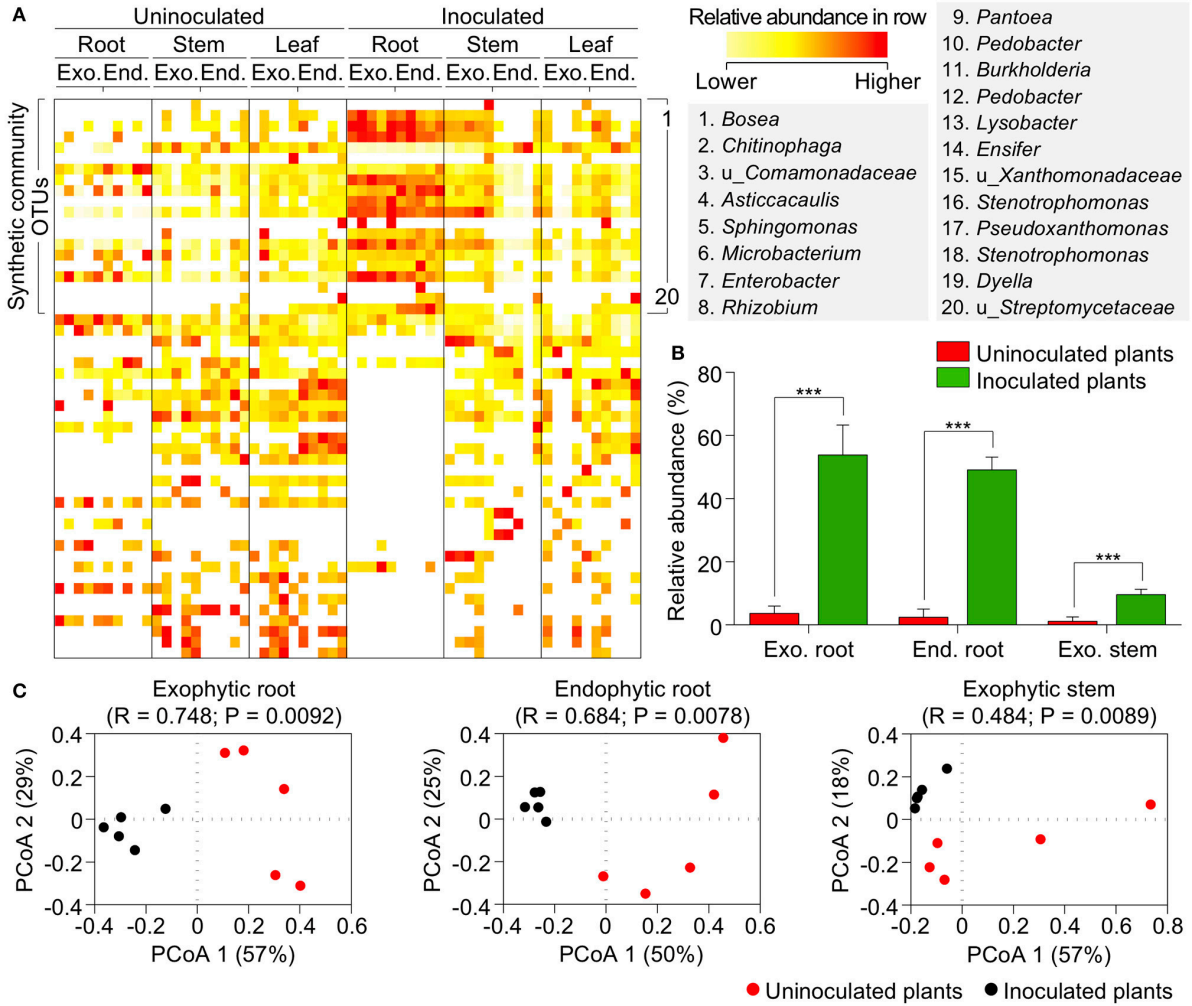
The synthetic community robustly colonizes maize plants and displaces the natural OTU relative abundance pattern. **(A)** Heatmap of relative abundances of OTUs found in organs of uninoculated and inoculated plants. Color range is given by row. OTUs in the synthetic community are highlighted, and their taxonomic prediction is shown on the right. The complete heatmap is shown in Supplementary Figure S6. **(B)** Relative abundance of OTUs present in the synthetic community in maize inoculation. **(C)** Principal coordinates analysis (PCoA) of Bray–Curtis dissimilarity matrix of exophytic and endophytic roots and exophytic stem tissues of inoculated and uninoculated plants. Exo, exophytic; End, endophytic; u_, unknown taxa. ^***^p ≤ 0.001.

The colonization pattern of the synthetic community was verified by 16S sequencing of samples from exophytic and endophytic compartments of roots, stems, and leaves of inoculated and uninoculated plants. A significant shift in the community profile was observed in exophytic and endophytic roots and exophytic stems of inoculated as compared with the uninoculated plants (Figure 6A). The OTUs identification is shown in Supplementary Table S8. The synthetic community bacteria *Asticcacaulis, Burkholderia, Chitinophaga, Ensifer, Lysobacter, Pedobacter, Rhizobium, Stenotrophomonas*, and two unknown genera of the families *Comamonadaceae* and *Streptomycetaceae* efficiently colonized and became predominant in the roots and stems of inoculated plants. Interestingly, 10 bacteria of the synthetic inoculant, although highly abundant in the sugarcane core microbiome, did not robustly colonize the maize plants. The natural microbiota of the uninoculated plants contained OTUs that were classified in the same groups as those of the synthetic community. However, the total abundance of these OTUs was minimal compared with that of the inoculated plants (3.7, 2.4, and 1.2% for uninoculated plants against 53.9, 49.1, and 9.6% for inoculated plants exophytic root, endophytic root, and exophytic stem, respectively; Figure 6B). Natural microbiota was considered as the communities naturally acquired by the plant from the environment. The microbiota of the uninoculated plants was more diverse and widespread among groups. The results led to the conclusion that the OTUs of the abundance-based synthetic inoculant displaced the OTUs of the natural microbiota and changed the bacterial profiling both in diversity and abundance compared with the uninoculated plants (Figure 6A and Supplementary Figure S6). Of the 20 OTUs of the synthetic community, 10 were identified as efficient colonizers based on their abundance (Kruskal–Wallis test, *P* < 0.05) in the roots of inoculated plants compared with uninoculated plants. These robust colonizers accounted for 53.9 and 49.1% of the total relative abundance in exophytic and endophytic roots, respectively. Four OTUs from the synthetic community efficiently colonized the exophytic stem (Kruskal–Wallis test, *P* < 0.05) and accounted for 9.6% of the total abundance in this organ (Figure 6B).

Despite none of the synthetic community OTUs displayed differential abundances in the leaves of inoculated plants, they displaced several abundant, naturally occurring bacterial groups in this organ (Figure 6A and Supplementary Figure S6). This adjustment in the community profile of the inoculated plants was corroborated by principal coordinates analysis (PCoA) of Bray–Curtis dissimilarity matrix. The inoculated plants clearly formed distinct clusters, which could be observed in both compartments of roots (ANOSIM; *R* = 0.748, *P* < 0.01 and *R* = 0.684, *P* < 0.01 for exophytic and endophytic, respectively) and exophytic stems (ANOSIM; *R* = 0.484, *P* < 0.01; Figure 6C). Inoculation was the first principal coordinate in the roots and the second in the stems, explaining over 50 and 18% of differences between the samples, respectively. This pattern was not observed for the endophytic stems and leaves (Supplementary Figure S7).

## Discussion

### The use of CBC for microbial discovery

Sugarcane microbiota has long been investigated for its potential beneficial impact on plant growth and development. However, the knowledge of the potential role of the sugarcane microbiota has been restricted to the characterization of diazotrophic bacteria discovered by selective culture media (Döbereiner, 1961; Döbereiner et al., 1972; Cavalcante and Döbereiner, 1988; Olivares et al., 1996). We recently revealed, in sugarcane, highly abundant microbial groups whose functional role in plant remains to be determined (de Souza et al., 2016). We also developed the CBC method to construct microbial culture collections (Armanhi et al., 2016) and used this strategy to identify and annotate our entire sugarcane culture collection described here.

The use of sterile sugarcane juice as an additive in the culture medium has been shown to improve the growth of bacterial groups adapted to the sugars, amino acids, vitamins, organic acids, and other small molecules (Gupta and Mukerji, 2002; Uren, 2007; Kim and Day, 2011). Some bacterial groups such as *Pseudomonas, Flavobacterium, Agrobacterium*, and *Enterobacter*, for instance, have been shown to respond to rhizosphere exudates (Curl and Truelove, 1986; Taghavi et al., 2015) that directly affect bacterial growth (Grayston et al., 1998; Hadacek and Kraus, 2002; Singh and Mukerji, 2006). By using culture media supplemented with sterile sugarcane juice, we retrieved microbial composition and taxonomic classification of 2,942 wells resulting in the identification of 399 unique OTUs. The CBC redundancy may have occurred due to (1) the high abundance of some bacterial groups in the organ core microbiome or (2) an unintentional imposed selection by the culture media used.

Bacterial culture collections have been constructed from the environment or human-derived samples by using defined culture media (Goodman et al., 2011; Paungfoo-Lonhienne et al., 2014; Bai et al., 2015; Browne et al., 2016). In one case, the use of a single broad-spectrum medium allowed targeting a large proportion of the vast gut bacterial diversity (Browne et al., 2016). In the present study, the use of three broad-spectrum culture media revealed that most identified OTUs comprise bacteria that grow specifically in a single medium. Only a small proportion of the OTUs thrived in all three media. The use of YPD aimed to target bacteria, indigenous yeasts and other fungi inhabiting sugarcane stalks (da Silva-Filho et al., 2005; Basso et al., 2008; de Souza et al., 2016). YPD is a conventional fungal culture medium which allowed growth of some specific bacterial groups that may coexist with fungi in some of the wells, perhaps reflecting their natural environment.

### The sugarcane CBC recovered relevant bacterial groups

Culturing representatives of the microbial diversity from host-associated and natural ecosystems is challenging (Bulgarelli et al., 2012, 2013, 2015; Lundberg et al., 2012; Edwards et al., 2015; Panke-Buisse et al., 2015; Zarraonaindia et al., 2015; Coleman-Derr et al., 2016; Castrillo et al., 2017). It is recognized that there is a gap between the diversity found in cultured microbes and the full community profiles (Goodman et al., 2011; Lebeis et al., 2012). Thus, a critical step in establishing extensive cultivation of microorganisms is to validate the extent of the microbial composition recovered from microbiomes. While in humans substantial portions from the gut microbiome have been recovered (Goodman et al., 2011; Browne et al., 2016), in crops this is still a poorly explored topic, despite the fact that many bacterial groups have been shown to contain cultivable members in plants (Bai et al., 2015). Our results demonstrate that the use of the CBC method (Armanhi et al., 2016) allowed the recover of bacterial groups accounting for 15.9–65.3% of the core microbiomes of roots, stalks, and leaves of sugarcane. Most of the identified OTUs belong to highly abundant bacterial phyla inhabiting the sugarcane organs (de Souza et al., 2016).

The most represented phylum in the CBC was “*Proteobacteria”*, whose members have been shown to promote plant growth by diverse mechanisms (Pisa et al., 2011; Yuan et al., 2011; Beneduzi et al., 2013). Members of the order *Rhizobiales* (*Alphaproteobacteria* class), found in the CBC, belong to groups of bacteria with representatives capable of nitrogen fixation (Carvalho et al., 2010; Gnat et al., 2015; Melorose et al., 2015) and soil denitrification (Yoshida et al., 2012). Moreover, the genus *Burkholderia* (*Betaproteobacteria* class), comprises endophytic bacteria (Perin et al., 2006; Luvizotto et al., 2010; Beneduzi et al., 2013) that has been shown to be involved in the denitrification process (Yoshida et al., 2012). The CBC representatives of the family *Burkholderiaceae* are especially abundant in young shoots and bottom stalks (de Souza et al., 2016). Beyond *Burkholderia*, the order *Burkholderiales* includes the genus *Herbaspirillum* (*Oxalobacteraceae* family), well known for its nitrogen fixation capacity (Pimentel et al., 1991; Olivares et al., 1996). The sugarcane CBC targeted a member of the family *Moraxellaceae* (*Gammaproteobacteria* class), the genus *Acinetobacter*, which presents members that have long been studied for its nitrogen fixation ability, phytohormone production and mineral solubilization (Rokhbakhsh-Zamin et al., 2011; Yuan et al., 2011). Members of the family *Enterobacteriaceae*, such as the genera *Erwinia* and *Enterobacter*, are also well known as plant growth promoters (Loiret et al., 2004; Yuan et al., 2011). Within the phylum “*Bacteroidetes”, Chitinophagaceae* is a lesser known bacterial family, but one of the most abundant in the sugarcane root core microbiome (de Souza et al., 2016). Over half of the CBC wells containing this phylum belong to the family *Chitinophagaceae*. This phylum also includes *Flavobacterium*, a genus that has been isolated from the sugarcane leaves and demonstrated to fix nitrogen and produce phytohormones (Fischer et al., 1993; Yuan et al., 2011). The CBC also targeted the genera *Microbacterium* and *Curtobacterium* (“*Actinobacteria”* phylum), and the phylum “*Acidobacteria”*, which includes members with plant-beneficial activities (Anandham et al., 2008; Magnani et al., 2010; de Pereira et al., 2012; Qin et al., 2014; Kielak et al., 2016).

Overall, most CBC representatives have their organ preference consistent with their abundance in sugarcane organs. This assumption could be observed particularly for *Moraxellaceae, Pseudomonadaceae*, and *Enterobacteriaceae* in the endophytic compartment of stalks and leaves, for *Burkholderiaceae* in the young shoots and endophytic bottom stalks, and for *Xanthomonadaceae* in the exophytic stalks (de Souza et al., 2016). As observed in the sugarcane microbiome, leaves are the preferred organ for the cultivable bacteria of the family *Sphingomonadaceae* (de Souza et al., 2016). Although representatives of the family *Rhizobiaceae* are enriched in exophytic stalk and leaf core microbiomes (de Souza et al., 2016), their CBC representatives are mostly found in roots and young shoots.

We also determined the organ preferences of the OTU clusters of the CBC formed by closely related taxa. *Moraxellaceae, Pseudomonadaceae*, and *Enterobacteriaceae* are phylogenetically close groups and they showed endophytic stalks and leaves as their preferred organs (de Souza et al., 2016). Thus, it seems that some phylogenetically close bacterial groups, might have similar preferred organs while other groups may not follow the same rule. For example, the CBC representatives of *Xanthomonadaceae* are bacteria with contrasting organ preferences, mostly found in the exophytic compartment of stalks (de Souza et al., 2016). The order *Rhizobiales* and families *Caulobacteraceae* and *Sphingomonadaceae* are closely related groups, although they did not present the same colonization preference of sugarcane organs. However, upon close inspection, we observed subsets of OTUs within these groups that show similar preferred organs. The limitations imposed by the existing 16S rRNA gene sequence databases did not allow the classification of some of the OTUs at a generic or specific level; therefore, our findings indicate that phylogenetic distance might not be directly related to organ-preferences. This observation may suggest that while beneficial plant functions could be conserved among the members of some of the closely related taxa, within families such as *Pseudomonadaceae, Moraxellaceae, Chitinophagaceae*, and *Microbacteriaceae*, the consistent preferences appeared within just a small subset of representatives of other groups. Identifying these functions and correlations may help understand the plant-microbial community association.

### Abundant microbial groups from the sugarcane microbiome have a high impact on plant growth promotion in maize

Traditionally, beneficial microbes are isolated based on screening for known plant growth-promoting (PGP) related activities or taxonomic affiliation. However, there is no evidence that a pre-existing PGP activity or taxonomic affiliation is correlated to the representativeness of a microbial group in plant organs. In fact, in sugarcane, the sole use of these strategies have targeted minor components of the microbial diversity (de Souza et al., 2016). In this work, we chose to select microbial groups based on the community profile and assemblage pattern targeting neglected microbial groups from the sugarcane microbiome. A synthetic bacterial community was assembled by selecting naturally dominant groups in the sugarcane microbiome regardless of pre-existing traits or taxonomic affiliation.

Recently, synthetic communities have been designed as inoculants to study colonization and beneficial impacts on growth and development (Bai et al., 2015; Lebeis et al., 2015; Niu et al., 2017). Synthetic communities have the advantage of carrying redundant functions together with individual contributions of specific functions. Collectively, these functions could be advantageous for the plant, which would explain why different bacterial groups are kept at high abundance during plant development.

We evaluated the effect of the abundance-based synthetic community using maize as a crop model. Maize was selected as a candidate crop because it is phylogenetically close to sugarcane. The synthetic inoculant produced a remarkable effect on plant growth. Bacterial members of the synthetic community efficiently colonized exophytic and endophytic roots and exophytic stems of maize plants in an organ-preferred pattern similar to that observed for the sugarcane plant (de Souza et al., 2016). Notably, the 16S rRNA gene sequences of naturally occurring bacteria in uninoculated plants were clustered in the same groups (OTUs) of the synthetic community. However, these bacteria were less efficient in colonizing and promote plant growth and development. This result suggests that the abundance-based synthetic community has plant beneficial functions that are absent in the maize natural microbiota. These functions may arise from genomic scale differential expression and genetic polymorphism at the protein level, established throughout the microbiome/plant co-evolution and could explain its superiority over the maize resident plant-borne microbiome. This hypothesis is in keeping with previous studies describing that microorganisms from the same species confer different beneficial effects on plant survival depending on their habitat, which has been described as habitat adapted-symbioses (Rodriguez et al., 2008).

The robustness of colonization of maize plants by members of the abundance-based synthetic community displaced the naturally occurring maize OTUs in the inoculated plants. The displacement of natural microbiota may include bacteria that somehow negatively impact plant growth and development. Interestingly, the microbial displacement also occurred in plant organs that have not been robustly colonized by the members of the synthetic community. This effect can be seen in leaves and suggests that the synthetic inoculant might also affect microbial assemblage by factors other than local competition against the natural community and could be related to plant differential gene expression induced by the plant-microbe interaction.

It is remarkable that the abundance-based synthetic community assembled from the sugarcane microbiome had a tremendous impact on maize plant growth. The results suggest that selection based on relative abundance in the plant microbiome could be used for the identification of beneficial microorganisms with a broader impact cross-species.

## Conclusions

The guided use of microorganisms to promote plant growth, development and health have a great potential for agriculture sustainability. The establishment of plant-derived culture collections is key for the exploration of the biological potential of microbial communities. The creation of the sugarcane CBC allowed the isolation of bacterial groups belonging to diverse groups that have been understudied. The cross-referencing of microbial data of the culture collection with the microbiome profile of sugarcane was key to identify highly abundant naturally occurring bacteria from the core microbiome of the plant organs. This strategy allowed the assemblage of an abundance-based synthetic community that was probed as an inoculant using maize as a plant model. The fact that members of the abundance-based synthetic community robustly colonize the inoculated maize plants in an assemblage pattern similar to that naturally found in sugarcane plant organs is a significant finding. Additionally, the displacement of the naturally occurring maize microbiota and the dramatic impact on plant biomass and root stimulation is a sound proof of the concept for the abundance-based synthetic community for the assembly of plant inoculants. The present study is unique in describing a rational workflow for obtaining CBCs from plant or environmental samples and assess their relevance by cross-referencing with the microbiome data to find key microorganisms useful for sustainable agriculture.

## Author contributions

PA and JI: managed the project. PA, JI, and RdS: coordinated the project. JA, LdA, and RdS: performed plant organ sampling and microbiota isolation. JA and LdA: performed microbial plating, isolation and storage and isolated DNA from stored cultures. JA: prepared sequencing libraries of 16S rRNA gene for microbe identification of the CBC. JA and RdS: performed bioinformatics analyses. RdS: designed the inoculation experiments and prepared the synthetic community for inoculation assays. RdS, JA, and ND: performed the inoculation experiments and plant phenotyping. JA and RdS: prepared sequencing libraries for analyses of colonization in maize and performed bioinformatics analyses. JA: created figures with the help of RdS. JA: wrote the manuscript with significant input from RdS and PA. PA, JI, and RdS: critically revised the manuscript. All authors read and approved the final manuscript.

### Conflict of interest statement

The authors declare that the research was conducted in the absence of any commercial or financial relationships that could be construed as a potential conflict of interest. The reviewer YB and handling Editor declared their shared affiliation.

## Abbreviations

CBC: community-based culture collection
CCS: circular consensus sequence
cOTUs: collection-OTUs
mOTUs: microbiome-OTUs
OTU: operational taxonomic unit
TRS: total reducing sugars
wOTUs: well-OTUs.

## References

[B1] Anandham R. Indira Gandhi P. Madhaiyan M. Sa T. (2008). Potential plant growth promoting traits and bioacidulation of rock phosphate by thiosulfate oxidizing bacteria isolated from crop plants. J. Basic Microbiol. 48, 439–447. 10.1002/jobm.200700380 18785656

[B2] Armanhi J. S. L. de Souza R. S. C. de Araújo L. M. Okura V. K. Mieczkowski P. Imperial J.. (2016). Multiplex amplicon sequencing for microbe identification in community-based culture collections. Sci. Rep. 6:29543. 10.1038/srep29543 27404280 PMC4941570

[B3] Asnicar F. Weingart G. Tickle T. L. Huttenhower C. Segata N. (2015). Compact graphical representation of phylogenetic data and metadata with GraPhlAn. PeerJ 3:e1029. 10.7717/peerj.1029 26157614 PMC4476132

[B4] Bai Y. Müller D. B. Srinivas G. Garrido-Oter R. Potthoff E. Rott M.. (2015). Functional overlap of the *Arabidopsis* leaf and root microbiota. Nature 528, 364–369. 10.1038/nature16192 26633631

[B5] Barea J.-M. Pozo M. J. Azcón R. Azcán-Aguilar C. (2005). Microbial co-operation in the rhizosphere. J. Exp. Bot. 56, 1761–1778. 10.1093/jxb/eri197 15911555

[B6] Basso L. C. De Amorim H. V. De Oliveira A. J. Lopes M. L. (2008). Yeast selection for fuel ethanol production in Brazil. FEMS Yeast Res. 8, 1155–1163. 10.1111/j.1567-1364.2008.00428.x 18752628

[B7] Bastián F. Cohen A. Piccoli P. Luna V. Baraldi R. Bottini R. (1998). Production of indole-3-acetic acid and gibberellins A_1_ and A_3_ by *Acetobacter diazotrophicus* and *Herbaspirillum seropedicae* in chemically-defined culture media. Plant Growth Regul. 24, 7–11. 10.1023/A:1005964031159

[B8] Beneduzi A. Moreira F. Costa P. B. Vargas L. K. Lisboa B. B. Favreto R. (2013). Diversity and plant growth promoting evaluation abilities of bacteria isolated from sugarcane cultivated in the South of Brazil. Agric. Ecosyst. Environ. Appl. Soil Ecol. 63, 94–104. 10.1016/j.apsoil.2012.08.010

[B9] Brown S. D. Utturkar S. M. Klingeman D. M. Johnson C. M. Martin S. L. Land M. L.. (2012). Twenty-one genome sequences from *Pseudomonas* species and 19 genome sequences from diverse bacteria isolated from the rhizosphere and endosphere of *Populus deltoides*. J. Bacteriol. 194, 5991–5993. 10.1128/JB.01243-12 23045501 PMC3486089

[B10] Browne H. P. Forster S. C. Anonye B. O. Kumar N. Neville B. A. Stares M. D.. (2016). Culturing of “unculturable” human microbiota reveals novel taxa and extensive sporulation. Nature 533, 1–15. 10.1038/nature17645 27144353 PMC4890681

[B11] Bulgarelli D. Garrido-Oter R. Münch P. C. Weiman A. Dröge J. Pan Y.. (2015). Structure and function of the bacterial root microbiota in wild and domesticated barley. Cell Host Microbe 17, 392–403. 10.1016/j.chom.2015.01.011 25732064 PMC4362959

[B12] Bulgarelli D. Rott M. Schlaeppi K. van Themaat E. V. L. Ahmadinejad N. Assenza F.. (2012). Revealing structure and assembly cues for *Arabidopsis* root-inhabiting bacterial microbiota. Nature 488, 91–95. 10.1038/nature11336 22859207

[B13] Bulgarelli D. Schlaeppi K. Spaepen S. van Themaat E. V. L. Schulze-Lefert P. (2013). Structure and functions of the bacterial microbiota of plants. Annu. Rev. Plant Biol. 64, 807–838. 10.1146/annurev-arplant-050312-120106 23373698

[B14] Caporaso J. G. Kuczynski J. Stombaugh J. Bittinger K. Bushman F. D. Costello E. K.. (2010). QIIME allows analysis of high-throughput community sequencing data. Nat. Methods 7, 335–336. 10.1038/nmeth.f.303 20383131 PMC3156573

[B15] Carvalho F. M. Souza R. C. Barcellos F. G. Hungria M. Vasconcelos A. T. R. (2010). Genomic and evolutionary comparisons of diazotrophic and pathogenic bacteria of the order Rhizobiales. BMC Microbiol. 10:37. 10.1186/1471-2180-10-37 20144182 PMC2907836

[B16] Castrillo G. Teixeira P. J. Paredes S. H. Law T. F. de Lorenzo L. Feltcher M. E.. (2017). Root microbiota drive direct integration of phosphate stress and immunity. Nature 543, 513–518. 10.1038/nature21417 28297714 PMC5364063

[B17] Cavalcante V. A. Döbereiner J. (1988). A new acid-tolerant nitrogen-fixing bacterium associated with sugarcane. Plant Soil 108, 23–31. 10.1007/BF02370096

[B18] Cole J. R. Chai B. Farris R. J. Wang Q. Kulam S. A. McGarrell D. M.. (2005). The Ribosomal Database Project (RDP-II): sequences and tools for high-throughput rRNA analysis. Nucleic Acids Res. 33, 294–296. 10.1093/nar/gki038 15608200 PMC539992

[B19] Coleman-Derr D. Desgarennes D. Fonseca-Garcia C. Gross S. Clingenpeel S. Woyke T.. (2016). Plant compartment and biogeography affect microbiome composition in cultivated and native *Agave* species. New Phytol. 209, 798–811. 10.1111/nph.13697 26467257 PMC5057366

[B20] Curl E. A. Truelove B. (1986). The Rhizosphere. Berlin: Springer-Verlag.

[B21] Döbereiner J. Day J. M. Dart P. J. (1972). Nitrogenase activity in the rhizosphere of sugarcane and some other tropical grasses. Plant Soil 196, 191–196. 10.1007/BF01578494

[B22] Döbereiner J. (1961). Nitrogen-fixing bacteria of the genus *Beijerinckia* Derx in the rhizosphere of sugar cane. Plant Soil 15, 211–216. 10.1007/BF01400455

[B23] da Silva-Filho E. A. dos Santos S. K. Resende Ado M. de Morais Jo F. de Morais M. A. Simões D. A. (2005). Yeast population dynamics of industrial fuel-ethanol fermentation process assessed by PCR-fingerprinting. Antonie Van Leeuwenhoek 88, 13–23. 10.1007/s10482-005-7283-3 15928973

[B24] de Pereira G. V. M. Magalhães K. T. Lorenzetii E. R. Souza T. P. Schwan R. F. (2012). A multiphasic approach for the identification of endophytic bacterial in strawberry fruit and their potential for plant growth promotion. Microb. Ecol. 63, 405–417. 10.1007/s00248-011-9919-3 21837472

[B25] DeSantis T. Z. Hugenholtz P. Larsen N. Rojas M. Brodie E. L. Keller K.. (2006). Greengenes, a chimera-checked 16S rRNA gene database and workbench compatible with ARB. Appl. Environ. Microbiol. 72, 5069–5072. 10.1128/AEM.03006-05 16820507 PMC1489311

[B26] de Souza R. S. C. Okura V. K. Armanhi J. S. L. Jorrín B. Lozano N. da Silva M. J.. (2016). Unlocking the bacterial and fungal communities assemblages of sugarcane microbiome. Sci. Rep. 6:28774. 10.1038/srep28774 27358031 PMC4928081

[B27] Edgar R. (2016). SINTAX: a simple non-Bayesian taxonomy classifier for 16S and ITS sequences. bioRxiv 74161. 10.1101/074161

[B28] Edgar R. C. (2010). Search and clustering orders of magnitude faster than BLAST. Bioinformatics 26, 2460–2461. 10.1093/bioinformatics/btq461 20709691

[B29] Edgar R. C. (2013). UPARSE: highly accurate OTU sequences from microbial amplicon reads. Nat. Methods 10, 996–998. 10.1038/nmeth.2604 23955772

[B30] Edgar R. C. Flyvbjerg H. (2015). Error filtering, pair assembly and error correction for next-generation sequencing reads. Bioinformatics 31, 3476–3482. 10.1093/bioinformatics/btv401 26139637

[B31] Edwards J. Johnson C. Santos-Medellín C. Lurie E. Podishetty N. K. Bhatnagar S.. (2015). Structure, variation, and assembly of the root-associated microbiomes of rice. Proc. Natl. Acad. Sci. U.S.A. 112, E911–E920. 10.1073/pnas.1414592112 25605935 PMC4345613

[B32] Finkel O. M. Castrillo G. Paredes S. H. González I. S. Dangl J. L. (2017). Understanding and exploiting plant beneficial microbes. Curr. Opin. Plant Biol. 38, 155–163. 10.1016/j.pbi.2017.04.018 28622659 PMC5561662

[B33] Fischer G. Jena V. Pati B. R. Chandra A. K. (1993). Diazotrophic bacterial population and other associated organisms on the phyllosphere of some crop plants. *Zentralbl*. Mikrobiol. 148, 392–402. 10.1016/S0232-4393(11)80304-5

[B34] Fuentes-Ramirez L. E. Caballero-Mellado J. (2005). Bacterial biofertilizers, in PGPR: Biocontrol and Biofertilization, ed Siddiqui Z. A. (Dordrecht: Springer), 143–172.

[B35] Gnat S. Małek W. Olenska E. Wdowiak-Wróbel S. Kalita M. Łotocka B.. (2015). Phylogeny of symbiotic genes and the symbiotic properties of rhizobia specific to *Astragalus glycyphyllos* L. PLoS ONE 10:e0141504. 10.1371/journal.pone.0141504 26496493 PMC4619719

[B36] Goodman A. L. Kallstrom G. Faith J. J. Reyes A. Moore A. Dantas G.. (2011). Extensive personal human gut microbiota culture collections characterized and manipulated in gnotobiotic mice. Proc. Natl. Acad. Sci. U.S.A. 108, 6252–6257. 10.1073/pnas.1102938108 21436049 PMC3076821

[B37] Grayston S. J. Wang S. Campbell C. D. Edwards A. C. (1998). Selective influence of plant species on microbial diversity in the rhizosphere. Soil Biol. Biochem. 30, 369–378. 10.1016/S0038-0717(97)00124-7

[B38] Gupta R. Mukerji K. G. (2002). Root exudate — biology, in Techniques in Mycorrhizal Studies, eds Mukerji K. G. Manoharachary C. Chamola B. P. (Dordrecht: Springer), 103–131.

[B39] Hadacek F. Kraus G. F. (2002). Plant root carbohydrates affect growth behaviour of endophytic microfungi. FEMS Microbiol. Ecol. 41, 161–170. 10.1111/j.1574-6941.2002.tb00977.x 19709250

[B40] Hoagland D. R. Arnon D. I. (1950). The water-culture method for growing plants without soil. Calif. Agric. Exp. Stn. Circ. 347, 1–32.

[B41] Kielak A. M. Cipriano M. A. Kuramae E. E. (2016). *Acidobacteria* strains from subdivision 1 act as plant growth-promoting bacteria. Arch. Microbiol. 198, 987–993. 10.1007/s00203-016-1260-2 27339258 PMC5080364

[B42] Kim M. Day D. F. (2011). Composition of sugar cane, energy cane, and sweet sorghum suitable for ethanol production at Louisiana sugar mills. J. Ind. Microbiol. Biotechnol. 38, 803–807. 10.1007/s10295-010-0812-8 20803247

[B43] Lebeis S. L. Paredes S. H. Lundberg D. S. Breakfield N. Gehring J. McDonald M.. (2015). Salicylic acid modulates colonization of the root microbiome by specific bacterial taxa. Science 349, 860–864. 10.1126/science.aaa8764 26184915

[B44] Lebeis S. L. Rott M. Dangl J. L. Schulze-Lefert P. (2012). Culturing a plant microbiome community at the cross-Rhodes. New Phytol. 196, 341–344. 10.1111/j.1469-8137.2012.04336.x 22978611

[B45] Li M. Copeland A. Han J. (2011). DUK – A Fast and Efficient Kmer Matching Tool. Available online at: https://publications.lbl.gov/islandora/object/ir%3A155200/datastream/PDF/download/citation.pdf (Accessed March 17, 2017).

[B46] Loiret F. G. Ortega E. Kleiner D. Ortega-Rodés P. Rodés R. Dong Z. (2004). A putative new endophytic nitrogen-fixing bacterium *Pantoea* sp. from sugarcane. J. Appl. Microbiol. 97, 504–511. 10.1111/j.1365-2672.2004.02329.x 15281930

[B47] Lundberg D. S. Lebeis S. L. Paredes S. H. Yourstone S. Gehring J. Malfatti S.. (2012). Defining the core *Arabidopsis thaliana* root microbiome. Nature 488, 86–90. 10.1038/nature11237 22859206 PMC4074413

[B48] Luvizotto D. M. Marcon J. Andreote F. D. Dini-Andreote F. Neves A. A. C. Araújo W. L. (2010). Genetic diversity and plant-growth related features of *Burkholderia* spp. from sugarcane roots. World J. Microbiol. Biotechnol. 26, 1829–1836. 10.1007/s11274-010-0364-0

[B49] Magnani G. S. Didonet C. M. Cruz L. M. Picheth C. F. Pedrosa F. O. Souza E. M. (2010). Diversity of endophytic bacteria in Brazilian sugarcane. Genet. Mol. Res. 9, 250–258. 10.4238/vol9-1gmr703 20198580

[B50] Melorose J. Perroy R. Careas S. (2015). Bacteria in Agrobiology: Plant Probiotics. Berlin: Springer-Verlag.

[B51] Niu B. Paulson J. N. Zheng X. Kolter R. (2017). Simplified and representative bacterial community of maize roots. Proc. Natl. Acad. Sci. U.S.A. 114, E2450–E2459. 10.1073/pnas.1616148114 28275097 PMC5373366

[B52] Olivares F. L. Baldani V. L. D. Reis V. M. Baldani J. I. Döbereiner J. (1996). Occurrence of the endophytic diazotrophs *Herbaspirillum* spp. in roots, stems, and leaves, predominantly of Gramineae. Biol. Fertil. Soils 21, 197–200. 10.1007/BF00335935

[B53] Panke-Buisse K. Poole A. C. Goodrich J. K. Ley R. E. Kao-Kniffin J. (2015). Selection on soil microbiomes reveals reproducible impacts on plant function. ISME J. 9, 980–989. 10.1038/ismej.2014.196 25350154 PMC4817706

[B54] Paungfoo-Lonhienne C. Lonhienne T. G. A. Yeoh Y. K. Webb R. I. Lakshmanan P. Chan C. X.. (2014). A new species of *Burkholderia* isolated from sugarcane roots promotes plant growth. Microb. Biotechnol. 7, 142–154. 10.1111/1751-7915.12105 24350979 PMC3937718

[B55] Perin L. Martínez-Aguilar L. Castro-González R. Estrada-De Los Santos P. Cabellos-Avelar T. Guedes H. V.. (2006). Diazotrophic *Burkholderia* species associated with field-grown maize and sugarcane. Appl. Environ. Microbiol. 72, 3103–3110. 10.1128/AEM.72.5.3103-3110.2006 16672447 PMC1472400

[B56] Pimentel J. P. Olivares F. Pitard R. M. Urquiaga S. Akiba F. Döbereiner J. (1991). Dinitrogen fixation and infection of grass leaves by *Pseudomonas rubrisubalbicans* and *Herbaspirillum seropedicae*. Plant Soil 137, 61–65. 10.1007/BF02187433

[B57] Pisa G. Magnani G. S. Weber H. Souza E. M. Faoro H. Monteiro R. A.. (2011). Diversity of 16S rRNA genes from bacteria of sugarcane rhizosphere soil. Braz. J. Med. Biol. Res. 44, 1215–1221. 10.1590/S0100-879X2011007500148 22042267

[B58] Price M. N. Dehal P. S. Arkin A. P. (2009). FastTree: computing large minimum evolution trees with profiles instead of a distance matrix. Mol. Biol. Evol. 26, 1641–1650. 10.1093/molbev/msp077 19377059 PMC2693737

[B59] Qin S. Zhang Y. J. Yuan B. Xu P. Y. Xing K. Wang J. (2014). Isolation of ACC deaminase-producing habitat-adapted symbiotic bacteria associated with halophyte *Limonium sinense* (Girard) Kuntze and evaluating their plant growth-promoting activity under salt stress. Plant Soil 374, 753–766. 10.1007/s11104-013-1918-3

[B60] Rodriguez R. J. Henson J. Van Volkenburgh E. Hoy M. Wright L. Beckwith F.. (2008). Stress tolerance in plants via habitat-adapted symbiosis. ISME J. 2, 404–416. 10.1038/ismej.2007.106 18256707

[B61] Rokhbakhsh-Zamin F. Sachdev D. Pour N.-K. Engineer A. Pardesi K. R. Zinjarde S.. (2011). Characterization of plant-growth-promoting traits of *Acinetobacter* species isolated from rhizosphere of *Pennisetum glaucum*. J. Microbiol. Biotechnol. 21, 556–566. 10.4014/jmb.1012.12006 21715961

[B62] Schink B. (2002). Synergistic interactions in the microbial world. Antonie Van Leeuwenhoek 81, 257–261. 10.1023/A:1020579004534 12448724

[B63] Schloss P. D. Handelsman J. (2005). Metagenomics for studying unculturable microorganisms: cutting the Gordian knot. Genome Biol. 6:229. 10.1186/gb-2005-6-8-229 16086859 PMC1273625

[B64] Sievers F. Wilm A. Dineen D. Gibson T. J. Karplus K. Li W.. (2011). Fast, scalable generation of high-quality protein multiple sequence alignments using Clustal Omega. Mol. Syst. Biol. 7:539. 10.1038/msb.2011.75 21988835 PMC3261699

[B65] Singh G. Mukerji K. G. (2006). Root exudates as determinant of rhizospheric microbial biodiversity, in Microbial Activity in the Rhizosphere, eds Mukerji K. G. Manoharachary C. Singh J. (Berlin; Heidelberg: Springer), 39–53.

[B66] Stewart E. J. (2012). Growing unculturable bacteria. J. Bacteriol. 194, 4151–4160. 10.1128/JB.00345-12 22661685 PMC3416243

[B67] Taghavi S. Wu X. Ouyang L. Zhang Y. B. Stadler A. McCorkle S.. (2015). Transcriptional responses to sucrose mimic the plant-associated life style of the plant growth promoting endophyte *Enterobacter* sp. 638. PLoS ONE 10:e0115455. 10.1371/journal.pone.0115455 25607953 PMC4301647

[B68] Timm C. M. Pelletier D. A. Jawdy S. S. Gunter L. E. Henning J. A. Engle N.. (2016). Two poplar-associated bacterial isolates induce additive favorable responses in a constructed plant-microbiome system. Front. Plant Sci. 7:497. 10.3389/fpls.2016.00497 27200001 PMC4845692

[B69] Turner T. R. James E. K. Poole P. S. (2013). The plant microbiome. Genome Biol. 14, 209–219. 10.1186/gb-2013-14-6-209 23805896 PMC3706808

[B70] Uren N. (2007). Types, amounts, and possible functions of compounds released into the rhizosphere by soil-grown plants, in The Rhizosphere: Biochemistry and Organic Substances at the Soil-Plant Interface, eds Pinton R. Varanini Z. Nannipieri P. (New York, NY: CRC Press), 1–21.

[B71] Vartoukian S. R. Palmer R. M. Wade W. G. (2010). Strategies for culture of “unculturable” bacteria. FEMS Microbiol. Lett. 309, 1–7. 10.1111/j.1574-6968.2010.02000.x 20487025

[B72] Yoshida M. Ishii S. Fujii D. Otsuka S. Senoo K. (2012). Identification of active denitrifiers in rice paddy soil by DNA- and RNA-based analyses. Microbes Environ. 27, 456–461. 10.1264/jsme2.ME12076 22972387 PMC4103554

[B73] Yuan C. L. Mou C. X. Wu W. L. Guo Y. B. (2011). Effect of different fertilization treatments on indole-3-acetic acid producing bacteria in soil. J. Soils Sediments 11, 322–329. 10.1007/s11368-010-0315-2

[B74] Zarraonaindia I. Owens S. Weisenhorn P. West K. Hampton-Marcell J. Lax S.. (2015). The soil microbiome influences grapevine-associated microbiota. MBio 6:e02527-14. 10.1128/mBio.02527-14 25805735 PMC4453523

[B75] Zengler K. Toledo G. Rappe M. Elkins J. Mathur E. J. Short J. M.. (2002). Cultivating the uncultured. Proc. Natl. Acad. Sci. U.S.A. 99, 15681–15686. 10.1073/pnas.252630999 12438682 PMC137776

