# Supplementary material for: A Community-Based Culture Collection for Targeting Novel Plant Growth-Promoting Bacteria from the Sugarcane Microbiome
Source: Front Plant Sci. 2018 Jan 4;8:2191. doi: 10.3389/fpls.2017.02191 (PMC5759035; doi:10.3389/fpls.2017.02191)
Supplement: Supplementary file 1 [file Table1.pdf]

SUPPLEMENTARY TABLE S1 | Content analysis of the sugarcane juice. Content of glucose, fructose, and sucrose, and physical analyses of the sugarcane juice used as a supplement in culture media, obtained by HPLC analysis.

**Sugar content (g l^{-1})**			**Physical analyses**		
Glucose	Fructose	Sucrose	pH	°Brix	Density (g ml^{-1})
83.78	72.76	545.94	5.17	63.76	1.2612

doi: 10.3389/fpls.2017.02191
