# Supplementary material for: A Community-Based Culture Collection for Targeting Novel Plant Growth-Promoting Bacteria from the Sugarcane Microbiome
Source: Front Plant Sci. 2018 Jan 4;8:2191. doi: 10.3389/fpls.2017.02191 (PMC5759035; doi:10.3389/fpls.2017.02191)
Supplement: Supplementary file 2 [file Table2.pdf]

SUPPLEMENTARY TABLE S2 | Primers and barcode sequences used for library preparation in the multiplex strategy. Primers used are shown for the first- **(A)** and second-step PCR **(B)**.

(A) Plate tagging (first PCR)

Forward primers

Primer_ID	Nextera transposase sequence	MT-FS	Linker	Forward rRNA primer	Complete sequence (5' to 3')
008f_MT	TCGTCGGCAGCGTCAGATGTGTATAAGAGACAG	NNNNNNNNN	AC	AGAGTTTGATCMTGGC	TCGTCGGCAGCGTCAGATGTGTATAAGAGACAGNNNNNNNNNACAGAGTTTGATCMTGGC

Reverse primers (plate tagging)

Primer_ID	Nextera transposase sequence	MT-FS	Barcodes (plate tagging)	Linker	Reverse rRNA primer	Complete sequence (5' to 3')
1492r_MT_bc1	GTCTCGTGGGCTCGGAGATGTGTATAAGAGACAG	NNNN	TTACCGACG	CG	TACCTTGTTACGACTT	GTCTCGTGGGCTCGGAGATGTGTATAAGAGACAGNNNNTTACCGACGCGTACCTTGTTACGACTT
1492r_MT_bc2	GTCTCGTGGGCTCGGAGATGTGTATAAGAGACAG	NNNN	ATTGGACAC	CG	TACCTTGTTACGACTT	GTCTCGTGGGCTCGGAGATGTGTATAAGAGACAGNNNNATTGGACACCGTACCTTGTTACGACTT
1492r_MT_bc3	GTCTCGTGGGCTCGGAGATGTGTATAAGAGACAG	NNNN	TCGCATGGA	CG	TACCTTGTTACGACTT	GTCTCGTGGGCTCGGAGATGTGTATAAGAGACAGNNNNTCGCATGGACGTACCTTGTTACGACTT
1492r_MT_bc4	GTCTCGTGGGCTCGGAGATGTGTATAAGAGACAG	NNNN	AGCGAACCT	CG	TACCTTGTTACGACTT	GTCTCGTGGGCTCGGAGATGTGTATAAGAGACAGNNNNAGCGAACCTCGTACCTTGTTACGACTT
1492r_MT_bc5	GTCTCGTGGGCTCGGAGATGTGTATAAGAGACAG	NNNN	AGCTTCGAC	CG	TACCTTGTTACGACTT	GTCTCGTGGGCTCGGAGATGTGTATAAGAGACAGNNNNAGCTTCGACCGTACCTTGTTACGACTT
1492r_MT_bc6	GTCTCGTGGGCTCGGAGATGTGTATAAGAGACAG	NNNN	GTCAGCCGT	CG	TACCTTGTTACGACTT	GTCTCGTGGGCTCGGAGATGTGTATAAGAGACAGNNNNGTCAGCCGTCGTACCTTGTTACGACTT
1492r_MT_bc7	GTCTCGTGGGCTCGGAGATGTGTATAAGAGACAG	NNNN	TCCAGATAG	CG	TACCTTGTTACGACTT	GTCTCGTGGGCTCGGAGATGTGTATAAGAGACAGNNNNTCCAGATAGCGTACCTTGTTACGACTT
1492r_MT_bc8	GTCTCGTGGGCTCGGAGATGTGTATAAGAGACAG	NNNN	GAGAGTCCA	CG	TACCTTGTTACGACTT	GTCTCGTGGGCTCGGAGATGTGTATAAGAGACAGNNNNGAGAGTCCACGTACCTTGTTACGACTT
1492r_MT_bc9	GTCTCGTGGGCTCGGAGATGTGTATAAGAGACAG	NNNN	GCTCACAAT	CG	TACCTTGTTACGACTT	GTCTCGTGGGCTCGGAGATGTGTATAAGAGACAGNNNNGCTCACAATCGTACCTTGTTACGACTT
1492r_MT_bc10	GTCTCGTGGGCTCGGAGATGTGTATAAGAGACAG	NNNN	TTGACGACA	CG	TACCTTGTTACGACTT	GTCTCGTGGGCTCGGAGATGTGTATAAGAGACAGNNNNTTGACGACACGTACCTTGTTACGACTT
1492r_MT_bc11	GTCTCGTGGGCTCGGAGATGTGTATAAGAGACAG	NNNN	CTTAGAACG	CG	TACCTTGTTACGACTT	GTCTCGTGGGCTCGGAGATGTGTATAAGAGACAGNNNNCTTAGAACGCGTACCTTGTTACGACTT
1492r_MT_bc12	GTCTCGTGGGCTCGGAGATGTGTATAAGAGACAG	NNNN	CGGTTCACA	CG	TACCTTGTTACGACTT	GTCTCGTGGGCTCGGAGATGTGTATAAGAGACAGNNNNCGGTTCACACGTACCTTGTTACGACTT
1492r_MT_bc13	GTCTCGTGGGCTCGGAGATGTGTATAAGAGACAG	NNNN	CGATAGGCC	CG	TACCTTGTTACGACTT	GTCTCGTGGGCTCGGAGATGTGTATAAGAGACAGNNNNCGATAGGCCCGTACCTTGTTACGACTT
1492r_MT_bc14	GTCTCGTGGGCTCGGAGATGTGTATAAGAGACAG	NNNN	GCTATATCC	CG	TACCTTGTTACGACTT	GTCTCGTGGGCTCGGAGATGTGTATAAGAGACAGNNNNGCTATATCCCGTACCTTGTTACGACTT
1492r_MT_bc15	GTCTCGTGGGCTCGGAGATGTGTATAAGAGACAG	NNNN	GTCTTCAGC	CG	TACCTTGTTACGACTT	GTCTCGTGGGCTCGGAGATGTGTATAAGAGACAGNNNNGTCTTCAGCCGTACCTTGTTACGACTT
1492r_MT_bc16	GTCTCGTGGGCTCGGAGATGTGTATAAGAGACAG	NNNN	TAGACACCG	CG	TACCTTGTTACGACTT	GTCTCGTGGGCTCGGAGATGTGTATAAGAGACAGNNNNTAGACACCGCGTACCTTGTTACGACTT
1492r_MT_bc17	GTCTCGTGGGCTCGGAGATGTGTATAAGAGACAG	NNNN	TCAGCTGAC	CG	TACCTTGTTACGACTT	GTCTCGTGGGCTCGGAGATGTGTATAAGAGACAGNNNNTCAGCTGACCGTACCTTGTTACGACTT
1492r_MT_bc18	GTCTCGTGGGCTCGGAGATGTGTATAAGAGACAG	NNNN	TAAGTCGGC	CG	TACCTTGTTACGACTT	GTCTCGTGGGCTCGGAGATGTGTATAAGAGACAGNNNNTAAGTCGGCCGTACCTTGTTACGACTT
1492r_MT_bc19	GTCTCGTGGGCTCGGAGATGTGTATAAGAGACAG	NNNN	GCTCCTTAG	CG	TACCTTGTTACGACTT	GTCTCGTGGGCTCGGAGATGTGTATAAGAGACAGNNNNGCTCCTTAGCGTACCTTGTTACGACTT
1492r_MT_bc20	GTCTCGTGGGCTCGGAGATGTGTATAAGAGACAG	NNNN	ATGGCCTGA	CG	TACCTTGTTACGACTT	GTCTCGTGGGCTCGGAGATGTGTATAAGAGACAGNNNNATGGCCTGACGTACCTTGTTACGACTT
1492r_MT_bc21	GTCTCGTGGGCTCGGAGATGTGTATAAGAGACAG	NNNN	TTGCAAGTA	CG	TACCTTGTTACGACTT	GTCTCGTGGGCTCGGAGATGTGTATAAGAGACAGNNNNTTGCAAGTACGTACCTTGTTACGACTT
1492r_MT_bc22	GTCTCGTGGGCTCGGAGATGTGTATAAGAGACAG	NNNN	CCTAGTAAG	CG	TACCTTGTTACGACTT	GTCTCGTGGGCTCGGAGATGTGTATAAGAGACAGNNNNCCTAGTAAGCGTACCTTGTTACGACTT
1492r_MT_bc23	GTCTCGTGGGCTCGGAGATGTGTATAAGAGACAG	NNNN	CTAGGATCA	CG	TACCTTGTTACGACTT	GTCTCGTGGGCTCGGAGATGTGTATAAGAGACAGNNNNCTAGGATCACGTACCTTGTTACGACTT
1492r_MT_bc24	GTCTCGTGGGCTCGGAGATGTGTATAAGAGACAG	NNNN	TATGAACGT	CG	TACCTTGTTACGACTT	GTCTCGTGGGCTCGGAGATGTGTATAAGAGACAGNNNNTATGAACGTCGTACCTTGTTACGACTT
1492r_MT_bc25	GTCTCGTGGGCTCGGAGATGTGTATAAGAGACAG	NNNN	CTTGTGCGA	CG	TACCTTGTTACGACTT	GTCTCGTGGGCTCGGAGATGTGTATAAGAGACAGNNNNCTTGTGCGACGTACCTTGTTACGACTT
1492r_MT_bc26	GTCTCGTGGGCTCGGAGATGTGTATAAGAGACAG	NNNN	CACGATGGT	CG	TACCTTGTTACGACTT	GTCTCGTGGGCTCGGAGATGTGTATAAGAGACAGNNNNCACGATGGTCGTACCTTGTTACGACTT
1492r_MT_bc27	GTCTCGTGGGCTCGGAGATGTGTATAAGAGACAG	NNNN	ACGTGCCTT	CG	TACCTTGTTACGACTT	GTCTCGTGGGCTCGGAGATGTGTATAAGAGACAGNNNNACGTGCCTTCGTACCTTGTTACGACTT
1492r_MT_bc28	GTCTCGTGGGCTCGGAGATGTGTATAAGAGACAG	NNNN	TGAACTAGC	CG	TACCTTGTTACGACTT	GTCTCGTGGGCTCGGAGATGTGTATAAGAGACAGNNNNTGAACTAGCCGTACCTTGTTACGACTT
1492r_MT_bc29	GTCTCGTGGGCTCGGAGATGTGTATAAGAGACAG	NNNN	TATTCAGCG	CG	TACCTTGTTACGACTT	GTCTCGTGGGCTCGGAGATGTGTATAAGAGACAGNNNNTATTCAGCGCGTACCTTGTTACGACTT
1492r_MT_bc30	GTCTCGTGGGCTCGGAGATGTGTATAAGAGACAG	NNNN	TAATCGGTG	CG	TACCTTGTTACGACTT	GTCTCGTGGGCTCGGAGATGTGTATAAGAGACAGNNNNTAATCGGTGCGTACCTTGTTACGACTT
1492r_MT_bc31	GTCTCGTGGGCTCGGAGATGTGTATAAGAGACAG	NNNN	GCGTCCATG	CG	TACCTTGTTACGACTT	GTCTCGTGGGCTCGGAGATGTGTATAAGAGACAGNNNNGCGTCCATGCGTACCTTGTTACGACTT
1492r_MT_bc32	GTCTCGTGGGCTCGGAGATGTGTATAAGAGACAG	NNNN	CGTAAGATG	CG	TACCTTGTTACGACTT	GTCTCGTGGGCTCGGAGATGTGTATAAGAGACAGNNNNCGTAAGATGCGTACCTTGTTACGACTT
1492r_MT_bc33	GTCTCGTGGGCTCGGAGATGTGTATAAGAGACAG	NNNN	CTGTTACAG	CG	TACCTTGTTACGACTT	GTCTCGTGGGCTCGGAGATGTGTATAAGAGACAGNNNNCTGTTACAGCGTACCTTGTTACGACTT
1492r_MT_bc34	GTCTCGTGGGCTCGGAGATGTGTATAAGAGACAG	NNNN	ACGATCATC	CG	TACCTTGTTACGACTT	GTCTCGTGGGCTCGGAGATGTGTATAAGAGACAGNNNNACGATCATCCGTACCTTGTTACGACTT
1492r_MT_bc35	GTCTCGTGGGCTCGGAGATGTGTATAAGAGACAG	NNNN	GTAACGGCT	CG	TACCTTGTTACGACTT	GTCTCGTGGGCTCGGAGATGTGTATAAGAGACAGNNNNGTAACGGCTCGTACCTTGTTACGACTT
1492r_MT_bc36	GTCTCGTGGGCTCGGAGATGTGTATAAGAGACAG	NNNN	CCATGCTTA	CG	TACCTTGTTACGACTT	GTCTCGTGGGCTCGGAGATGTGTATAAGAGACAGNNNNCCATGCTTACGTACCTTGTTACGACTT
1492r_MT_bc37	GTCTCGTGGGCTCGGAGATGTGTATAAGAGACAG	NNNN	GTACGCACA	CG	TACCTTGTTACGACTT	GTCTCGTGGGCTCGGAGATGTGTATAAGAGACAGNNNNGTACGCACACGTACCTTGTTACGACTT
1492r_MT_bc38	GTCTCGTGGGCTCGGAGATGTGTATAAGAGACAG	NNNN	TTAGAGCCA	CG	TACCTTGTTACGACTT	GTCTCGTGGGCTCGGAGATGTGTATAAGAGACAGNNNNTTAGAGCCACGTACCTTGTTACGACTT
1492r_MT_bc39	GTCTCGTGGGCTCGGAGATGTGTATAAGAGACAG	NNNN	ATAAGGTCG	CG	TACCTTGTTACGACTT	GTCTCGTGGGCTCGGAGATGTGTATAAGAGACAGNNNNATAAGGTCGCGTACCTTGTTACGACTT
1492r_MT_bc40	GTCTCGTGGGCTCGGAGATGTGTATAAGAGACAG	NNNN	AGTGGCACT	CG	TACCTTGTTACGACTT	GTCTCGTGGGCTCGGAGATGTGTATAAGAGACAGNNNNAGTGGCACTCGTACCTTGTTACGACTT
1492r_MT_bc41	GTCTCGTGGGCTCGGAGATGTGTATAAGAGACAG	NNNN	CCAGAAGTG	CG	TACCTTGTTACGACTT	GTCTCGTGGGCTCGGAGATGTGTATAAGAGACAGNNNNCCAGAAGTGCGTACCTTGTTACGACTT
1492r_MT_bc42	GTCTCGTGGGCTCGGAGATGTGTATAAGAGACAG	NNNN	CTACTAGCG	CG	TACCTTGTTACGACTT	GTCTCGTGGGCTCGGAGATGTGTATAAGAGACAGNNNNCTACTAGCGCGTACCTTGTTACGACTT
1492r_MT_bc43	GTCTCGTGGGCTCGGAGATGTGTATAAGAGACAG	NNNN	TAGCGTTCC	CG	TACCTTGTTACGACTT	GTCTCGTGGGCTCGGAGATGTGTATAAGAGACAGNNNNTAGCGTTCCCGTACCTTGTTACGACTT
1492r_MT_bc44	GTCTCGTGGGCTCGGAGATGTGTATAAGAGACAG	NNNN	GTGAGTCAT	CG	TACCTTGTTACGACTT	GTCTCGTGGGCTCGGAGATGTGTATAAGAGACAGNNNNGTGAGTCATCGTACCTTGTTACGACTT
1492r_MT_bc45	GTCTCGTGGGCTCGGAGATGTGTATAAGAGACAG	NNNN	TGGTCCTAC	CG	TACCTTGTTACGACTT	GTCTCGTGGGCTCGGAGATGTGTATAAGAGACAGNNNNTGGTCCTACCGTACCTTGTTACGACTT
1492r_MT_bc46	GTCTCGTGGGCTCGGAGATGTGTATAAGAGACAG	NNNN	TACGCGTAC	CG	TACCTTGTTACGACTT	GTCTCGTGGGCTCGGAGATGTGTATAAGAGACAGNNNNTACGCGTACCGTACCTTGTTACGACTT
1492r_MT_bc47	GTCTCGTGGGCTCGGAGATGTGTATAAGAGACAG	NNNN	GAGCCATCT	CG	TACCTTGTTACGACTT	GTCTCGTGGGCTCGGAGATGTGTATAAGAGACAGNNNNGAGCCATCTCGTACCTTGTTACGACTT
1492r_MT_bc48	GTCTCGTGGGCTCGGAGATGTGTATAAGAGACAG	NNNN	CGTCCGTAT	CG	TACCTTGTTACGACTT	GTCTCGTGGGCTCGGAGATGTGTATAAGAGACAGNNNNCGTCCGTATCGTACCTTGTTACGACTT
1492r_MT_bc49	GTCTCGTGGGCTCGGAGATGTGTATAAGAGACAG	NNNN	GATACGTTC	CG	TACCTTGTTACGACTT	GTCTCGTGGGCTCGGAGATGTGTATAAGAGACAGNNNNGATACGTTCCGTACCTTGTTACGACTT
1492r_MT_bc50	GTCTCGTGGGCTCGGAGATGTGTATAAGAGACAG	NNNN	CAGCTGGTT	CG	TACCTTGTTACGACTT	GTCTCGTGGGCTCGGAGATGTGTATAAGAGACAGNNNNCAGCTGGTTCGTACCTTGTTACGACTT
1492r_MT_bc51	GTCTCGTGGGCTCGGAGATGTGTATAAGAGACAG	NNNN	TTAAGCGCC	CG	TACCTTGTTACGACTT	GTCTCGTGGGCTCGGAGATGTGTATAAGAGACAGNNNNTTAAGCGCCCGTACCTTGTTACGACTT
1492r_MT_bc52	GTCTCGTGGGCTCGGAGATGTGTATAAGAGACAG	NNNN	CCTGCGAAG	CG	TACCTTGTTACGACTT	GTCTCGTGGGCTCGGAGATGTGTATAAGAGACAGNNNNCCTGCGAAGCGTACCTTGTTACGACTT
1492r_MT_bc53	GTCTCGTGGGCTCGGAGATGTGTATAAGAGACAG	NNNN	TTGTAGCCG	CG	TACCTTGTTACGACTT	GTCTCGTGGGCTCGGAGATGTGTATAAGAGACAGNNNNTTGTAGCCGCGTACCTTGTTACGACTT
1492r_MT_bc54	GTCTCGTGGGCTCGGAGATGTGTATAAGAGACAG	NNNN	TCTGTAGAG	CG	TACCTTGTTACGACTT	GTCTCGTGGGCTCGGAGATGTGTATAAGAGACAGNNNNTCTGTAGAGCGTACCTTGTTACGACTT
1492r_MT_bc55	GTCTCGTGGGCTCGGAGATGTGTATAAGAGACAG	NNNN	CTATTAAGC	CG	TACCTTGTTACGACTT	GTCTCGTGGGCTCGGAGATGTGTATAAGAGACAGNNNNCTATTAAGCCGTACCTTGTTACGACTT
1492r_MT_bc56	GTCTCGTGGGCTCGGAGATGTGTATAAGAGACAG	NNNN	CTCTGAGGT	CG	TACCTTGTTACGACTT	GTCTCGTGGGCTCGGAGATGTGTATAAGAGACAGNNNNCTCTGAGGTCGTACCTTGTTACGACTT

(B) Well tagging (second PCR)

Forward primers (row tagging)

Primer_ID	Forward Illumina adapter	Barcode (row tagging)	Nextera transposase sequence	Complete sequence (5' to 3')
S501	AATGATACGGCGACCACCGAGATCTACAC	TAGATCGC	TCGTCGGCAGCGTC	AATGATACGGCGACCACCGAGATCTACACTAGATCGCTCGTCGGCAGCGTC
S502	AATGATACGGCGACCACCGAGATCTACAC	CTCTCTAT	TCGTCGGCAGCGTC	AATGATACGGCGACCACCGAGATCTACACCTCTCTATTCGTCGGCAGCGTC
S503	AATGATACGGCGACCACCGAGATCTACAC	TATCCTCT	TCGTCGGCAGCGTC	AATGATACGGCGACCACCGAGATCTACACTATCCTCTTCGTCGGCAGCGTC
S504	AATGATACGGCGACCACCGAGATCTACAC	AGAGTAGA	TCGTCGGCAGCGTC	AATGATACGGCGACCACCGAGATCTACACAGAGTAGATCGTCGGCAGCGTC
S505	AATGATACGGCGACCACCGAGATCTACAC	GTAAGGAG	TCGTCGGCAGCGTC	AATGATACGGCGACCACCGAGATCTACACGTAAGGAGTCGTCGGCAGCGTC
S506	AATGATACGGCGACCACCGAGATCTACAC	ACTGCATA	TCGTCGGCAGCGTC	AATGATACGGCGACCACCGAGATCTACACACTGCATATCGTCGGCAGCGTC
S507	AATGATACGGCGACCACCGAGATCTACAC	AAGGAGTA	TCGTCGGCAGCGTC	AATGATACGGCGACCACCGAGATCTACACAAGGAGTATCGTCGGCAGCGTC
S508	AATGATACGGCGACCACCGAGATCTACAC	CTAAGCCT	TCGTCGGCAGCGTC	AATGATACGGCGACCACCGAGATCTACACCTAAGCCTTCGTCGGCAGCGTC

Reverse primers (column tagging)

Primer_ID	Reverse Illumina adapter	Barcode (column tagging)	Nextera transposase sequence	Complete sequence (5' to 3')
N701	CAAGCAGAAGACGGCATACGAGAT	TCGCCTTA	GTCTCGTGGGCTCGG	CAAGCAGAAGACGGCATACGAGATTCGCCTTAGTCTCGTGGGCTCGG
N702	CAAGCAGAAGACGGCATACGAGAT	CTAGTACG	GTCTCGTGGGCTCGG	CAAGCAGAAGACGGCATACGAGATCTAGTACGGTCTCGTGGGCTCGG
N703	CAAGCAGAAGACGGCATACGAGAT	TTCTGCCT	GTCTCGTGGGCTCGG	CAAGCAGAAGACGGCATACGAGATTTCTGCCTGTCTCGTGGGCTCGG
N704	CAAGCAGAAGACGGCATACGAGAT	GCTCAGGA	GTCTCGTGGGCTCGG	CAAGCAGAAGACGGCATACGAGATGCTCAGGAGTCTCGTGGGCTCGG
N705	CAAGCAGAAGACGGCATACGAGAT	AGGAGTCC	GTCTCGTGGGCTCGG	CAAGCAGAAGACGGCATACGAGATAGGAGTCCGTCTCGTGGGCTCGG
N706	CAAGCAGAAGACGGCATACGAGAT	CATGCCTA	GTCTCGTGGGCTCGG	CAAGCAGAAGACGGCATACGAGATCATGCCTAGTCTCGTGGGCTCGG
N707	CAAGCAGAAGACGGCATACGAGAT	GTAGAGAG	GTCTCGTGGGCTCGG	CAAGCAGAAGACGGCATACGAGATGTAGAGAGGTCTCGTGGGCTCGG
N708	CAAGCAGAAGACGGCATACGAGAT	CCTCTCTG	GTCTCGTGGGCTCGG	CAAGCAGAAGACGGCATACGAGATCCTCTCTGGTCTCGTGGGCTCGG
N709	CAAGCAGAAGACGGCATACGAGAT	AGCGTAGC	GTCTCGTGGGCTCGG	CAAGCAGAAGACGGCATACGAGATAGCGTAGCGTCTCGTGGGCTCGG
N710	CAAGCAGAAGACGGCATACGAGAT	CAGCCTCG	GTCTCGTGGGCTCGG	CAAGCAGAAGACGGCATACGAGATCAGCCTCGGTCTCGTGGGCTCGG
N711	CAAGCAGAAGACGGCATACGAGAT	TGCCTCTT	GTCTCGTGGGCTCGG	CAAGCAGAAGACGGCATACGAGATTGCCTCTTGTCTCGTGGGCTCGG
N712	CAAGCAGAAGACGGCATACGAGAT	TCCTCTAC	GTCTCGTGGGCTCGG	CAAGCAGAAGACGGCATACGAGATTCCTCTACGTCTCGTGGGCTCGG

doi: 10.3389/fpls.2017.02191
