# Supplementary material for: A Community-Based Culture Collection for Targeting Novel Plant Growth-Promoting Bacteria from the Sugarcane Microbiome
Source: Front Plant Sci. 2018 Jan 4;8:2191. doi: 10.3389/fpls.2017.02191 (PMC5759035; doi:10.3389/fpls.2017.02191)
Supplement: Supplementary file 3 [file Table3.pdf]

SUPPLEMENTARY TABLE S3 | Sequence statistics of 16S pooled libraries from multiplex amplicon sequencing for bacterial identification in the CBC. The multiplex amplicon strategy allows to pool libraries from different plates of the culture collection for sequencing in PacBio plataform. Numbers of demultiplexed CCSs, non-chimaeras, chimaeras, 16S sequences, non-16S sequences, reliable sequences and number of 16S rRNA-positive PCR bands are shown per plate and sequenced pools.

Pool name	Plate code	Barcode	Demultiplexed CCSs		Non-chimaeras		Chimaeras		16S sequences		Non-16S sequences		Reliable sequences		Reliable wells		Wells with bands	
			plate	*pool*	*plate*	*pool*	*plate*	*pool*	*plate*	*pool*	*plate*	*pool*	*plate*	*pool*	*plate*	*pool*	*plate*	*pool*
	001.1	36	1,093		969		124		960		9		868		71		72	
	001.2	37	1,712		1,522		190		1,519		3		1,374		79		73	
pool0105	001.3	38	1,718	6,701	1,574	6,005	144	696	1,573	5,920	1	85	1,431	5,339	83	374	76	357
	002.1	39	1,181		1,068		113		1,064		4		960		74		70	
	002.2	40	997		872		125		804		68		706		67		66	
	002.3	41	842		734		108		734		0		713		62		61	
	002.4	42	1,389		1,243		146		1,242		1		1,187		74		68	
pool0610	002.5	43	2,553	8,846	2,346	7,938	207	908	2,342	7,925	4	13	2,293	7,700	82	365	76	350
	002.6	44	2,648		2,401		247		2,400		1		2,345		83		75	
	003.1	45	1,414		1,214		200		1,207		7		1,162		64		70	
	003.2	36	2,783		2,425		358		2,419		6		2,339		75		64	
	004.1	37	2,104		1,974		130		1,974		0		1,909		75		59	
pool1115	005.1	38	3,137	13,272	2,943	12,299	194	973	2,942	12,292	1	7	2,885	12,018	73	348	62	274
	005.2	39	1,015		907		108		907		0		882		47		25	
	005.3	40	4,233		4,050		183		4,050		0		4,003		78		64	
	005.4	16	328		312		16		312		0		304		48		38	
	006.1	17	661		554		107		554		0		541		53		56	
pool1620	006.2	18	591	4,039	527	3,785	64	254	527	3,784	0	1	520	3,714	51	297	44	269
	007.1	19	1,665		1,615		50		1,614		1		1,586		79		50	
	007.2	20	794		777		17		777		0		763		66		81	
	007.3	21	545		498		47		498		0		476		33		55	
	007.4	22	2,008		1,969		39		1,968		1		1,879		73		48	
pool2125	007.5	23	1,064	13,443	1,032	13,243	32	200	1,032	13,240	0	3	998	12,903	36	252	46	259
	007.6	24	569		568		1		567		1		558		20		31	
	007.7	25	9,257		9,176		81		9,175		1		8,992		90		79	
	007.8	26	2		2		0		2		0		2		1		45	
	007.9	27	0		0		0		0		0		0		0		11	
pool2630	008.1	28	0	23	0	23	0	0	0	23	0	0	0	23	0	2	20	136
	008.2	29	0		0		0		0		0		0		0		34	
	008.3	30	21		21		0		21		0		21		1		26	
	008.4	31	420		413		7		411		2		400		28		16	
	008.5	32	925		696		229		696		0		682		34		23	
pool3135	008.6	33	3,279	5,890	3,174	5,480	105	410	3,172	5,476	2	4	3,132	5,357	57	191	38	119
	008.7	34	318		306		12		306		0		286		29		20	
	008.8	35	948		891		57		891		0		857		43		22	
	008.9	36	254		231		23		231		0		225		23		35	
	009.1	37	1,446		1,324		122		1,323		1		1,271		59		72	
pool3640	010.1	38	796	6,203	747	5,676	49	527	744	5,669	3	7	714	5,516	47	234	46	260
	010.2	39	2,237		2,005		232		2,002		3		1,971		49		51	
	010.3	40	1,470		1,369		101		1,369		0		1,335		56		56	
	010.4	41	2,504		2,247		257		2,246		1		2,172		86		85	
pool4144	010.5	42	4,971	12,502	4,554	11,289	417	1,213	4,554	11,272	0	17	4,467	10,919	85	307	77	289
	010.6	43	2,649		2,313		336		2,297		16		2,203		70		70	
	010.7	44	2,378		2,175		203		2,175		0		2,077		66		57	
	013.1	45	4,372		4,164		208		4,160		4		4,092		83		52	
	013.2	46	2,602		2,393		209		2,364		29		2,285		75		46	
pool4550	013.3	47	25	9,145	25	8,660	0	485	25	8,624	0	36	22	8,416	13	271	32	270
	013.4	48	2,096		2,029		67		2,026		3		1,978		76		38	
	013.5	49	49		48		1		48		0		38		23		60	
	013.6	50	1		1		0		1		0		1		1		42	
	014.1	41	402		384		18		384		0		377		20		19	
	016.1	42	1,251		1,191		60		1,191		0		1,168		56		51	
pool5156	016.2	43	2,056	9,564	2,013	9,189	43	375	2,013	9,181	0	8	1,999	9,054	60	301	47	254
	016.3	44	1,912		1,850		62		1,845		5		1,813		59		47	
	016.4	45	1,298		1,183		115		1,180		3		1,161		45		38	
	016.5	46	2,645		2,568		77		2,568		0		2,536		61		52	
	**Total**		89,628	89,628	83,587	83,587	6,041	6,041	83,406	83,406	181	181	80,959	80,959	2,942	2,942	2,837	2,837

doi: 10.3389/fpls.2017.02191
