# Supplementary material for: A Community-Based Culture Collection for Targeting Novel Plant Growth-Promoting Bacteria from the Sugarcane Microbiome
Source: Front Plant Sci. 2018 Jan 4;8:2191. doi: 10.3389/fpls.2017.02191 (PMC5759035; doi:10.3389/fpls.2017.02191)
Supplement: Supplementary file 4 [file Table4.pdf]

SUPPLEMENTARY TABLE S4 | Number of mOTUs from sugarcane organs recovered by the sugarcane CBC. Recovery estimates of mOTUs for roots, stalks (bottom, medium and upper) and leaves were calculated by cross-referencing the mOTUs (based on the culture-independent microbiome profile) with the cOTUs of the sugarcane CBC (based on the culture-dependent profile). End, endophytic; Exo, exophytic.

**Sugarcane organ**	**Core microbiome**		**Non-core microbiome**	
	mOTU	*cOTU*	*mOTU*	*cOTU*
Bulk soil	173	100	87	56
Rhizosphere	128	75	164	99
End. root	133	79	164	100
End. young shoot	104	62	195	111
End. bottom stalk	66	48	208	113
End. medium stalk	56	40	185	109
End. upper stalk	48	33	200	114
End. leaf	31	26	186	102
Exo. bottom stalk	83	58	230	121
Exo. medium stalk	45	33	249	131
Exo. upper stalk	40	29	222	121
Exo. leaf	63	46	207	116

doi: 10.3389/fpls.2017.02191
