# Supplementary material for: A Community-Based Culture Collection for Targeting Novel Plant Growth-Promoting Bacteria from the Sugarcane Microbiome
Source: Front Plant Sci. 2018 Jan 4;8:2191. doi: 10.3389/fpls.2017.02191 (PMC5759035; doi:10.3389/fpls.2017.02191)
Supplement: Supplementary file 5 [file Table5.pdf]

SUPPLEMENTARY TABLE S5 | Taxonomic identification of unique cOTUs in the CBC and number of wells in which cOTUs were found. cOTUs were classified using "utax" in USEARCH considering a minimum confidence score of 0.9.

cOTU	Taxonomy	Number of wells
OTU_1	d:Bacteria,p:Firmicutes,c:Bacilli,o:Bacillales,f:Bacillaceae_1,g:Bacillus	575
OTU_2	d:Bacteria,p:"Proteobacteria",c:Alphaproteobacteria,o:Rhizobiales,f:Rhizobiaceae,g:Rhizobium	449
OTU_14	d:Bacteria,p:"Bacteroidetes",c:Sphingobacteriia,o:"Sphingobacteriales",f:Chitinophagaceae,g:Chitinophaga	291
OTU_15	d:Bacteria,p:"Proteobacteria",c:Gammaproteobacteria,o:"Enterobacteriales",f:Enterobacteriaceae	229
OTU_5	d:Bacteria,p:"Proteobacteria",c:Betaproteobacteria,o:Burkholderiales,f:Burkholderiaceae,g:Burkholderia	206
OTU_6	d:Bacteria,p:"Proteobacteria",c:Gammaproteobacteria,o:Xanthomonadales,f:Xanthomonadaceae	171
OTU_7	d:Bacteria,p:"Proteobacteria",c:Betaproteobacteria,o:Burkholderiales,f:Comamonadaceae	169
OTU_4	d:Bacteria,p:"Proteobacteria",c:Gammaproteobacteria,o:Xanthomonadales,f:Xanthomonadaceae	162
OTU_23	d:Bacteria,p:Firmicutes,c:Bacilli,o:Bacillales,f:Paenibacillaceae_1,g:Brevibacillus	139
OTU_3	d:Bacteria,p:"Proteobacteria",c:Betaproteobacteria,o:Burkholderiales,f:Burkholderiaceae	111
OTU_16	d:Bacteria,p:Firmicutes,c:Bacilli,o:Bacillales,f:Planococcaceae	89
OTU_8	d:Bacteria,p:"Proteobacteria",c:Alphaproteobacteria,o:Rhizobiales,f:Beijerinckiaceae	88
OTU_17	d:Bacteria,p:"Bacteroidetes",c:Sphingobacteriia,o:"Sphingobacteriales",f:Chitinophagaceae,g:Chitinophaga	76
OTU_9	d:Bacteria,p:"Proteobacteria",c:Gammaproteobacteria,o:Xanthomonadales,f:Xanthomonadaceae,g:Lysobacter	74
OTU_234	d:Bacteria,p:"Proteobacteria",c:Gammaproteobacteria,o:Xanthomonadales,f:Xanthomonadaceae	65
OTU_19	d:Bacteria,p:"Bacteroidetes",c:Sphingobacteriia,o:"Sphingobacteriales",f:Sphingobacteriaceae,g:Mucilaginibacter	53
OTU_13	d:Bacteria,p:"Actinobacteria",c:Actinobacteria,o:Actinomycetales,f:Promicromonosporaceae	51
OTU_350	d:Bacteria,p:"Proteobacteria",c:Alphaproteobacteria,o:Rhizobiales	49
OTU_74	d:Bacteria,p:"Proteobacteria",c:Betaproteobacteria,o:Burkholderiales,f:Burkholderiaceae	42
OTU_20	d:Bacteria,p:"Proteobacteria",c:Alphaproteobacteria,o:Rhodospirillales,f:Rhodospirillaceae,g:Inquilinus	41
OTU_25	d:Bacteria,p:"Bacteroidetes",c:Sphingobacteriia,o:"Sphingobacteriales",f:Sphingobacteriaceae,g:Pedobacter	41
OTU_10	d:Bacteria,p:"Proteobacteria",c:Alphaproteobacteria,o:Rhizobiales,f:Rhizobiaceae	39
OTU_42	d:Bacteria,p:"Actinobacteria",c:Actinobacteria,o:Actinomycetales	39
OTU_21	d:Bacteria,p:"Bacteroidetes",c:Flavobacteriia,o:"Flavobacteriales",f:Flavobacteriaceae	37
OTU_22	d:Bacteria,p:"Actinobacteria",c:Actinobacteria,o:Actinomycetales,f:Microbacteriaceae	35
OTU_52	d:Bacteria,p:"Actinobacteria",c:Actinobacteria,o:Actinomycetales,f:Microbacteriaceae,g:Curtobacterium	31
OTU_28	d:Bacteria,p:Firmicutes,c:Bacilli,o:Bacillales,f:Bacillaceae_1,g:Bacillus	30
OTU_157	d:Bacteria,p:"Proteobacteria",c:Gammaproteobacteria,o:Pseudomonadales,f:Moraxellaceae,g:Acinetobacter	30
OTU_270	d:Bacteria,p:"Proteobacteria",c:Gammaproteobacteria,o:"Enterobacteriales",f:Enterobacteriaceae	25
OTU_24	d:Bacteria,p:"Proteobacteria",c:Betaproteobacteria,o:Burkholderiales,f:Oxalobacteraceae	23
OTU_95	d:Bacteria,p:"Actinobacteria",c:Actinobacteria,o:Actinomycetales,f:Microbacteriaceae	23
OTU_41	d:Bacteria,p:"Proteobacteria",c:Betaproteobacteria,o:Burkholderiales,f:Burkholderiaceae	21
OTU_12	d:Bacteria,p:"Proteobacteria",c:Alphaproteobacteria,o:Caulobacterales,f:Caulobacteraceae,g:Asticcacaulis	19
OTU_167	d:Bacteria,p:"Actinobacteria",c:Actinobacteria,o:Actinomycetales,f:Microbacteriaceae,g:Microbacterium	19
OTU_37	d:Bacteria,p:"Proteobacteria",c:Alphaproteobacteria,o:Caulobacterales,f:Caulobacteraceae	17
OTU_135	d:Bacteria,p:"Proteobacteria",c:Betaproteobacteria,o:Burkholderiales,f:Burkholderiaceae	16
OTU_11	d:Bacteria,p:"Actinobacteria",c:Actinobacteria,o:Actinomycetales,f:Micrococcaceae,g:Arthrobacter	15
OTU_18	d:Bacteria,p:"Proteobacteria",c:Alphaproteobacteria,o:Rhizobiales,f:Methylobacteriaceae,g:Methylobacterium	15
OTU_121	d:Bacteria,p:"Proteobacteria",c:Alphaproteobacteria,o:Sphingomonadales,f:Sphingomonadaceae	15
OTU_243	d:Bacteria,p:"Actinobacteria",c:Actinobacteria,o:Actinomycetales,f:Micrococcaceae	15
OTU_267	d:Bacteria,p:Firmicutes,c:Bacilli,o:Bacillales,f:Paenibacillaceae_1,g:Paenibacillus	15
OTU_80	d:Bacteria,p:"Bacteroidetes",c:Sphingobacteriia	14
OTU_81	d:Bacteria,p:"Bacteroidetes",c:Sphingobacteriia,o:"Sphingobacteriales",f:Chitinophagaceae,g:Chitinophaga	14
OTU_51	d:Bacteria,p:"Actinobacteria",c:Actinobacteria,o:Actinomycetales,f:Streptomycetaceae,g:Streptomyces	13
OTU_50	d:Bacteria,p:"Proteobacteria",c:Alphaproteobacteria,o:Rhizobiales	12
OTU_163	d:Bacteria,p:"Proteobacteria",c:Alphaproteobacteria,o:Rhizobiales	12
OTU_27	d:Bacteria,p:"Proteobacteria",c:Gammaproteobacteria,o:Pseudomonadales,f:Pseudomonadaceae,g:Pseudomonas	11
OTU_53	d:Bacteria,p:"Proteobacteria",c:Gammaproteobacteria,o:Xanthomonadales,f:Xanthomonadaceae	11
OTU_59	d:Bacteria,p:"Proteobacteria",c:Betaproteobacteria,o:Burkholderiales,f:Burkholderiaceae	11
OTU_66	d:Bacteria,p:Firmicutes,c:Bacilli,o:Bacillales,f:Bacillaceae_1,g:Bacillus	11

doi: 10.3389/fpls.2017.02191

OTU_162	d:Bacteria,p:"Proteobacteria",c:Alphaproteobacteria,o:Sphingomonadales,f:Sphingomonadaceae	11
OTU_262	d:Bacteria,p:"Proteobacteria",c:Betaproteobacteria,o:Burkholderiales	11
OTU_279	d:Bacteria,p:Firmicutes,c:Bacilli,o:Bacillales,f:Planococcaceae	11
OTU_45	d:Bacteria,p:"Bacteroidetes",c:Cytophagia,o:Cytophagales,f:Cytophagaceae,g:Dyadobacter	10
OTU_102	d:Bacteria,p:"Actinobacteria",c:Actinobacteria,o:Actinomycetales,f:Streptomycetaceae,g:Streptomyces	10
OTU_49	d:Bacteria,p:"Actinobacteria",c:Actinobacteria,o:Actinomycetales,f:Micrococcaceae	9
OTU_65	d:Bacteria,p:"Proteobacteria",c:Alphaproteobacteria,o:Rhizobiales,f:Bradyrhizobiaceae,g:Bosea	9
OTU_117	d:Bacteria,p:"Proteobacteria",c:Alphaproteobacteria	9
OTU_138	d:Bacteria,p:"Proteobacteria",c:Alphaproteobacteria,o:Rhizobiales,f:Rhizobiaceae	9
OTU_166	d:Bacteria,p:Firmicutes,c:Bacilli,o:Bacillales,f:Bacillaceae_1	9
OTU_251	d:Bacteria,p:"Proteobacteria",c:Betaproteobacteria,o:Burkholderiales,f:Alcaligenaceae,g:Advenella	9
OTU_34	d:Bacteria,p:"Bacteroidetes",c:Flavobacteriia,o:"Flavobacteriales",f:Flavobacteriaceae	8
OTU_119	d:Bacteria,p:Firmicutes,c:Bacilli,o:Bacillales,f:Bacillaceae_1,g:Bacillus	8
OTU_120	d:Bacteria,p:"Proteobacteria",c:Gammaproteobacteria	8
OTU_160	d:Bacteria,p:"Actinobacteria",c:Actinobacteria,o:Actinomycetales	8
OTU_180	d:Bacteria,p:"Proteobacteria",c:Gammaproteobacteria,o:"Enterobacteriales",f:Enterobacteriaceae	8
OTU_194	d:Bacteria,p:"Actinobacteria",c:Actinobacteria,o:Solirubrobacterales,f:Patulibacteraceae,g:Patulibacter	8
OTU_213	d:Bacteria,p:"Proteobacteria",c:Gammaproteobacteria	8
OTU_229	d:Bacteria,p:Firmicutes,c:Bacilli,o:Bacillales,f:Paenibacillaceae_1	8
OTU_236	d:Bacteria,p:"Proteobacteria",c:Alphaproteobacteria,o:Rhizobiales,f:Rhizobiaceae	8
OTU_241	d:Bacteria,p:"Actinobacteria",c:Actinobacteria,o:Actinomycetales,f:Microbacteriaceae	8
OTU_382	d:Bacteria,p:"Proteobacteria",c:Betaproteobacteria,o:Burkholderiales,f:Burkholderiaceae,g:Burkholderia	8
OTU_30	d:Bacteria,p:"Proteobacteria",c:Gammaproteobacteria,o:Pseudomonadales,f:Pseudomonadaceae	7
OTU_72	d:Bacteria,p:"Proteobacteria",c:Betaproteobacteria,o:Burkholderiales,f:Burkholderiaceae	7
OTU_97	d:Bacteria,p:"Proteobacteria",c:Alphaproteobacteria,o:Sphingomonadales,f:Sphingomonadaceae	7
OTU_142	d:Bacteria,p:"Proteobacteria",c:Alphaproteobacteria,o:Sphingomonadales	7
OTU_231	d:Bacteria,p:"Proteobacteria",c:Betaproteobacteria,o:Burkholderiales	7
OTU_288	d:Bacteria,p:"Proteobacteria",c:Betaproteobacteria,o:Burkholderiales,f:Burkholderiaceae	7
OTU_317	d:Bacteria,p:"Proteobacteria",c:Alphaproteobacteria,o:Rhizobiales,f:Methylobacteriaceae	7
OTU_394	d:Bacteria,p:"Proteobacteria",c:Betaproteobacteria,o:Burkholderiales,f:Burkholderiaceae	7
OTU_35	d:Bacteria,p:"Bacteroidetes",c:Sphingobacteriia,o:"Sphingobacteriales",f:Sphingobacteriaceae,g:Mucilaginibacter	6
OTU_67	d:Bacteria,p:"Proteobacteria",c:Betaproteobacteria	6
OTU_123	d:Bacteria,p:"Proteobacteria",c:Alphaproteobacteria,o:Sphingomonadales,f:Sphingomonadaceae,g:Sphingomonas	6
OTU_139	d:Bacteria,p:"Proteobacteria",c:Alphaproteobacteria,o:Rhizobiales	6
OTU_248	d:Bacteria,p:"Proteobacteria",c:Alphaproteobacteria,o:Rhizobiales	6
OTU_285	d:Bacteria,p:"Proteobacteria",c:Alphaproteobacteria,o:Caulobacterales,f:Caulobacteraceae,g:Brevundimonas	6
OTU_326	d:Bacteria,p:"Actinobacteria",c:Actinobacteria,o:Actinomycetales,f:Promicromonosporaceae	6
OTU_331	d:Bacteria,p:"Proteobacteria",c:Alphaproteobacteria,o:Rhizobiales,f:Xanthobacteraceae,g:Labrys	6
OTU_369	d:Bacteria,p:"Actinobacteria",c:Actinobacteria,o:Actinomycetales,f:Microbacteriaceae	6
OTU_63	d:Bacteria,p:"Proteobacteria",c:Gammaproteobacteria	5
OTU_82	d:Bacteria,p:"Proteobacteria",c:Gammaproteobacteria	5
OTU_86	d:Bacteria,p:"Proteobacteria",c:Gammaproteobacteria,o:Pseudomonadales,f:Pseudomonadaceae	5
OTU_143	d:Bacteria,p:"Actinobacteria",c:Actinobacteria,o:Actinomycetales,f:Nocardioidaceae	5
OTU_187	d:Bacteria,p:"Proteobacteria",c:Alphaproteobacteria,o:Rhizobiales	5
OTU_376	d:Bacteria,p:"Bacteroidetes",c:Flavobacteriia,o:"Flavobacteriales",f:Flavobacteriaceae	5
OTU_380	d:Bacteria,p:"Bacteroidetes",c:Sphingobacteriia,o:"Sphingobacteriales",f:Sphingobacteriaceae	5
OTU_39	d:Bacteria,p:"Proteobacteria",c:Gammaproteobacteria	4
OTU_48	d:Bacteria,p:"Acidobacteria",c:Acidobacteria_Gp1	4
OTU_118	d:Bacteria,p:"Proteobacteria",c:Alphaproteobacteria,o:Rhizobiales	4
OTU_150	d:Bacteria,p:"Proteobacteria",c:Alphaproteobacteria,o:Caulobacterales,f:Caulobacteraceae	4
OTU_181	d:Bacteria,p:"Proteobacteria",c:Alphaproteobacteria	4
OTU_186	d:Bacteria,p:"Bacteroidetes",c:Flavobacteriia,o:"Flavobacteriales",f:Flavobacteriaceae	4
OTU_215	d:Bacteria,p:Firmicutes,c:Bacilli,o:Bacillales,f:Bacillaceae_1	4
OTU_219	d:Bacteria,p:"Bacteroidetes",c:Sphingobacteriia,o:"Sphingobacteriales",f:Chitinophagaceae,g:Chitinophaga	4
OTU_225	d:Bacteria,p:"Actinobacteria",c:Actinobacteria,o:Actinomycetales	4
OTU_235	d:Bacteria,p:"Proteobacteria",c:Alphaproteobacteria,o:Sphingomonadales	4

doi: 10.3389/fpls.2017.02191

OTU_249	d:Bacteria,p:"Actinobacteria",c:Actinobacteria,o:Actinomycetales,f:Promicromonosporaceae	4
OTU_256	d:Bacteria,p:Firmicutes,c:Bacilli,o:Bacillales,f:Bacillaceae_1,g:Bacillus	4
OTU_278	d:Bacteria,p:"Proteobacteria",c:Betaproteobacteria	4
OTU_294	d:Bacteria,p:Firmicutes,c:Bacilli,o:Bacillales	4
OTU_347	d:Bacteria,p:"Proteobacteria",c:Alphaproteobacteria,o:Rhizobiales	4
OTU_36	d:Bacteria,p:"Proteobacteria",c:Gammaproteobacteria,o:Xanthomonadales	3
OTU_56	d:Bacteria,p:"Bacteroidetes",c:Sphingobacteriia,o:"Sphingobacteriales",f:Chitinophagaceae	3
OTU_61	d:Bacteria,p:"Proteobacteria",c:Betaproteobacteria,o:Burkholderiales,f:Burkholderiaceae	3
OTU_76	d:Bacteria,p:"Proteobacteria",c:Betaproteobacteria	3
OTU_88	d:Bacteria,p:"Bacteroidetes",c:Sphingobacteriia,o:"Sphingobacteriales",f:Sphingobacteriaceae	3
OTU_90	d:Bacteria,p:"Proteobacteria",c:Gammaproteobacteria,o:Xanthomonadales,f:Xanthomonadaceae,g:Dokdonella	3
OTU_92	d:Bacteria,p:"Proteobacteria",c:Betaproteobacteria	3
OTU_110	d:Bacteria,p:"Proteobacteria",c:Betaproteobacteria,o:Burkholderiales,f:Alcaligenaceae	3
OTU_111	d:Bacteria,p:"Bacteroidetes",c:Sphingobacteriia,o:"Sphingobacteriales",f:Chitinophagaceae,g:Chitinophaga	3
OTU_132	d:Bacteria,p:"Proteobacteria",c:Alphaproteobacteria,o:Rhizobiales	3
OTU_136	d:Bacteria,p:"Proteobacteria",c:Gammaproteobacteria,o:Xanthomonadales,f:Xanthomonadaceae	3
OTU_155	d:Bacteria,p:"Proteobacteria",c:Alphaproteobacteria,o:Rhizobiales	3
OTU_174	d:Bacteria,p:"Actinobacteria",c:Actinobacteria,o:Actinomycetales,f:Nocardioidaceae	3
OTU_182	d:Bacteria,p:"Proteobacteria",c:Gammaproteobacteria,o:"Enterobacteriales",f:Enterobacteriaceae	3
OTU_195	d:Bacteria,p:"Proteobacteria",c:Alphaproteobacteria,o:Rhizobiales,f:Rhizobiaceae	3
OTU_200	d:Bacteria,p:"Proteobacteria",c:Betaproteobacteria	3
OTU_202	d:Bacteria,p:"Proteobacteria",c:Alphaproteobacteria,o:Rhizobiales	3
OTU_211	d:Bacteria,p:"Actinobacteria",c:Actinobacteria,o:Actinomycetales,f:Intrasporangiaceae	3
OTU_212	d:Bacteria,p:Firmicutes,c:Bacilli,o:Bacillales,f:Paenibacillaceae_1	3
OTU_259	d:Bacteria,p:"Bacteroidetes",c:Sphingobacteriia,o:"Sphingobacteriales",f:Chitinophagaceae,g:Chitinophaga	3
OTU_277	d:Bacteria,p:"Actinobacteria",c:Actinobacteria,o:Actinomycetales,f:Promicromonosporaceae	3
OTU_286	d:Bacteria,p:Firmicutes,c:Bacilli,o:Bacillales,f:Paenibacillaceae_2	3
OTU_291	d:Bacteria,p:Firmicutes,c:Bacilli,o:Bacillales	3
OTU_303	d:Bacteria,p:Firmicutes,c:Bacilli,o:Bacillales,f:Bacillaceae_1	3
OTU_307	d:Bacteria,p:"Proteobacteria",c:Betaproteobacteria,o:Burkholderiales	3
OTU_318	d:Bacteria,p:"Actinobacteria",c:Actinobacteria,o:Actinomycetales,f:Micrococcaceae	3
OTU_334	d:Bacteria,p:Firmicutes,c:Bacilli,o:Bacillales,f:Paenibacillaceae_1,g:Brevibacillus	3
OTU_335	d:Bacteria,p:"Proteobacteria",c:Gammaproteobacteria,o:"Enterobacteriales",f:Enterobacteriaceae	3
OTU_353	d:Bacteria,p:"Proteobacteria",c:Alphaproteobacteria,o:Sphingomonadales	3
OTU_364	d:Bacteria,p:"Proteobacteria",c:Gammaproteobacteria	3
OTU_367	d:Bacteria,p:"Actinobacteria",c:Actinobacteria,o:Actinomycetales,f:Microbacteriaceae	3
OTU_399	d:Bacteria,p:"Proteobacteria",c:Gammaproteobacteria,o:"Enterobacteriales",f:Enterobacteriaceae	3
OTU_400	d:Bacteria,p:"Proteobacteria",c:Alphaproteobacteria,o:Rhizobiales,f:Rhizobiaceae	3
OTU_40	d:Bacteria,p:"Proteobacteria",c:Alphaproteobacteria	2
OTU_46	d:Bacteria,p:"Proteobacteria",c:Gammaproteobacteria,o:"Enterobacteriales",f:Enterobacteriaceae	2
OTU_62	d:Bacteria,p:"Actinobacteria",c:Actinobacteria,o:Actinomycetales,f:Microbacteriaceae	2
OTU_68	d:Bacteria,p:Firmicutes,c:Bacilli,o:Bacillales,f:Planococcaceae	2
OTU_69	d:Bacteria,p:"Proteobacteria"	2
OTU_73	d:Bacteria,p:"Proteobacteria",c:Alphaproteobacteria,o:Rhizobiales,f:Rhizobiaceae	2
OTU_75	d:Bacteria,p:"Proteobacteria",c:Betaproteobacteria,o:Burkholderiales,f:Burkholderiaceae	2
OTU_85	d:Bacteria,p:"Bacteroidetes",c:Flavobacteriia,o:"Flavobacteriales",f:Flavobacteriaceae,g:Flavobacterium	2
OTU_87	d:Bacteria,p:"Proteobacteria",c:Alphaproteobacteria,o:Rhizobiales,f:Phyllobacteriaceae	2
OTU_89	d:Bacteria,p:"Proteobacteria",c:Betaproteobacteria,o:Burkholderiales,f:Burkholderiaceae	2
OTU_116	d:Bacteria,p:"Proteobacteria",c:Betaproteobacteria,o:Burkholderiales,f:Burkholderiaceae	2
OTU_130	d:Bacteria,p:"Bacteroidetes",c:Sphingobacteriia,o:"Sphingobacteriales",f:Chitinophagaceae	2
OTU_134	d:Bacteria,p:Firmicutes,c:Bacilli,o:Bacillales,f:Bacillaceae_1,g:Bacillus	2
OTU_137	d:Bacteria,p:"Proteobacteria",c:Alphaproteobacteria	2
OTU_151	d:Bacteria,p:"Proteobacteria",c:Alphaproteobacteria,o:Caulobacterales,f:Caulobacteraceae,g:Caulobacter	2
OTU_153	d:Bacteria,p:Firmicutes,c:Bacilli,o:Bacillales,f:Bacillaceae_1,g:Bacillus	2
OTU_159	d:Bacteria,p:Firmicutes,c:Bacilli,o:Bacillales,f:Bacillaceae_1	2
OTU_165	d:Bacteria,p:"Bacteroidetes",c:Sphingobacteriia,o:"Sphingobacteriales",f:Chitinophagaceae,g:Chitinophaga	2

doi: 10.3389/fpls.2017.02191

OTU_170	d:Bacteria,p:"Bacteroidetes",c:Sphingobacteriia,o:"Sphingobacteriales",f:Sphingobacteriaceae	2
OTU_179	d:Bacteria,p:"Proteobacteria",c:Gammaproteobacteria,o:"Enterobacteriales",f:Enterobacteriaceae	2
OTU_184	d:Bacteria,p:"Bacteroidetes",c:Sphingobacteriia,o:"Sphingobacteriales",f:Sphingobacteriaceae	2
OTU_188	d:Bacteria,p:"Proteobacteria",c:Betaproteobacteria,o:Burkholderiales,f:Burkholderiaceae	2
OTU_189	d:Bacteria,p:"Proteobacteria",c:Betaproteobacteria,o:Burkholderiales,f:Alcaligenaceae	2
OTU_190	d:Bacteria,p:"Actinobacteria",c:Actinobacteria,o:Actinomycetales	2
OTU_191	d:Bacteria,p:"Proteobacteria",c:Betaproteobacteria,o:Burkholderiales,f:Burkholderiaceae	2
OTU_193	d:Bacteria,p:"Acidobacteria",c:Acidobacteria_Gp1	2
OTU_197	d:Bacteria,p:"Proteobacteria",c:Alphaproteobacteria,o:Rhizobiales,f:Hyphomicrobiaceae,g:Hyphomicrobium	2
OTU_204	d:Bacteria,p:"Actinobacteria",c:Actinobacteria,o:Actinomycetales,f:Propionibacteriaceae	2
OTU_209	d:Bacteria,p:"Proteobacteria",c:Gammaproteobacteria,o:"Enterobacteriales",f:Enterobacteriaceae	2
OTU_222	d:Bacteria,p:"Proteobacteria",c:Gammaproteobacteria,o:"Enterobacteriales",f:Enterobacteriaceae	2
OTU_223	d:Bacteria,p:"Actinobacteria",c:Actinobacteria,o:Actinomycetales,f:Streptomycetaceae,g:Streptomyces	2
OTU_238	d:Bacteria,p:"Actinobacteria",c:Actinobacteria,o:Actinomycetales,f:Microbacteriaceae	2
OTU_240	d:Bacteria,p:"Actinobacteria",c:Actinobacteria,o:Actinomycetales,f:Mycobacteriaceae,g:Mycobacterium	2
OTU_247	d:Bacteria,p:"Proteobacteria",c:Alphaproteobacteria,o:Sphingomonadales,f:Sphingomonadaceae	2
OTU_265	d:Bacteria,p:"Proteobacteria",c:Gammaproteobacteria,o:Pseudomonadales,f:Moraxellaceae,g:Acinetobacter	2
OTU_268	d:Bacteria,p:Firmicutes,c:Bacilli,o:Bacillales,f:Bacillaceae_1,g:Bacillus	2
OTU_272	d:Bacteria,p:"Proteobacteria",c:Gammaproteobacteria,o:Xanthomonadales,f:Xanthomonadaceae	2
OTU_281	d:Bacteria,p:"Bacteroidetes",c:Sphingobacteriia,o:"Sphingobacteriales",f:Chitinophagaceae,g:Chitinophaga	2
OTU_316	d:Bacteria,p:"Proteobacteria",c:Gammaproteobacteria,o:Pseudomonadales,f:Pseudomonadaceae,g:Pseudomonas	2
OTU_322	d:Bacteria,p:"Proteobacteria",c:Betaproteobacteria,o:Burkholderiales,f:Burkholderiaceae	2
OTU_325	d:Bacteria,p:"Proteobacteria",c:Betaproteobacteria,o:Burkholderiales	2
OTU_328	d:Bacteria,p:"Proteobacteria",c:Gammaproteobacteria,o:Xanthomonadales,f:Xanthomonadaceae,g:Lysobacter	2
OTU_336	d:Bacteria,p:"Proteobacteria",c:Gammaproteobacteria,o:"Enterobacteriales",f:Enterobacteriaceae	2
OTU_341	d:Bacteria,p:"Actinobacteria",c:Actinobacteria,o:Actinomycetales,f:Microbacteriaceae	2
OTU_356	d:Bacteria,p:"Proteobacteria",c:Alphaproteobacteria,o:Rhizobiales,f:Hyphomicrobiaceae,g:Devosia	2
OTU_359	d:Bacteria,p:"Proteobacteria",c:Alphaproteobacteria,o:Rhizobiales,f:Rhizobiaceae	2
OTU_360	d:Bacteria,p:"Proteobacteria",c:Alphaproteobacteria	2
OTU_361	d:Bacteria,p:"Proteobacteria",c:Gammaproteobacteria,o:Xanthomonadales,f:Xanthomonadaceae	2
OTU_363	d:Bacteria,p:"Proteobacteria",c:Alphaproteobacteria,o:Rhodospirillales,f:Acetobacteraceae	2
OTU_365	d:Bacteria,p:Firmicutes,c:Bacilli,o:Bacillales,f:Paenibacillaceae_1	2
OTU_372	d:Bacteria,p:"Proteobacteria",c:Betaproteobacteria,o:Burkholderiales,f:Burkholderiaceae	2
OTU_381	d:Bacteria,p:"Proteobacteria",c:Betaproteobacteria,o:Burkholderiales	2
OTU_390	d:Bacteria,p:Firmicutes,c:Bacilli,o:Bacillales,f:Bacillaceae_1,g:Bacillus	2
OTU_391	d:Bacteria,p:Firmicutes,c:Bacilli,o:Bacillales,f:Staphylococcaceae,g:Staphylococcus	2
OTU_398	d:Bacteria,p:"Proteobacteria",c:Gammaproteobacteria,o:Xanthomonadales,f:Xanthomonadaceae	2
OTU_26	d:Bacteria,p:"Proteobacteria",c:Betaproteobacteria,o:Burkholderiales,f:Burkholderiaceae	1
OTU_29	d:Bacteria,p:"Actinobacteria",c:Actinobacteria,o:Actinomycetales	1
OTU_31	d:Bacteria,p:"Proteobacteria",c:Gammaproteobacteria,o:"Enterobacteriales",f:Enterobacteriaceae	1
OTU_32	d:Bacteria,p:"Proteobacteria",c:Betaproteobacteria,o:Burkholderiales	1
OTU_33	d:Bacteria,p:"Proteobacteria",c:Alphaproteobacteria,o:Rhizobiales	1
OTU_38	d:Bacteria,p:"Bacteroidetes",c:Sphingobacteriia,o:"Sphingobacteriales",f:Chitinophagaceae	1
OTU_43	d:Bacteria,p:"Actinobacteria",c:Actinobacteria,o:Actinomycetales,f:Microbacteriaceae	1
OTU_44	d:Bacteria,p:"Proteobacteria",c:Betaproteobacteria,o:Burkholderiales	1
OTU_47	d:Bacteria,p:"Bacteroidetes",c:Sphingobacteriia,o:"Sphingobacteriales",f:Chitinophagaceae	1
OTU_54	d:Bacteria,p:Firmicutes,c:Bacilli,o:Bacillales,f:Bacillaceae_1	1
OTU_55	d:Bacteria,p:"Proteobacteria",c:Alphaproteobacteria,o:Caulobacterales,f:Caulobacteraceae	1
OTU_57	d:Bacteria,p:"Proteobacteria",c:Betaproteobacteria,o:Burkholderiales,f:Burkholderiaceae	1
OTU_58	d:Bacteria,p:"Proteobacteria",c:Alphaproteobacteria,o:Rhizobiales	1
OTU_60	d:Bacteria,p:"Proteobacteria",c:Gammaproteobacteria,o:Xanthomonadales,f:Xanthomonadaceae	1
OTU_64	d:Bacteria,p:"Proteobacteria",c:Alphaproteobacteria,o:Rhodospirillales,f:Rhodospirillaceae	1
OTU_70	d:Bacteria,p:"Proteobacteria",c:Gammaproteobacteria,o:"Enterobacteriales",f:Enterobacteriaceae	1
OTU_71	d:Bacteria,p:"Proteobacteria",c:Betaproteobacteria	1
OTU_77	d:Bacteria,p:"Bacteroidetes",c:Sphingobacteriia,o:"Sphingobacteriales",f:Sphingobacteriaceae	1
OTU_78	d:Bacteria,p:"Proteobacteria",c:Betaproteobacteria,o:Burkholderiales	1

doi: 10.3389/fpls.2017.02191

OTU_79	d:Bacteria,p:"Proteobacteria",c:Alphaproteobacteria,o:Rhizobiales	1
OTU_83	d:Bacteria,p:"Proteobacteria",c:Alphaproteobacteria,o:Sphingomonadales,f:Sphingomonadaceae	1
OTU_84	d:Bacteria,p:"Proteobacteria",c:Alphaproteobacteria,o:Rhodospirillales,f:Rhodospirillaceae	1
OTU_91	d:Bacteria,p:"Proteobacteria",c:Gammaproteobacteria	1
OTU_93	d:Bacteria,p:"Proteobacteria",c:Betaproteobacteria,o:Burkholderiales	1
OTU_94	d:Bacteria,p:"Proteobacteria",c:Betaproteobacteria	1
OTU_96	d:Bacteria,p:"Proteobacteria",c:Betaproteobacteria,o:Burkholderiales	1
OTU_98	d:Bacteria,p:"Proteobacteria",c:Alphaproteobacteria,o:Rhizobiales	1
OTU_99	d:Bacteria,p:"Bacteroidetes",c:Sphingobacteriia,o:"Sphingobacteriales",f:Chitinophagaceae	1
OTU_100	d:Bacteria,p:"Bacteroidetes",c:Sphingobacteriia,o:"Sphingobacteriales",f:Chitinophagaceae	1
OTU_101	d:Bacteria,p:"Bacteroidetes",c:Sphingobacteriia,o:"Sphingobacteriales",f:Chitinophagaceae,g:Chitinophaga	1
OTU_103	d:Bacteria,p:"Proteobacteria",c:Betaproteobacteria	1
OTU_104	d:Bacteria,p:"Actinobacteria",c:Actinobacteria,o:Actinomycetales,f:Microbacteriaceae	1
OTU_105	d:Bacteria,p:"Proteobacteria",c:Betaproteobacteria,o:Burkholderiales	1
OTU_106	d:Bacteria,p:"Proteobacteria",c:Alphaproteobacteria,o:Rhizobiales	1
OTU_107	d:Bacteria,p:"Proteobacteria",c:Alphaproteobacteria	1
OTU_108	d:Bacteria,p:Firmicutes,c:Bacilli,o:Bacillales,f:Bacillaceae_1,g:Bacillus	1
OTU_109	d:Bacteria,p:"Proteobacteria",c:Betaproteobacteria,o:Burkholderiales	1
OTU_112	d:Bacteria,p:"Bacteroidetes",c:Sphingobacteriia,o:"Sphingobacteriales",f:Chitinophagaceae	1
OTU_113	d:Bacteria,p:"Actinobacteria",c:Actinobacteria,o:Actinomycetales	1
OTU_114	d:Bacteria,p:"Proteobacteria",c:Alphaproteobacteria,o:Rhizobiales	1
OTU_115	d:Bacteria,p:"Proteobacteria",c:Alphaproteobacteria,o:Rhizobiales,f:Rhizobiaceae	1
OTU_122	d:Bacteria,p:"Proteobacteria",c:Alphaproteobacteria,o:Caulobacterales,f:Caulobacteraceae	1
OTU_124	d:Bacteria,p:"Proteobacteria",c:Gammaproteobacteria,o:"Enterobacteriales",f:Enterobacteriaceae	1
OTU_125	d:Bacteria,p:"Proteobacteria",c:Alphaproteobacteria,o:Caulobacterales,f:Caulobacteraceae	1
OTU_126	d:Bacteria,p:"Proteobacteria",c:Betaproteobacteria,o:Burkholderiales,f:Burkholderiaceae	1
OTU_127	d:Bacteria,p:"Proteobacteria",c:Betaproteobacteria,o:Burkholderiales,f:Alcaligenaceae	1
OTU_128	d:Bacteria,p:Firmicutes,c:Bacilli,o:Bacillales,f:Bacillaceae_1,g:Bacillus	1
OTU_129	d:Bacteria,p:"Bacteroidetes",c:Sphingobacteriia,o:"Sphingobacteriales",f:Chitinophagaceae	1
OTU_131	d:Bacteria,p:Firmicutes,c:Bacilli,o:Bacillales,f:Bacillaceae_1	1
OTU_133	d:Bacteria,p:"Proteobacteria",c:Gammaproteobacteria,o:Xanthomonadales,f:Xanthomonadaceae	1
OTU_140	d:Bacteria,p:"Proteobacteria",c:Gammaproteobacteria,o:"Enterobacteriales",f:Enterobacteriaceae	1
OTU_141	d:Bacteria,p:"Actinobacteria",c:Actinobacteria,o:Actinomycetales,f:Microbacteriaceae	1
OTU_144	d:Bacteria,p:Firmicutes,c:Bacilli,o:Bacillales,f:Bacillaceae_1	1
OTU_145	d:Bacteria,p:Firmicutes,c:Bacilli,o:Bacillales,f:Paenibacillaceae_1,g:Paenibacillus	1
OTU_146	d:Bacteria,p:"Bacteroidetes",c:Sphingobacteriia,o:"Sphingobacteriales"	1
OTU_147	d:Bacteria,p:Firmicutes,c:Bacilli,o:Bacillales,f:Bacillaceae_1	1
OTU_148	d:Bacteria,p:"Proteobacteria",c:Gammaproteobacteria,o:Xanthomonadales,f:Xanthomonadaceae	1
OTU_149	d:Bacteria,p:"Proteobacteria",c:Gammaproteobacteria,o:Xanthomonadales,f:Xanthomonadaceae	1
OTU_152	d:Bacteria,p:"Proteobacteria",c:Gammaproteobacteria	1
OTU_154	d:Bacteria,p:Firmicutes,c:Bacilli,o:Bacillales,f:Bacillaceae_1	1
OTU_156	d:Bacteria,p:Firmicutes,c:Bacilli,o:Bacillales,f:Bacillaceae_1,g:Bacillus	1
OTU_158	d:Bacteria,p:"Bacteroidetes",c:Flavobacteriia,o:"Flavobacteriales",f:Flavobacteriaceae	1
OTU_161	d:Bacteria,p:"Proteobacteria",c:Gammaproteobacteria,o:Pseudomonadales,f:Moraxellaceae	1
OTU_164	d:Bacteria,p:Firmicutes,c:Bacilli,o:Bacillales,f:Bacillaceae_1,g:Bacillus	1
OTU_168	d:Bacteria,p:"Actinobacteria",c:Actinobacteria,o:Actinomycetales,f:Microbacteriaceae	1
OTU_169	d:Bacteria,p:"Proteobacteria",c:Alphaproteobacteria,o:Rhizobiales	1
OTU_171	d:Bacteria,p:"Proteobacteria",c:Alphaproteobacteria,o:Rhizobiales	1
OTU_172	d:Bacteria,p:"Bacteroidetes",c:Sphingobacteriia,o:"Sphingobacteriales",f:Sphingobacteriaceae	1
OTU_173	d:Bacteria,p:"Actinobacteria",c:Actinobacteria,o:Actinomycetales,f:Microbacteriaceae	1
OTU_175	d:Bacteria,p:"Proteobacteria",c:Betaproteobacteria,o:Burkholderiales,f:Burkholderiaceae	1
OTU_176	d:Bacteria,p:"Actinobacteria",c:Actinobacteria,o:Actinomycetales,f:Nocardioidaceae	1
OTU_177	d:Bacteria,p:"Proteobacteria",c:Alphaproteobacteria,o:Rhodospirillales,f:Acetobacteraceae	1
OTU_178	d:Bacteria,p:"Proteobacteria",c:Betaproteobacteria,o:Burkholderiales,f:Burkholderiaceae	1
OTU_183	d:Bacteria,p:Firmicutes,c:Negativicutes,o:Selenomonadales,f:Veillonellaceae,g:Selenomonas	1
OTU_185	d:Bacteria,p:Firmicutes,c:Bacilli,o:Bacillales	1

doi: 10.3389/fpls.2017.02191

OTU_192	d:Bacteria,p:Firmicutes,c:Bacilli,o:Bacillales,f:Paenibacillaceae_1,g:Cohnella	1
OTU_196	d:Bacteria,p:"Actinobacteria",c:Actinobacteria	1
OTU_198	d:Bacteria,p:"Proteobacteria",c:Alphaproteobacteria,o:Rhizobiales,f:Methylobacteriaceae,g:Methylobacterium	1
OTU_199	d:Bacteria,p:"Actinobacteria",c:Actinobacteria,o:Actinomycetales	1
OTU_201	d:Bacteria,p:"Proteobacteria",c:Betaproteobacteria	1
OTU_203	d:Bacteria,p:"Proteobacteria",c:Alphaproteobacteria,o:Rhizobiales	1
OTU_205	d:Bacteria,p:"Proteobacteria",c:Alphaproteobacteria,o:Rhizobiales,f:Bradyrhizobiaceae	1
OTU_206	d:Bacteria,p:"Actinobacteria",c:Actinobacteria,o:Actinomycetales	1
OTU_207	d:Bacteria,p:"Proteobacteria",c:Alphaproteobacteria,o:Rhizobiales,f:Rhizobiaceae	1
OTU_208	d:Bacteria,p:"Proteobacteria",c:Betaproteobacteria,o:Burkholderiales	1
OTU_210	d:Bacteria,p:"Proteobacteria",c:Alphaproteobacteria,o:Rhizobiales	1
OTU_214	d:Bacteria,p:"Bacteroidetes",c:Sphingobacteriia,o:"Sphingobacteriales",f:Chitinophagaceae,g:Chitinophaga	1
OTU_216	d:Bacteria,p:"Proteobacteria",c:Betaproteobacteria	1
OTU_217	d:Bacteria,p:"Actinobacteria",c:Actinobacteria,o:Actinomycetales,f:Actinomycetaceae,g:Actinomyces	1
OTU_218	d:Bacteria,p:Firmicutes,c:Bacilli,o:Bacillales,f:Bacillaceae_1	1
OTU_220	d:Bacteria,p:"Bacteroidetes",c:Flavobacteriia,o:"Flavobacteriales"	1
OTU_221	d:Bacteria,p:"Bacteroidetes",c:Sphingobacteriia,o:"Sphingobacteriales",f:Chitinophagaceae,g:Chitinophaga	1
OTU_224	d:Bacteria,p:Firmicutes,c:Bacilli,o:Bacillales,f:Bacillaceae_1	1
OTU_226	d:Bacteria,p:"Bacteroidetes",c:Cytophagia,o:Cytophagales,f:Cytophagaceae,g:Dyadobacter	1
OTU_227	d:Bacteria,p:"Proteobacteria",c:Alphaproteobacteria,o:Rhizobiales	1
OTU_228	d:Bacteria,p:"Bacteroidetes",c:Sphingobacteriia,o:"Sphingobacteriales",f:Chitinophagaceae	1
OTU_230	d:Bacteria,p:"Proteobacteria",c:Gammaproteobacteria,o:Xanthomonadales,f:Xanthomonadaceae	1
OTU_232	d:Bacteria,p:"Proteobacteria",c:Betaproteobacteria,o:Burkholderiales,f:Burkholderiaceae	1
OTU_233	d:Bacteria,p:"Proteobacteria",c:Betaproteobacteria,o:Burkholderiales,f:Burkholderiaceae	1
OTU_237	d:Bacteria,p:"Actinobacteria",c:Actinobacteria,o:Actinomycetales,f:Microbacteriaceae	1
OTU_239	d:Bacteria,p:"Bacteroidetes",c:Sphingobacteriia	1
OTU_242	d:Bacteria,p:"Actinobacteria",c:Actinobacteria,o:Actinomycetales,f:Micrococcaceae	1
OTU_244	d:Bacteria,p:"Proteobacteria",c:Betaproteobacteria,o:Burkholderiales,f:Burkholderiaceae	1
OTU_245	d:Bacteria,p:"Bacteroidetes",c:Sphingobacteriia,o:"Sphingobacteriales",f:Chitinophagaceae	1
OTU_246	d:Bacteria,p:"Actinobacteria",c:Actinobacteria,o:Actinomycetales,f:Streptomycetaceae,g:Streptomyces	1
OTU_250	d:Bacteria,p:"Proteobacteria",c:Gammaproteobacteria,o:Xanthomonadales,f:Xanthomonadaceae	1
OTU_252	d:Bacteria,p:"Proteobacteria",c:Gammaproteobacteria,o:Xanthomonadales,f:Xanthomonadaceae	1
OTU_253	d:Bacteria,p:"Proteobacteria",c:Betaproteobacteria,o:Burkholderiales,f:Alcaligenaceae	1
OTU_254	d:Bacteria,p:"Actinobacteria",c:Actinobacteria,o:Actinomycetales,f:Microbacteriaceae	1
OTU_255	d:Bacteria,p:"Proteobacteria",c:Gammaproteobacteria,o:Xanthomonadales,f:Xanthomonadaceae	1
OTU_257	d:Bacteria,p:"Proteobacteria",c:Gammaproteobacteria,o:"Enterobacteriales",f:Enterobacteriaceae	1
OTU_258	d:Bacteria,p:"Proteobacteria",c:Alphaproteobacteria,o:Caulobacterales,f:Caulobacteraceae	1
OTU_260	d:Bacteria,p:"Proteobacteria",c:Alphaproteobacteria,o:Rhizobiales	1
OTU_261	d:Bacteria,p:Firmicutes,c:Bacilli,o:Bacillales,f:Bacillaceae_1	1
OTU_263	d:Bacteria,p:Firmicutes,c:Bacilli,o:Bacillales	1
OTU_264	d:Bacteria,p:Firmicutes,c:Clostridia	1
OTU_266	d:Bacteria,p:Firmicutes,c:Bacilli,o:Bacillales,f:Paenibacillaceae_1	1
OTU_269	d:Bacteria,p:Firmicutes,c:Bacilli,o:Bacillales	1
OTU_271	d:Bacteria,p:"Proteobacteria",c:Betaproteobacteria,o:Burkholderiales,f:Oxalobacteraceae	1
OTU_273	d:Bacteria,p:"Proteobacteria",c:Betaproteobacteria,o:Burkholderiales,f:Burkholderiaceae	1
OTU_274	d:Bacteria,p:"Proteobacteria",c:Betaproteobacteria,o:Burkholderiales,f:Burkholderiaceae	1
OTU_275	d:Bacteria,p:Firmicutes,c:Bacilli,o:Bacillales,f:Planococcaceae	1
OTU_276	d:Bacteria,p:"Proteobacteria",c:Betaproteobacteria,o:Burkholderiales	1
OTU_280	d:Bacteria,p:"Proteobacteria",c:Gammaproteobacteria,o:Xanthomonadales,f:Xanthomonadaceae	1
OTU_282	d:Bacteria,p:Firmicutes,c:Bacilli,o:Bacillales,f:Bacillaceae_1,g:Bacillus	1
OTU_283	d:Bacteria,p:Firmicutes,c:Bacilli,o:Bacillales,f:Bacillaceae_1,g:Bacillus	1
OTU_284	d:Bacteria,p:"Proteobacteria",c:Betaproteobacteria,o:Burkholderiales,f:Burkholderiaceae	1
OTU_287	d:Bacteria,p:"Actinobacteria",c:Actinobacteria,o:Actinomycetales,f:Nocardiaceae	1
OTU_289	d:Bacteria,p:"Actinobacteria",c:Actinobacteria,o:Actinomycetales,f:Nocardiaceae	1
OTU_290	d:Bacteria,p:"Proteobacteria",c:Betaproteobacteria,o:Burkholderiales,f:Burkholderiaceae	1
OTU_292	d:Bacteria,p:"Proteobacteria",c:Gammaproteobacteria,o:"Enterobacteriales",f:Enterobacteriaceae	1

doi: 10.3389/fpls.2017.02191

OTU_293	d:Bacteria,p:Firmicutes,c:Bacilli,o:Bacillales,f:Paenibacillaceae_1	1
OTU_295	d:Bacteria,p:Firmicutes,c:Bacilli,o:Bacillales,f:Planococcaceae	1
OTU_296	d:Bacteria,p:Firmicutes,c:Bacilli,o:Bacillales	1
OTU_297	d:Bacteria,p:Firmicutes,c:Bacilli,o:Bacillales	1
OTU_298	d:Bacteria,p:Firmicutes,c:Bacilli,o:Bacillales	1
OTU_299	d:Bacteria,p:Firmicutes,c:Bacilli,o:Bacillales,f:Planococcaceae	1
OTU_300	d:Bacteria,p:Firmicutes,c:Bacilli,o:Bacillales,f:Paenibacillaceae_1	1
OTU_301	d:Bacteria,p:Firmicutes,c:Bacilli,o:Bacillales,f:Planococcaceae	1
OTU_302	d:Bacteria,p:Firmicutes,c:Bacilli,o:Bacillales,f:Bacillaceae_1,g:Bacillus	1
OTU_304	d:Bacteria,p:Firmicutes,c:Bacilli,o:Bacillales,f:Paenibacillaceae_1	1
OTU_305	d:Bacteria,p:Firmicutes,c:Bacilli,o:Bacillales,f:Paenibacillaceae_1,g:Paenibacillus	1
OTU_306	d:Bacteria,p:"Proteobacteria",c:Alphaproteobacteria,o:Rhizobiales,f:Methylobacteriaceae	1
OTU_308	d:Bacteria,p:Firmicutes,c:Bacilli,o:Bacillales,f:Bacillaceae_1,g:Bacillus	1
OTU_309	d:Bacteria,p:Firmicutes,c:Bacilli,o:Bacillales,f:Bacillaceae_1	1
OTU_310	d:Bacteria,p:Firmicutes,c:Bacilli,o:Bacillales,f:Bacillaceae_1,g:Bacillus	1
OTU_311	d:Bacteria,p:Firmicutes,c:Bacilli,o:Bacillales,f:Bacillaceae_1,g:Bacillus	1
OTU_312	d:Bacteria,p:Firmicutes,c:Bacilli,o:Bacillales,f:Paenibacillaceae_1,g:Brevibacillus	1
OTU_313	d:Bacteria,p:"Actinobacteria",c:Actinobacteria,o:Actinomycetales,f:Promicromonosporaceae	1
OTU_314	d:Bacteria,p:Firmicutes,c:Bacilli,o:Bacillales,f:Paenibacillaceae_1,g:Brevibacillus	1
OTU_315	d:Bacteria,p:"Proteobacteria",c:Alphaproteobacteria,o:Rhodospirillales,f:Acetobacteraceae	1
OTU_319	d:Bacteria,p:"Proteobacteria",c:Betaproteobacteria,o:Burkholderiales,f:Burkholderiaceae	1
OTU_320	d:Bacteria,p:"Proteobacteria",c:Betaproteobacteria,o:Burkholderiales,f:Burkholderiaceae	1
OTU_321	d:Bacteria,p:"Proteobacteria",c:Alphaproteobacteria,o:Rhizobiales,f:Methylobacteriaceae,g:Methylobacterium	1
OTU_323	d:Bacteria,p:"Proteobacteria",c:Alphaproteobacteria,o:Rhizobiales	1
OTU_324	d:Bacteria,p:Firmicutes,c:Bacilli,o:Bacillales,f:Paenibacillaceae_1,g:Brevibacillus	1
OTU_327	d:Bacteria,p:"Proteobacteria",c:Gammaproteobacteria,o:Xanthomonadales,f:Xanthomonadaceae	1
OTU_329	d:Bacteria,p:"Proteobacteria",c:Betaproteobacteria,o:Burkholderiales	1
OTU_330	d:Bacteria,p:"Actinobacteria",c:Actinobacteria,o:Actinomycetales,f:Dermabacteraceae	1
OTU_332	d:Bacteria,p:"Actinobacteria",c:Actinobacteria,o:Actinomycetales,f:Nocardioidaceae	1
OTU_333	d:Bacteria,p:"Actinobacteria",c:Actinobacteria,o:Actinomycetales,f:Nocardioidaceae	1
OTU_337	d:Bacteria,p:Firmicutes,c:Bacilli,o:Bacillales,f:Paenibacillaceae_1,g:Brevibacillus	1
OTU_338	d:Bacteria,p:Firmicutes,c:Bacilli,o:Bacillales,f:Paenibacillaceae_1,g:Brevibacillus	1
OTU_339	d:Bacteria,p:"Proteobacteria",c:Alphaproteobacteria,o:Rhizobiales,f:"Aurantimonadaceae"	1
OTU_340	d:Bacteria,p:Firmicutes,c:Bacilli,o:Bacillales,f:Paenibacillaceae_1,g:Brevibacillus	1
OTU_342	d:Bacteria,p:"Proteobacteria",c:Gammaproteobacteria,o:"Enterobacteriales",f:Enterobacteriaceae	1
OTU_343	d:Bacteria,p:"Actinobacteria",c:Actinobacteria,o:Actinomycetales,f:Microbacteriaceae	1
OTU_344	d:Bacteria,p:"Actinobacteria",c:Actinobacteria,o:Actinomycetales,f:Streptomycetaceae	1
OTU_345	d:Bacteria,p:Firmicutes,c:Bacilli,o:Bacillales,f:Paenibacillaceae_1,g:Brevibacillus	1
OTU_346	d:Bacteria,p:Firmicutes,c:Bacilli,o:Bacillales,f:Bacillaceae_1,g:Bacillus	1
OTU_348	d:Bacteria,p:Firmicutes,c:Bacilli,o:Bacillales,f:Bacillaceae_1	1
OTU_349	d:Bacteria,p:"Proteobacteria",c:Alphaproteobacteria,o:Rhizobiales	1
OTU_351	d:Bacteria,p:Firmicutes,c:Bacilli,o:Bacillales,f:Bacillaceae_1	1
OTU_352	d:Bacteria,p:"Proteobacteria",c:Betaproteobacteria,o:Burkholderiales,f:Burkholderiaceae	1
OTU_354	d:Bacteria,p:"Actinobacteria",c:Actinobacteria,o:Actinomycetales	1
OTU_355	d:Bacteria,p:"Actinobacteria",c:Actinobacteria,o:Actinomycetales,f:Promicromonosporaceae	1
OTU_357	d:Bacteria,p:Firmicutes,c:Bacilli,o:Bacillales,f:Paenibacillaceae_1,g:Brevibacillus	1
OTU_358	d:Bacteria,p:Firmicutes,c:Bacilli,o:Bacillales,f:Paenibacillaceae_1,g:Brevibacillus	1
OTU_362	d:Bacteria,p:Firmicutes,c:Bacilli,o:Bacillales,f:Paenibacillaceae_1	1
OTU_366	d:Bacteria,p:"Proteobacteria",c:Alphaproteobacteria,o:Rhizobiales	1
OTU_368	d:Bacteria,p:"Bacteroidetes",c:Sphingobacteriia,o:"Sphingobacteriales",f:Chitinophagaceae,g:Chitinophaga	1
OTU_370	d:Bacteria,p:"Proteobacteria",c:Alphaproteobacteria,o:Rhizobiales	1
OTU_371	d:Bacteria,p:"Proteobacteria",c:Betaproteobacteria,o:Burkholderiales,f:Burkholderiaceae	1
OTU_373	d:Bacteria,p:"Proteobacteria",c:Gammaproteobacteria,o:Pseudomonadales,f:Pseudomonadaceae,g:Pseudomonas	1
OTU_374	d:Bacteria,p:"Proteobacteria",c:Betaproteobacteria,o:Burkholderiales,f:Burkholderiaceae	1
OTU_375	d:Bacteria,p:"Proteobacteria",c:Alphaproteobacteria,o:Sphingomonadales	1
OTU_377	d:Bacteria,p:"Proteobacteria",c:Alphaproteobacteria,o:Rhizobiales	1

doi: 10.3389/fpls.2017.02191

OTU_378	d:Bacteria,p:"Proteobacteria",c:Betaproteobacteria,o:Burkholderiales,f:Burkholderiaceae,g:Pandoraea	1
OTU_379	d:Bacteria,p:"Actinobacteria",c:Actinobacteria,o:Actinomycetales,f:Microbacteriaceae	1
OTU_383	d:Bacteria,p:Firmicutes,c:Bacilli,o:Bacillales,f:Paenibacillaceae_1	1
OTU_384	d:Bacteria,p:"Proteobacteria",c:Alphaproteobacteria,o:Rhizobiales	1
OTU_385	d:Bacteria	1
OTU_386	d:Bacteria,p:"Actinobacteria",c:Actinobacteria,o:Actinomycetales	1
OTU_387	d:Bacteria,p:"Proteobacteria",c:Betaproteobacteria,o:Burkholderiales,f:Comamonadaceae,g:Delftia	1
OTU_388	d:Bacteria,p:"Proteobacteria",c:Gammaproteobacteria,o:Pseudomonadales,f:Moraxellaceae,g:Acinetobacter	1
OTU_389	d:Bacteria,p:"Proteobacteria",c:Gammaproteobacteria,o:"Enterobacteriales",f:Enterobacteriaceae	1
OTU_392	d:Bacteria,p:"Bacteroidetes",c:Sphingobacteriia,o:"Sphingobacteriales",f:Chitinophagaceae,g:Chitinophaga	1
OTU_393	d:Bacteria,p:"Proteobacteria",c:Alphaproteobacteria,o:Rhizobiales	1
OTU_395	d:Bacteria,p:Firmicutes,c:Bacilli,o:Bacillales,f:Planococcaceae	1
OTU_396	d:Bacteria,p:"Proteobacteria",c:Alphaproteobacteria,o:Rhizobiales,f:Xanthobacteraceae,g:Labrys	1
OTU_397	d:Bacteria,p:Firmicutes,c:Bacilli,o:Bacillales,f:Bacillaceae_1,g:Bacillus	1

doi: 10.3389/fpls.2017.02191
