# Supplementary material for: A Community-Based Culture Collection for Targeting Novel Plant Growth-Promoting Bacteria from the Sugarcane Microbiome
Source: Front Plant Sci. 2018 Jan 4;8:2191. doi: 10.3389/fpls.2017.02191 (PMC5759035; doi:10.3389/fpls.2017.02191)
Supplement: Supplementary file 6 [file Table6.pdf]

SUPPLEMENTARY TABLE S6 | Relative abundances of CBC taxa in sugarcane organs. The relative abundances of taxa in sugarcane organs were obtained by summing the abundances of cOTUs, given by cross-referencing the CBC and the sugarcane microbiome datasets. Taxa considered unknown at a given taxonomic level were collapsed to the common deepest taxonomic level. The table is organized by the number of wells in sugarcane CBC. d, domain; p, phylum; c, class; o, order; f, family; g, genus; End, endophytic.

Deepest taxonomic level resolved by "utax" algorithm	Number of wells in sugarcane CBC	Relative abundance of CBC taxa in sugarcane organs											
		Belowground organs			Endophytic aboveground organs					Exophytic aboveground organs			
		Bulk soil	Rhizosphere	End. root	Young Shoot	Bottom stalk	Medium stalk	Upper stalk	Leaf	Bottom stalk	Medium stalk	Upper stalk	Leaf
d:Bacteria,p:Firmicutes,c:Bacilli,o:Bacillales,f:Bacillaceae_1,g:Bacillus	620	0.00094216	0.00141658	0.00050166	0.00061397	0.00143761	0.00037626	0.00037452	0.00137502	0.00105227	0.00086671	0.00010736	0.00110577
d:Bacteria,p:"Proteobacteria",c:Alphaproteobacteria,o:Rhizobiales,f:Rhizobiaceae	496	0.00017077	0.01379132	0.01486336	0.01909941	0.03862232	0.03421754	0.03545046	0.01568107	0.04395250	0.04215458	0.09788767	0.05293798
d:Bacteria,p:"Proteobacteria",c:Gammaproteobacteria,o:Xanthomonadales,f:Xanthomonadaceae	427	0.00603245	0.00309735	0.00452511	0.01367049	0.02446307	0.01464401	0.01474584	0.03549705	0.02435524	0.03897002	0.03439279	0.01300782
d:Bacteria,p:"Bacteroidetes",c:Sphingobacteriia,o:"Sphingobacteriales",f:Chitinophagaceae,g:Chitinophaga	359	0.00116696	0.04090541	0.04720023	0.01688942	0.00294806	0.00315962	0.00261532	0.00164984	0.00511430	0.00584345	0.00305603	0.00006641
d:Bacteria,p:"Proteobacteria",c:Betaproteobacteria,o:Burkholderiales,f:Burkholderiaceae	328	0.00607418	0.00873739	0.01597951	0.03373077	0.04486159	0.01445837	0.01412499	0.00739592	0.02119763	0.02269018	0.01685630	0.01178737
d:Bacteria,p:"Proteobacteria",c:Gammaproteobacteria,o:"Enterobacteriales",f:Enterobacteriaceae	291	0.00044379	0.00226220	0.01284894	0.03296950	0.08279477	0.13442196	0.13726347	0.17484946	0.02467134	0.02411243	0.03312807	0.23412393
d:Bacteria,p:"Proteobacteria",c:Betaproteobacteria,o:Burkholderiales,f:Burkholderiaceae,g:Burkholderia	262	0.00188619	0.00690814	0.01497383	0.03272597	0.04065996	0.00840423	0.00802940	0.00465962	0.02074847	0.02233550	0.01672306	0.01149048
d:Bacteria,p:"Proteobacteria",c:Alphaproteobacteria,o:Rhizobiales	197	0.00017356	0.01405480	0.01506929	0.01970999	0.03888658	0.03462376	0.03587000	0.01574910	0.04445206	0.04245356	0.09855168	0.05297655
d:Bacteria,p:Firmicutes,c:Bacilli,o:Bacillales,f:Paenibacillaceae_1,g:Brevibacillus	145	0.00000612	0.00000878	0.00000625	0.00000793	0.00001658	0.00000000	0.00004003	0.00000000	0.00001977	0.00000383	0.00000018	0.00001083
d:Bacteria,p:"Proteobacteria",c:Alphaproteobacteria,o:Rhizobiales,f:Rhizobiaceae,g:Rhizobium	143	0.00011131	0.00735976	0.00672662	0.00461626	0.00083359	0.00115151	0.00126052	0.00048150	0.00347249	0.00195759	0.00082902	0.00058944
d:Bacteria,p:"Proteobacteria",c:Betaproteobacteria,o:Burkholderiales,f:Comamonadaceae	113	0.00103317	0.00958982	0.01086754	0.00776781	0.00527910	0.00496233	0.00465759	0.01719136	0.00703072	0.00503015	0.00699642	0.01602427
d:Bacteria,p:"Actinobacteria",c:Actinobacteria,o:Actinomycetales,f:Microbacteriaceae	100	0.00006093	0.00059258	0.00048387	0.00100078	0.00358174	0.00110317	0.00072261	0.00204882	0.02191904	0.02055748	0.00776265	0.00389882
d:Bacteria,p:"Proteobacteria",c:Betaproteobacteria,o:Burkholderiales	95	0.00687584	0.01704725	0.02830521	0.02480531	0.01778106	0.02837048	0.03136710	0.02903358	0.01376024	0.00967811	0.02557700	0.03787109
d:Bacteria,p:Firmicutes,c:Bacilli,o:Bacillales,f:Planococcaceae	90	0.00298250	0.00208202	0.00093326	0.00129506	0.00172120	0.00060788	0.00066581	0.00090446	0.00268556	0.00139393	0.00045890	0.00302909
d:Bacteria,p:"Bacteroidetes",c:Sphingobacteriia,o:"Sphingobacteriales",f:Sphingobacteriaceae	88	0.00146462	0.02244721	0.00867014	0.01923048	0.01759642	0.02585413	0.02620906	0.02393637	0.01499240	0.01143958	0.05613852	0.00875553
d:Bacteria,p:Firmicutes,c:Bacilli,o:Bacillales,f:Bacillaceae_1	84	0.00303252	0.00280015	0.00109886	0.00161329	0.00216299	0.00086338	0.00090259	0.00183428	0.00311503	0.00198174	0.00050785	0.00351811
d:Bacteria,p:"Proteobacteria",c:Gammaproteobacteria	76	0.00349285	0.00890456	0.01661572	0.01551371	0.04129878	0.02175892	0.02084236	0.04150488	0.04795295	0.05497552	0.03893125	0.01666530
d:Bacteria,p:"Actinobacteria",c:Actinobacteria,o:Actinomycetales,f:Promicromonosporaceae	65	0.00000000	0.00000021	0.00000021	0.00000105	0.00000100	0.00001029	0.00000000	0.00000000	0.00001539	0.00001586	0.00000035	0.00000156
d:Bacteria,p:"Actinobacteria",c:Actinobacteria,o:Actinomycetales	63	0.00005527	0.00042825	0.00035078	0.00078105	0.00337307	0.00095911	0.00059614	0.00204760	0.02207706	0.01933248	0.00729403	0.00384359
d:Bacteria,p:"Proteobacteria",c:Gammaproteobacteria,o:Xanthomonadales,f:Xanthomonadaceae,g:Lysobacter	61	0.00020336	0.00030647	0.00013100	0.00035545	0.00006837	0.00020888	0.00012747	0.00003226	0.00007335	0.00004164	0.00000799	0.00006493
d:Bacteria,p:"Bacteroidetes",c:Flavobacteriia,o:"Flavobacteriales",f:Flavobacteriaceae	55	0.00008098	0.00370365	0.00306977	0.00320939	0.00419811	0.00520731	0.00442378	0.01019239	0.00496307	0.00407573	0.00318199	0.00338403
d:Bacteria,p:"Bacteroidetes",c:Sphingobacteriia,o:"Sphingobacteriales",f:Chitinophagaceae	43	0.00112294	0.03769843	0.04210492	0.02294081	0.00289021	0.00319760	0.00263931	0.00128296	0.00496982	0.00517730	0.00290201	0.00004663
d:Bacteria,p:"Proteobacteria",c:Alphaproteobacteria,o:Rhodospirillales,f:Rhodospirillaceae,g:Inquilinus	40	0.00000664	0.00047595	0.00042293	0.00012312	0.00000651	0.00000969	0.00000850	0.00006332	0.00000720	0.00000222	0.00000036	0.00000000
d:Bacteria,p:Firmicutes,c:Bacilli,o:Bacillales	39	0.00298250	0.00208202	0.00093326	0.00129506	0.00172120	0.00060788	0.00066581	0.00090446	0.00268556	0.00139393	0.00045890	0.00302909
d:Bacteria,p:"Actinobacteria",c:Actinobacteria,o:Actinomycetales,f:Micrococcaceae	38	0.00000644	0.00001238	0.00000427	0.00000673	0.00001242	0.00000092	0.00002204	0.00003166	0.00004029	0.00000748	0.00000111	0.00003005
d:Bacteria,p:"Proteobacteria",c:Gammaproteobacteria,o:Pseudomonadales,f:Moraxellaceae,g:Acinetobacter	34	0.00003198	0.00267251	0.00493534	0.00684537	0.17517018	0.27000400	0.29381279	0.17549670	0.02720329	0.01016078	0.00101339	0.10237334
d:Bacteria,p:"Proteobacteria",c:Betaproteobacteria	32	0.00115744	0.00425819	0.00893362	0.01385860	0.00724870	0.01046388	0.01081850	0.00744736	0.00370171	0.00174376	0.01005346	0.01875639
d:Bacteria,p:"Proteobacteria",c:Alphaproteobacteria,o:Caulobacterales,f:Caulobacteraceae	30	0.00008641	0.00986121	0.00959777	0.00214884	0.00155083	0.00248615	0.00236998	0.00102937	0.00078759	0.00018954	0.00006374	0.00017117
d:Bacteria,p:"Bacteroidetes",c:Sphingobacteriia,o:"Sphingobacteriales",f:Sphingobacteriaceae,g:Mucilaginibacter	29	0.00024849	0.01013785	0.00321988	0.00402905	0.00021176	0.00022198	0.00009501	0.00019762	0.00105841	0.00091829	0.00017727	0.00005268
d:Bacteria,p:"Proteobacteria",c:Betaproteobacteria,o:Burkholderiales,f:Oxalobacteraceae	29	0.00128652	0.00247527	0.00450797	0.00145612	0.01173871	0.01202855	0.01258111	0.01047426	0.01077761	0.01757888	0.01026034	0.02021352
d:Bacteria,p:"Actinobacteria",c:Actinobacteria,o:Actinomycetales,f:Microbacteriaceae,g:Curtobacterium	29	0.00005809	0.00043221	0.00035986	0.00086120	0.00306191	0.00090232	0.00058288	0.00198310	0.01839689	0.01711564	0.00671128	0.00382177
d:Bacteria,p:Firmicutes,c:Bacilli,o:Bacillales,f:Paenibacillaceae_1	26	0.00312357	0.00163507	0.00076348	0.00118072	0.00180428	0.00072824	0.00074792	0.00111665	0.00259614	0.00160744	0.00052018	0.00270241
d:Bacteria,p:"Actinobacteria",c:Actinobacteria,o:Actinomycetales,f:Streptomycetaceae,g:Streptomyces	25	0.00056125	0.00175038	0.00463184	0.00034232	0.00067570	0.00019355	0.00016451	0.00000394	0.00270998	0.00229153	0.00061751	0.00013292
d:Bacteria,p:"Proteobacteria",c:Alphaproteobacteria	24	0.00127899	0.01772036	0.01932585	0.03674119	0.01981545	0.01655090	0.01728126	0.01552557	0.03291949	0.03482608	0.04797674	0.05034248
d:Bacteria,p:"Proteobacteria",c:Alphaproteobacteria,o:Sphingomonadales,f:Sphingomonadaceae	23	0.00125846	0.00843532	0.00679735	0.01139576	0.02266931	0.01374389	0.01338598	0.02865505	0.01851584	0.02298181	0.00958305	0.03541341
d:Bacteria,p:"Proteobacteria",c:Alphaproteobacteria,o:Rhizobiales,f:Beijerinckiaceae	23	0.00010063	0.00669211	0.00894724	0.01516399	0.03315063	0.03044919	0.03134502	0.01435297	0.03401595	0.03310825	0.08903919	0.05204676
d:Bacteria,p:"Proteobacteria",c:Gammaproteobacteria,o:Pseudomonadales,f:Pseudomonadaceae	22	0.00103500	0.00422322	0.01216925	0.01463895	0.08408878	0.05454099	0.05801442	0.14777401	0.00586958	0.00313905	0.00390509	0.00381916
d:Bacteria,p:"Proteobacteria",c:Alphaproteobacteria,o:Sphingomonadales,f:Sphingomonadaceae,g:Sphingomonas	20	0.00119063	0.00814693	0.00661168	0.01078664	0.02226082	0.01347286	0.01315063	0.02862942	0.01723841	0.02021586	0.00940522	0.03538043
d:Bacteria,p:"Proteobacteria",c:Alphaproteobacteria,o:Rhizobiales,f:Methylobacteriaceae,g:Methylobacterium	20	0.00001139	0.00000526	0.00002551	0.00014667	0.00245100	0.00072697	0.00060069	0.00143526	0.00463862	0.00442104	0.00199883	0.00087152
d:Bacteria,p:"Actinobacteria",c:Actinobacteria,o:Actinomycetales,f:Microbacteriaceae,g:Microbacterium	20	0.00000054	0.00000494	0.00000294	0.00002323	0.00023254	0.00002088	0.00002026	0.00095061	0.00035856	0.00029416	0.00021909	0.00251883
d:Bacteria,p:Firmicutes,c:Bacilli,o:Bacillales,f:Paenibacillaceae_1,g:Paenibacillus	17	0.00000284	0.00000339	0.00000242	0.00010451	0.00123618	0.00032028	0.00047321	0.00022907	0.00320935	0.00269987	0.00093099	0.00002482
d:Bacteria,p:"Bacteroidetes",c:Sphingobacteriia	15	0.00005426	0.00005309	0.00005445	0.00000000	0.00005299	0.00000212	0.00000000	0.00000000	0.00153291	0.00012468	0.00002974	0.00000030
d:Bacteria,p:"Proteobacteria",c:Gammaproteobacteria,o:Pseudomonadales,f:Pseudomonadaceae,g:Pseudomonas	14	0.00101695	0.00404274	0.01313831	0.01630855	0.13406074	0.07318209	0.07709817	0.16834927	0.00641933	0.00409309	0.00617932	0.03777491
d:Bacteria,p:"Actinobacteria",c:Actinobacteria,o:Actinomycetales,f:Micrococcaceae,g:Arthrobacter	13	0.00000008	0.00000436	0.00000173	0.00000395	0.00000076	0.00000000	0.00000000	0.00000000	0.00000600	0.00000047	0.00000018	0.00000022
d:Bacteria,p:"Proteobacteria",c:Alphaproteobacteria,o:Sphingomonadales	12	0.00003055	0.00407343	0.00582357	0.01373786	0.00995095	0.00800471	0.00730073	0.01103931	0.01136215	0.01053205	0.00959524	0.00087498
d:Bacteria,p:"Proteobacteria",c:Alphaproteobacteria,o:Caulobacterales,f:Caulobacteraceae,g:Asticcacaulis	11	0.00003449	0.00609457	0.00589965	0.00107725	0.00028666	0.00032171	0.00021692	0.00021271	0.00031126	0.00002937	0.00000390	0.00001716
d:Bacteria,p:"Bacteroidetes",c:Cytophagia,o:Cytophagales,f:Cytophagaceae,g:Dyadobacter	11	0.00000218	0.00107452	0.00088024	0.00125000	0.00209820	0.00156859	0.00129292	0.00008485	0.00342449	0.00468754	0.00150638	0.00002466
d:Bacteria,p:"Proteobacteria",c:Betaproteobacteria,o:Burkholderiales,f:Alcaligenaceae	11	0.00011397	0.00018986	0.00049714	0.00206755	0.00725102	0.01040727	0.01227088	0.00220748	0.01442292	0.01150727	0.01515017	0.00536929
d:Bacteria,p:"Proteobacteria",c:Alphaproteobacteria,o:Rhizobiales,f:Bradyrhizobiaceae,g:Bosea	9	0.00000013	0.00000590	0.00000874	0.00001631	0.00001361	0.00005710	0.00002370	0.00000799	0.00005173	0.00003581	0.00002382	0.00000295
d:Bacteria,p:"Actinobacteria",c:Actinobacteria,o:Actinomycetales,f:Nocardioidaceae	9	0.00000156	0.00002824	0.00002607	0.00000370	0.00003360	0.00000948	0.00000686	0.00000000	0.00015512	0.00009987	0.00007276	0.00000510
d:Bacteria,p:"Bacteroidetes",c:Sphingobacteriia,o:"Sphingobacteriales",f:Sphingobacteriaceae,g:Pedobacter	7	0.00000275	0.00316612	0.00195492	0.00756754	0.00715498	0.01686822	0.01607451	0.01549692	0.00844272	0.00555254	0.04049985	0.00129180
d:Bacteria,p:"Proteobacteria"	7	0.00000244	0.00088077	0.00096922	0.00127843	0.00000609	0.00004433	0.00004011	0.00000758	0.00008113	0.00001496	0.00000009	0.00000049
d:Bacteria,p:"Actinobacteria",c:Actinobacteria,o:Solirubrobacterales,f:Patulibacteraceae,g:Patulibacter	7	0.00000000	0.00000000	0.00000000	0.00000011	0.00002150	0.00003214	0.00002298	0.00000000	0.00004674	0.00002639	0.00001321	0.00000314
d:Bacteria,p:"Proteobacteria",c:Alphaproteobacteria,o:Caulobacterales,f:Caulobacteraceae,g:Caulobacter	6	0.00020383	0.00026457	0.00072232	0.00064762	0.00045247	0.00075290	0.00073115	0.00031852	0.00045383	0.00009511	0.00017241	0.00013828
d:Bacteria,p:"Proteobacteria",c:Alphaproteobacteria,o:Rhizobiales,f:Methylobacteriaceae	6	0.00000827	0.00000287	0.00002317	0.00014381	0.00245000	0.00072697	0.00060069	0.00142770	0.00462552	0.00441120	0.00197837	0.00085873
d:Bacteria,p:Firmicutes,c:Bacilli,o:Bacillales,f:Paenibacillaceae_1,g:Cohnella	6	0.00090677	0.00012731	0.00006677	0.00004074	0.00011383	0.00012562	0.00008237	0.00021888	0.00034817	0.00039603	0.00006907	0.00006331
d:Bacteria,p:"Proteobacteria",c:Alphaproteobacteria,o:Caulobacterales,f:Caulobacteraceae,g:Brevundimonas	6	0.00000693	0.00000999	0.00001779	0.00008548	0.00263860	0.00372225	0.00143649	0.00287922	0.00600548	0.00848087	0.00657681	0.00072883
d:Bacteria,p:"Proteobacteria",c:Alphaproteobacteria,o:Rhizobiales,f:Xanthobacteraceae,g:Labrys	6	0.00001194	0.00207154	0.00138292	0.00298273	0.00009680	0.00008502	0.00012488	0.00008776	0.00069998	0.00030040	0.00011022	0.00002009
d:Bacteria,p:"Proteobacteria",c:Alphaproteobacteria,o:Rhodospirillales,f:Rhodospirillaceae	5	0.00000664	0.00047595	0.00042293	0.00012312	0.00000651	0.00000969	0.00000850	0.00006332	0.00000720	0.00000222	0.00000036	0.00000000
d:Bacteria,p:"Proteobacteria",c:Gammaproteobacteria,o:Xanthomonadales	5	0.00007145	0.00021918	0.00014151	0.00054118	0.01542759	0.00534625	0.00528616	0.00275605	0.02681096	0.02403009	0.01384894	0.00321331
d:Bacteria,p:"Actinobacteria",c:Actinobacteria,o:Actinomycetales,f:Streptomycetaceae	5	0.00055887	0.00174250	0.00462445	0.00034195	0.00067570	0.00019355	0.00016451	0.00000394	0.00270732	0.00229096	0.00061751	0.00013292
d:Bacteria,p:"Proteobacteria",c:Alphaproteobacteria,o:Sphingomonadales,f:Sphingomonadaceae,g:Novosphingobium	5	0.00000814	0.00027492	0.00057166	0.00049521	0.00024928	0.00041140	0.00038360	0.00008787	0.00026550	0.00012814	0.00079130	0.00011148
d:Bacteria,p:"Proteobacteria",c:Betaproteobacteria,o:Burkholderiales,f:Comamonadaceae,g:Variovorax	4	0.00093388	0.00459649	0.00599919	0.00583212	0.00423352	0.00274099	0.00257207	0.01559252	0.00579571	0.00416557	0.00646723	0.01567403
d:Bacteria,p:"Proteobacteria",c:Alphaproteobacteria,o:Rhizobiales,f:Phyllobacteriaceae	4	0.00058335	0.00797308	0.00413107	0.00162855	0.00116224	0.00018094	0.00011222	0.00256953	0.00136516	0.00105750	0.00018253	0.00059575
d:Bacteria,p:"Proteobacteria",c:Alphaproteobacteria,o:Rhodospirillales,f:Acetobacteraceae	4	0.00000027	0.00023725	0.00024217	0.00597269	0.01318336	0.00567199	0.00527227	0.00114183	0.01380441	0.02375733	0.01794880	0.00300081
d:Bacteria,p:"Acidobacteria",c:Acidobacteria_Gp1	3	0.00009666	0.00440251	0.00620867	0.00970416	0.00687095	0.00784487	0.00879625	0.00064613	0.01031244	0.01090337	0.01442145	0.00032183
d:Bacteria,p:"Acidobacteria",c:Acidobacteria_Gp1,g:Terriglobus	3	0.00000016	0.00258728	0.00401336	0.00434037	0.00047121	0.00003826	0.00018427	0.00002435	0.00012109	0.00007944	0.00006886	0.00001361
d:Bacteria,p:"Proteobacteria",c:Gammaproteobacteria,o:Xanthomonadales,f:Xanthomonadaceae,g:Dokdonella	3	0.00009790	0.00059922	0.00211111	0.00079494	0.00006404	0.00004340	0.00004127	0.00000433	0.00008602	0.00001911	0.00000045	0.00001024
d:Bacteria,p:"Proteobacteria",c:Betaproteobacteria,o:Burkholderiales,f:Alcaligenaceae,g:Advenella	3	0.00003523	0.00003858	0.00003679	0.00009482	0.00061515	0.00144159	0.00068844	0.00000194	0.00035387	0.00019534	0.00629142	0.00016015
d:Bacteria,p:Firmicutes,c:Bacilli,o:Bacillales,f:Paenibacillaceae_2	3	0.00021614	0.00007041	0.00005517	0.00017334	0.00005427	0.00004720	0.00004703	0.00002616	0.00036433	0.00012701	0.00001880	0.00063084
d:Bacteria,p:"Actinobacteria",c:Actinobacteria,o:Actinomycetales,f:Dermabacteraceae	3	0.00000000	0.00000000	0.00000000	0.00000000	0.00000234	0.00001332	0.00000000	0.00000000	0.00003919	0.00000378	0.00005646	0.00003820
d:Bacteria,p:"Proteobacteria",c:Alphaproteobacteria,o:Rhizobiales,f:Bradyrhizobiaceae	2	0.00000013	0.00000590	0.00000874	0.00001631	0.00001361	0.00005710	0.00002370	0.00000799	0.00005173	0.00003581	0.00002382	0.00000295
d:Bacteria,p:"Bacteroidetes",c:Flavobacteriia,o:"Flavobacteriales",f:Flavobacteriaceae,g:Flavobacterium	2	0.00000035	0.00007991	0.00011641	0.00044377	0.00002218	0.00030906	0.00028657	0.00023538	0.00000608	0.00000000	0.00000018	0.00000236
d:Bacteria,p:"Proteobacteria",c:Alphaproteobacteria,o:Rhizobiales,f:Xanthobacteraceae	2	0.00000105	0.00007453	0.00015139	0.00070085	0.00014554	0.00007090	0.00007039	0.00006884	0.00035590	0.00030899	0.00030515	0.00004764
d:Bacteria,p:"Proteobacteria",c:Gammaproteobacteria,o:Pseudomonadales	2	0.00061582	0.00730257	0.01480591	0.00733345	0.02427494	0.01494819	0.01381632	0.03758482	0.01779956	0.02854802	0.02439561	0.01311892
d:Bacteria,p:"Proteobacteria",c:Alphaproteobacteria,o:Rhizobiales,f:Hyphomicrobiaceae,g:Hyphomicrobium	2	0.00030896	0.00024980	0.00041576	0.00213531	0.00013057	0.00005685	0.00005059	0.00004709	0.00038712	0.00006927	0.00001129	0.00001094
d:Bacteria,p:"Actinobacteria",c:Actinobacteria,o:Actinomycetales,f:Mycobacteriaceae,g:Mycobacterium	2	0.00004254	0.00029124	0.00056001	0.00080270	0.00026984	0.00032054	0.00039419	0.00016341	0.00287381	0.00083544	0.00018421	0.00003651
d:Bacteria,p:"Proteobacteria",c:Alphaproteobacteria,o:Rhizobiales,f:Phyllobacteriaceae,g:Mesorhizobium	2	0.00030951	0.00779403	0.00403770	0.00160057	0.00012739	0.00012378	0.00008584	0.00004601	0.00104565	0.00067503	0.00005870	0.00009428
d:Bacteria,p:Firmicutes,c:Bacilli	2	0.00000194	0.00000186	0.00000164	0.00000082	0.00000000	0.00000000	0.00000000	0.00000000	0.00000582	0.00000941	0.00000062	0.00001677
d:Bacteria,p:"Proteobacteria",c:Alphaproteobacteria,o:Rhizobiales,f:Hyphomicrobiaceae,g:Devosia	2	0.00000031	0.00002249	0.00003685	0.00000477	0.00074257	0.00018768	0.00008299	0.00001033	0.00169110	0.00169044	0.00084784	0.00010432
d:Bacteria,p:"Proteobacteria",c:Alphaproteobacteria,o:Rhizobiales,f:"Aurantimonadaceae"	1	0.00000058	0.00000301	0.00000358	0.00000672	0.00011984	0.00015832	0.00006957	0.00007891	0.00127902	0.00135275	0.00067160	0.00010440
d:Bacteria,p:"Bacteroidetes",c:Sphingobacteriia,o:"Sphingobacteriales"	1	0.00000275	0.00316612	0.00195492	0.00756754	0.00715498	0.01686822	0.01607451	0.01549692	0.00844272	0.00555254	0.04049985	0.00129180
d:Bacteria,p:"Actinobacteria",c:Actinobacteria,o:Actinomycetales,f:Propionibacteriaceae	1	0.00000320	0.00000088	0.00000015	0.00000032	0.00000000	0.00000000	0.00000000	0.00000000	0.00000079	0.00000000	0.00000000	0.00000064
d:Bacteria,p:"Actinobacteria",c:Actinobacteria,o:Actinomycetales,f:Actinomycetaceae,g:Actinomyces	1	0.00000049	0.00000075	0.00000013	0.00000305	0.00010292	0.00026318	0.00005872	0.00000952	0.00011010	0.00000474	0.00001090	0.00007213
d:Bacteria,p:Firmicutes,c:Negativicutes,o:Selenomonadales,f:Veillonellaceae,g:Selenomonas	1	0.00000000	0.00000000	0.00000000	0.00000000	0.00000273	0.00000000	0.00000000	0.00000000	0.00000000	0.00000000	0.00000000	0.00001940
d:Bacteria,p:"Actinobacteria",c:Actinobacteria,o:Actinomycetales,f:Nocardioidaceae,g:Aeromicrobium	1	0.00000127	0.00002321	0.00001658	0.00000359	0.00003260	0.00000948	0.00000686	0.00000000	0.00015293	0.00009947	0.00007241	0.00000441
d:Bacteria,p:"Actinobacteria",c:Actinobacteria,o:Actinomycetales,f:Nocardiaceae	1	0.00000338	0.00001002	0.00001649	0.00000889	0.00000000	0.00000000	0.00000212	0.00000000	0.00001936	0.00000472	0.00000000	0.00000000
d:Bacteria,p:"Proteobacteria",c:Alphaproteobacteria,o:Rhizobiales,f:Brucellaceae	1	0.00006763	0.00324213	0.00293501	0.00208898	0.00158218	0.00214897	0.00072561	0.00049057	0.00589041	0.00618664	0.01037983	0.00459133
d:Bacteria,p:"Actinobacteria",c:Actinobacteria,o:Actinomycetales,f:Propionibacteriaceae,g:Microlunatus	1	0.00000007	0.00000000	0.00000015	0.00000000	0.00000000	0.00000000	0.00000000	0.00000000	0.00000085	0.00000000	0.00000000	0.00000000

doi: 10.3389/fpls.2017.02191
