# Supplementary material for: A Community-Based Culture Collection for Targeting Novel Plant Growth-Promoting Bacteria from the Sugarcane Microbiome
Source: Front Plant Sci. 2018 Jan 4;8:2191. doi: 10.3389/fpls.2017.02191 (PMC5759035; doi:10.3389/fpls.2017.02191)
Supplement: Supplementary file 7 [file Table7.pdf]

SUPPLEMENTARY TABLE S7 | Raw measurements data of inoculated and uninoculated maize plants. Fresh and dry weight of roots, stems and total weight of maize plants after four weeks of growth.

Treatment	Plant	Fresh weight (g)			Dry weight (g)		
		Root	Stem	Total	Root	Stem	Total
Uninoculated	1	1.170	1.370	2.540	0.110	0.147	0.257
	2	1.130	1.280	2.410	0.094	0.133	0.227
	3	1.590	1.740	3.330	0.135	0.168	0.303
	4	0.660	0.520	1.180	0.106	0.062	0.168
	5	1.100	1.270	2.370	0.096	0.135	0.231
	6	1.300	1.280	2.580	0.104	0.103	0.207
	7	1.010	1.100	2.110	0.108	0.127	0.235
	8	0.910	1.080	1.990	0.110	0.099	0.209
Inoculated	1	3.000	3.190	6.190	0.273	0.320	0.593
	2	3.130	3.110	6.240	0.259	0.281	0.540
	3	3.980	4.230	8.210	0.354	0.371	0.725
	4	3.140	3.510	6.650	0.270	0.323	0.594
	5	3.730	4.030	7.760	0.294	0.376	0.670
	6	6.620	7.030	13.650	0.572	0.666	1.238
	7	3.720	3.230	6.950	0.301	0.293	0.594
	8	3.210	3.680	6.890	0.268	0.346	0.615

doi: 10.3389/fpls.2017.02191
