# Supplementary material for: A Community-Based Culture Collection for Targeting Novel Plant Growth-Promoting Bacteria from the Sugarcane Microbiome
Source: Front Plant Sci. 2018 Jan 4;8:2191. doi: 10.3389/fpls.2017.02191 (PMC5759035; doi:10.3389/fpls.2017.02191)
Supplement: Supplementary file 8 [file Table8.pdf]

SUPPLEMENTARY TABLE S8 | Taxonomic assignment and confidence score of OTUs in the synthetic community. OTUs were identified using the SINTAX algorithm in USEARCH and the SILVA database. Conf, confidence.

OTU	SINTAX													
	Kingdom	Conf.	Phylum	Conf.	Class	Conf.	Order	Conf.	Family	Conf.	Genus	Conf.	Species	Conf.
OTU_17	Bacteria	1.0000	Proteobacteria	1.0000	Alphaproteobacteria	1.0000	Rhizobiales	1.0000	Bradyrhizobiaceae	1.0000	Bosea	1.0000	Afipia_genosp._9	0.4100
OTU_23	Bacteria	1.0000	Bacteroidetes	1.0000	Sphingobacteriia	1.0000	Sphingobacteriales	1.0000	Chitinophagaceae	1.0000	Chitinophaga	1.0000	Chitinophaga_sp._MJM_38	1.0000
OTU_21	Bacteria	1.0000	Proteobacteria	1.0000	Betaproteobacteria	1.0000	Burkholderiales	1.0000	Comamonadaceae	1.0000	Ottowia	0.6000	Acidovorax_avenae	0.6000
OTU_16	Bacteria	1.0000	Proteobacteria	1.0000	Alphaproteobacteria	1.0000	Caulobacterales	1.0000	Caulobacteraceae	1.0000	Asticcacaulis	1.0000	Asticcacaulis_sp._A1-31	0.5400
OTU_4	Bacteria	1.0000	Proteobacteria	1.0000	Alphaproteobacteria	1.0000	Sphingomonadales	1.0000	Sphingomonadaceae	0.9900	Sphingomonas	0.9900	Sphingomonas_sp._AHM7	0.2300
OTU_25	Bacteria	1.0000	Actinobacteria	1.0000	Actinobacteria	1.0000	Micrococcales	1.0000	Microbacteriaceae	1.0000	Microbacterium	1.0000	Microbacterium_resistens	0.8300
OTU_49	Bacteria	1.0000	Proteobacteria	1.0000	Gammaproteobacteria	1.0000	Enterobacteriales	0.9900	Enterobacteriaceae	0.9900	Enterobacter	0.8700	Enterobacter_cloacae	0.2900
OTU_8	Bacteria	1.0000	Proteobacteria	1.0000	Alphaproteobacteria	1.0000	Rhizobiales	1.0000	Rhizobiaceae	1.0000	Rhizobium	1.0000	Rhizobium_larrymoorei	0.6000
OTU_3	Bacteria	1.0000	Proteobacteria	1.0000	Gammaproteobacteria	1.0000	Enterobacteriales	1.0000	Enterobacteriaceae	1.0000	Pantoea	0.8100	Pantoea_sp._CBMB23	0.2700
OTU_9	Bacteria	1.0000	Bacteroidetes	1.0000	Sphingobacteriia	1.0000	Sphingobacteriales	1.0000	Sphingobacteriaceae	1.0000	Pedobacter	0.9100	Bacteroidetes_bacterium_EC2	0.9100
OTU_18	Bacteria	1.0000	Proteobacteria	1.0000	Betaproteobacteria	1.0000	Burkholderiales	1.0000	Burkholderiaceae	1.0000	Burkholderia	1.0000	Burkholderia_sp._UYPR1.3	0.3900
OTU_32	Bacteria	1.0000	Bacteroidetes	1.0000	Sphingobacteriia	1.0000	Sphingobacteriales	1.0000	Sphingobacteriaceae	1.0000	Pedobacter	1.0000	bacterium_Wuba47	0.9100
OTU_12	Bacteria	1.0000	Proteobacteria	1.0000	Gammaproteobacteria	1.0000	Xanthomonadales	1.0000	Xanthomonadaceae	1.0000	Lysobacter	1.0000	unidentified	0.6500
OTU_2	Bacteria	1.0000	Proteobacteria	1.0000	Alphaproteobacteria	1.0000	Rhizobiales	1.0000	Rhizobiaceae	0.9800	Ensifer	0.9600	Sinorhizobium_fredii	0.9000
OTU_6	Bacteria	1.0000	Proteobacteria	1.0000	Gammaproteobacteria	1.0000	Xanthomonadales	1.0000	Xanthomonadaceae	1.0000	Dyella	0.6200	Dyella_ginsengisoli	0.4700
OTU_13	Bacteria	1.0000	Proteobacteria	1.0000	Gammaproteobacteria	1.0000	Xanthomonadales	1.0000	Xanthomonadaceae	1.0000	Stenotrophomonas	1.0000	Stenotrophomonas_maltophilia	0.9800
OTU_5	Bacteria	1.0000	Proteobacteria	1.0000	Gammaproteobacteria	1.0000	Xanthomonadales	1.0000	Xanthomonadaceae	1.0000	Pseudoxanthomonas	1.0000	Pseudoxanthomonas_sp._2362	1.0000
OTU_1612	Bacteria	1.0000	Proteobacteria	1.0000	Gammaproteobacteria	1.0000	Xanthomonadales	1.0000	Xanthomonadaceae	1.0000	Stenotrophomonas	1.0000	Stenotrophomonas_sp._AHL_1	0.3600
OTU_15	Bacteria	1.0000	Proteobacteria	1.0000	Gammaproteobacteria	1.0000	Xanthomonadales	1.0000	Xanthomonadaceae	1.0000	Dyella	1.0000	Dyella_sp._JP1	0.8200
OTU_10	Bacteria	1.0000	Actinobacteria	1.0000	Actinobacteria	1.0000	Streptomycetales	1.0000	Streptomycetaceae	1.0000	Streptomyces	0.7500	Streptomyces_sp._QLS01	0.3600

doi: 10.3389/fpls.2017.02191
