# Supplementary material for: A Community-Based Culture Collection for Targeting Novel Plant Growth-Promoting Bacteria from the Sugarcane Microbiome
Source: Front Plant Sci. 2018 Jan 4;8:2191. doi: 10.3389/fpls.2017.02191 (PMC5759035; doi:10.3389/fpls.2017.02191)
Supplement: Supplementary file 9 [file Image1.pdf]

doi: 10.3389/fpls.2017.02191

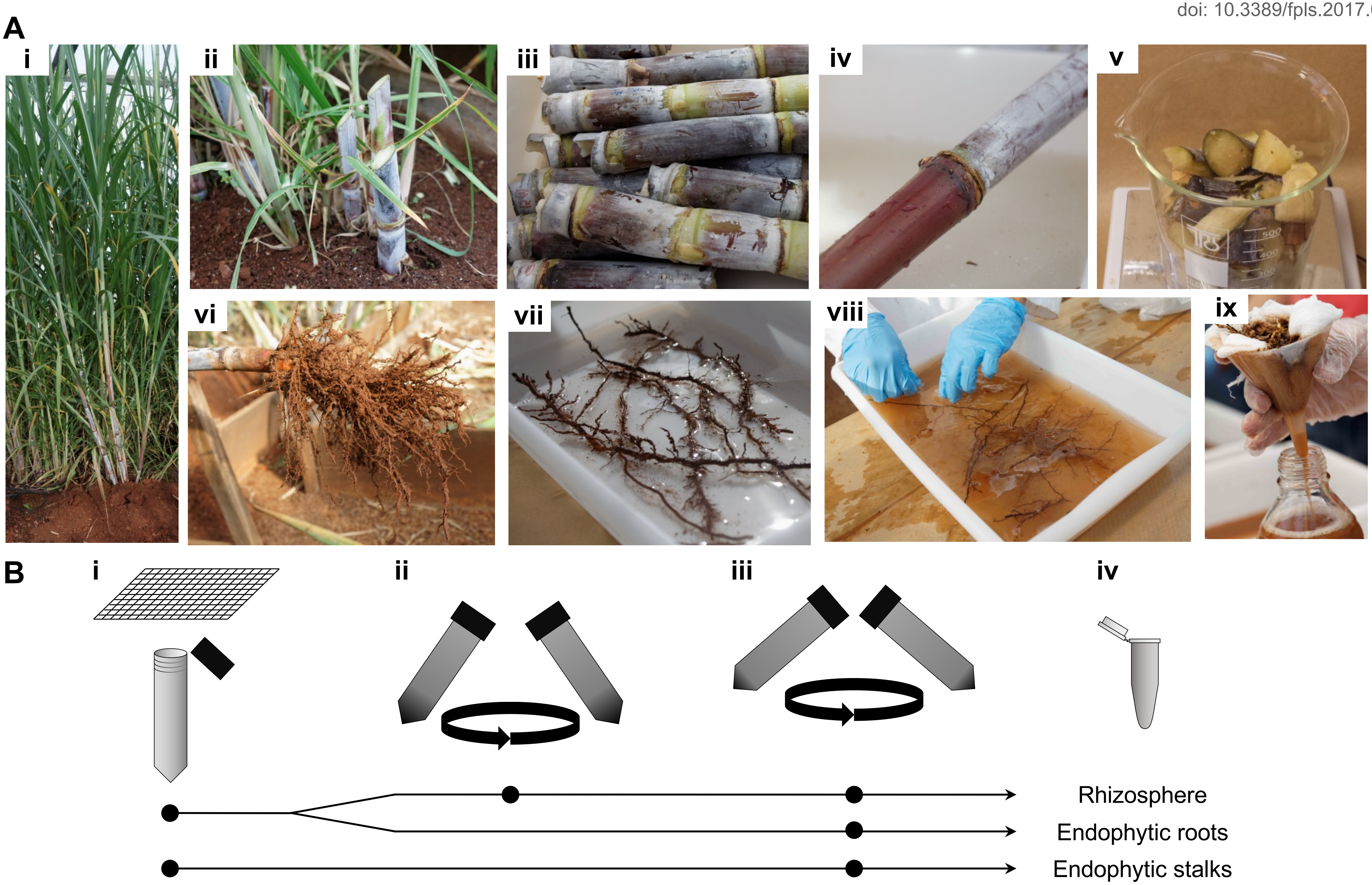

SUPPLEMENTARY FIGURE S1 | Procedure for microbiota isolation from sugarcane organs. **(A)** Stalks and roots were harvested from sugarcane plants **(i)**. Stalks were cut right above root-stalk interface **(ii)** and cut into pieces **(iii)**. Surface-sterilized stalks **(iv)** were cut into smaller pieces **(v)** and blended. Roots were harvested **(vi)**, manually washed to detach rhizosphere particles **(vii,viii)** and filtered through gauze **(ix)**. **(B)** Blended stalks and roots were filtered through gauze **(i)**, centrifuged once or twice **(ii,iii)** and properly stored **(iv)**.
