# Supplementary material for: A Community-Based Culture Collection for Targeting Novel Plant Growth-Promoting Bacteria from the Sugarcane Microbiome
Source: Front Plant Sci. 2018 Jan 4;8:2191. doi: 10.3389/fpls.2017.02191 (PMC5759035; doi:10.3389/fpls.2017.02191)
Supplement: Supplementary file 10 [file Image2.pdf]

doi: 10.3389/fpls.2017.02191

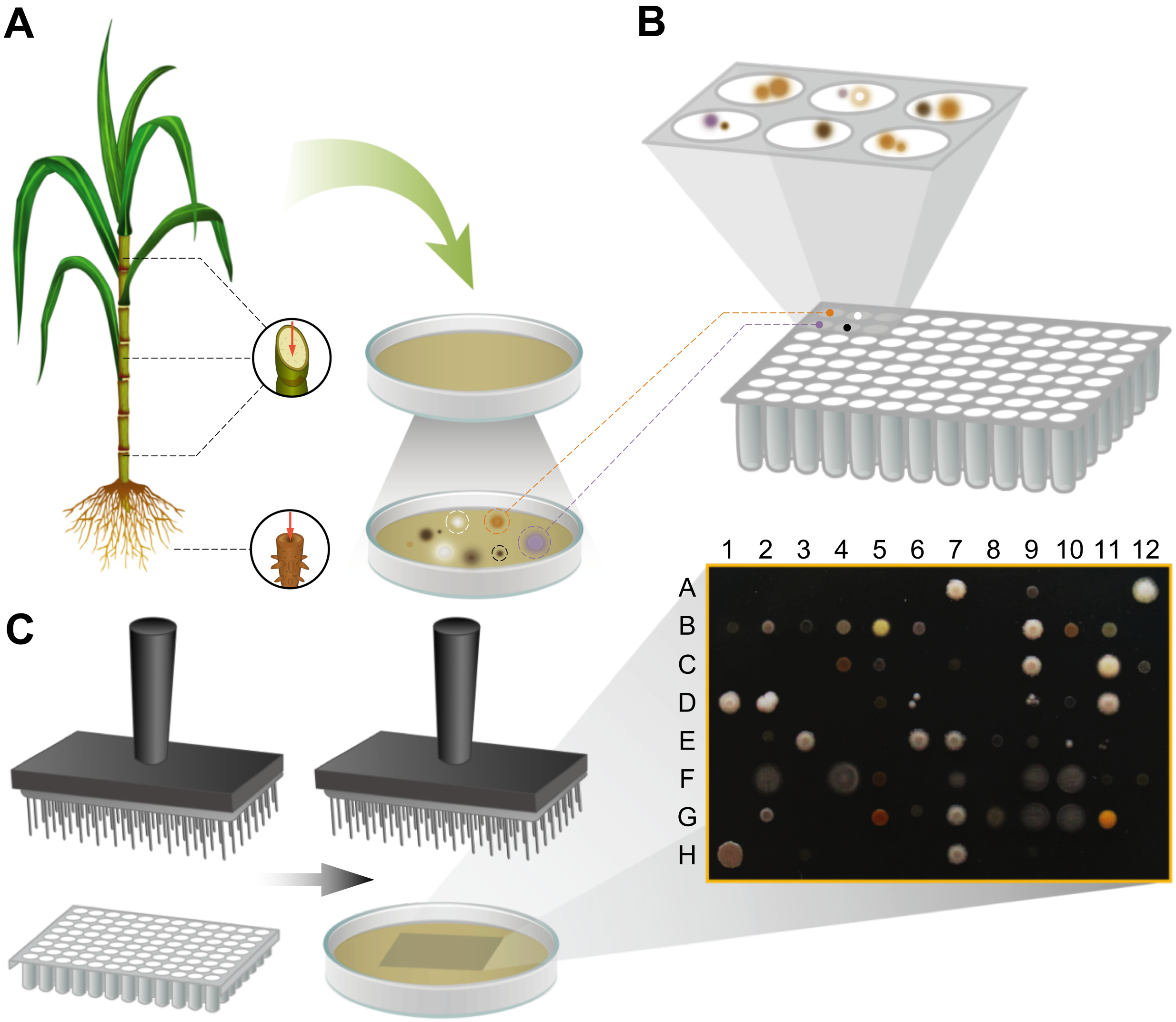

SUPPLEMENTARY FIGURE S2 | A framework of sugarcane CBC construction. **(A)** Enriched microbiota from the rhizosphere and the endophytic roots and stalks were plated on culture media. **(B)** Non-confluent colonies from primary platings were individually picked into 96-well plates with liquid culture media, regardless of whether they were formed by single or multiple microorganisms. **(C)** Growth validation of stored CBC 96-well plates.
