# Supplementary material for: A Community-Based Culture Collection for Targeting Novel Plant Growth-Promoting Bacteria from the Sugarcane Microbiome
Source: Front Plant Sci. 2018 Jan 4;8:2191. doi: 10.3389/fpls.2017.02191 (PMC5759035; doi:10.3389/fpls.2017.02191)
Supplement: Supplementary file 11 [file Image3.pdf]

doi: 10.3389/fpls.2017.02191

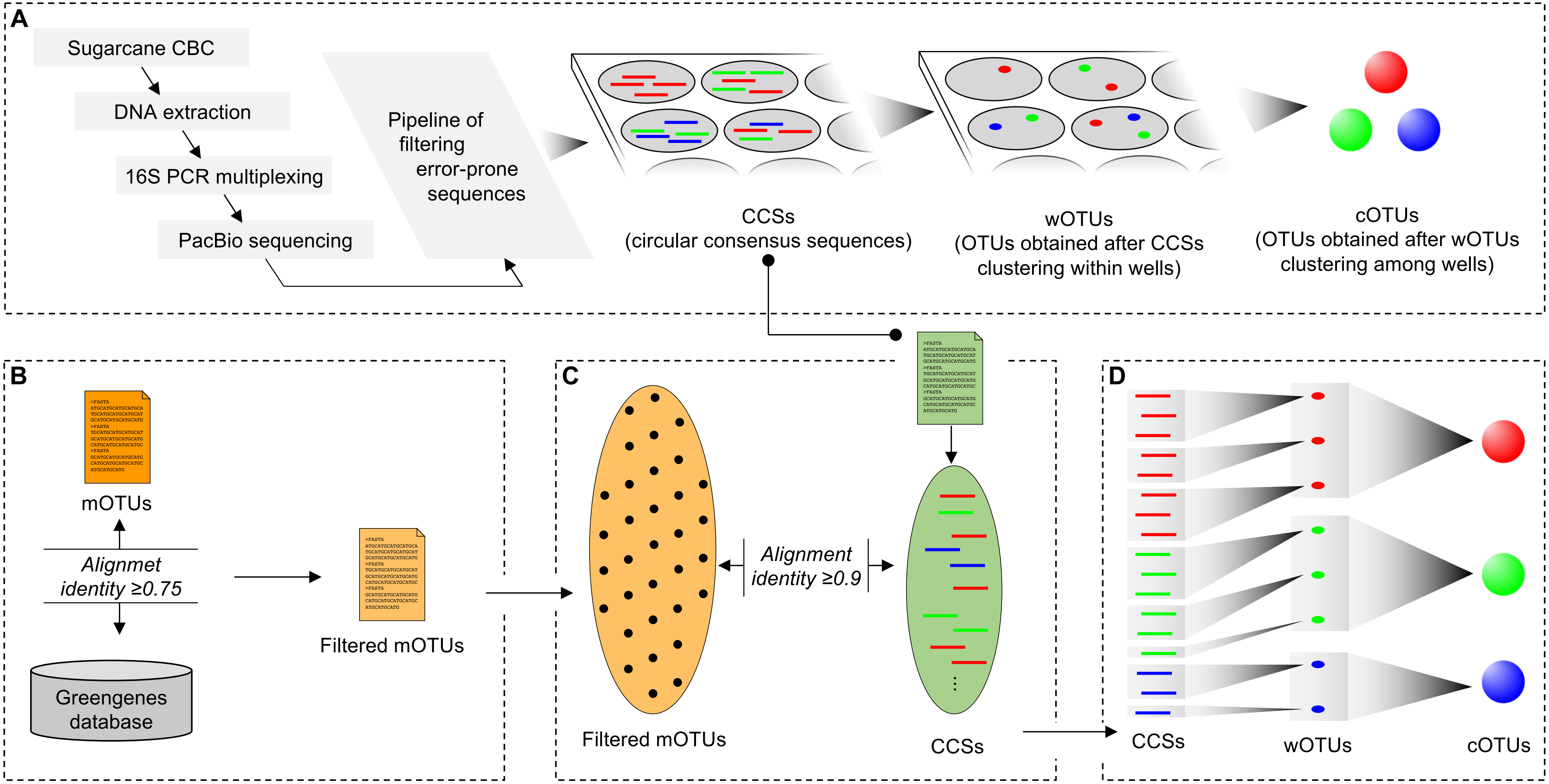

SUPPLEMENTARY FIGURE S3 | Schematic representation of the bioinformatics pipeline for cross-referencing of CBC (culture-dependent) with the sugarcane assembled microbiome (culture-independent) data. **(A)** Annotation of the sugarcane CBC, from stored samples to cOTUs. Demultiplexed raw reads were filtered to ensure reliability and quality of sequences. **(B)** Filtering the dataset of mOTUs in sugarcane organs by alignmnent with the Greengenes. **(C)** Global alignment of filtered mOTUs with CCS dataset of sugarcane CBC using a minimum identity of 90%. **(D)** Mapping back CCSs to cOTUs of sugarcane CBC.
