# Supplementary material for: A Community-Based Culture Collection for Targeting Novel Plant Growth-Promoting Bacteria from the Sugarcane Microbiome
Source: Front Plant Sci. 2018 Jan 4;8:2191. doi: 10.3389/fpls.2017.02191 (PMC5759035; doi:10.3389/fpls.2017.02191)
Supplement: Supplementary file 12 [file Image4.pdf]

doi: 10.3389/fpls.2017.02191

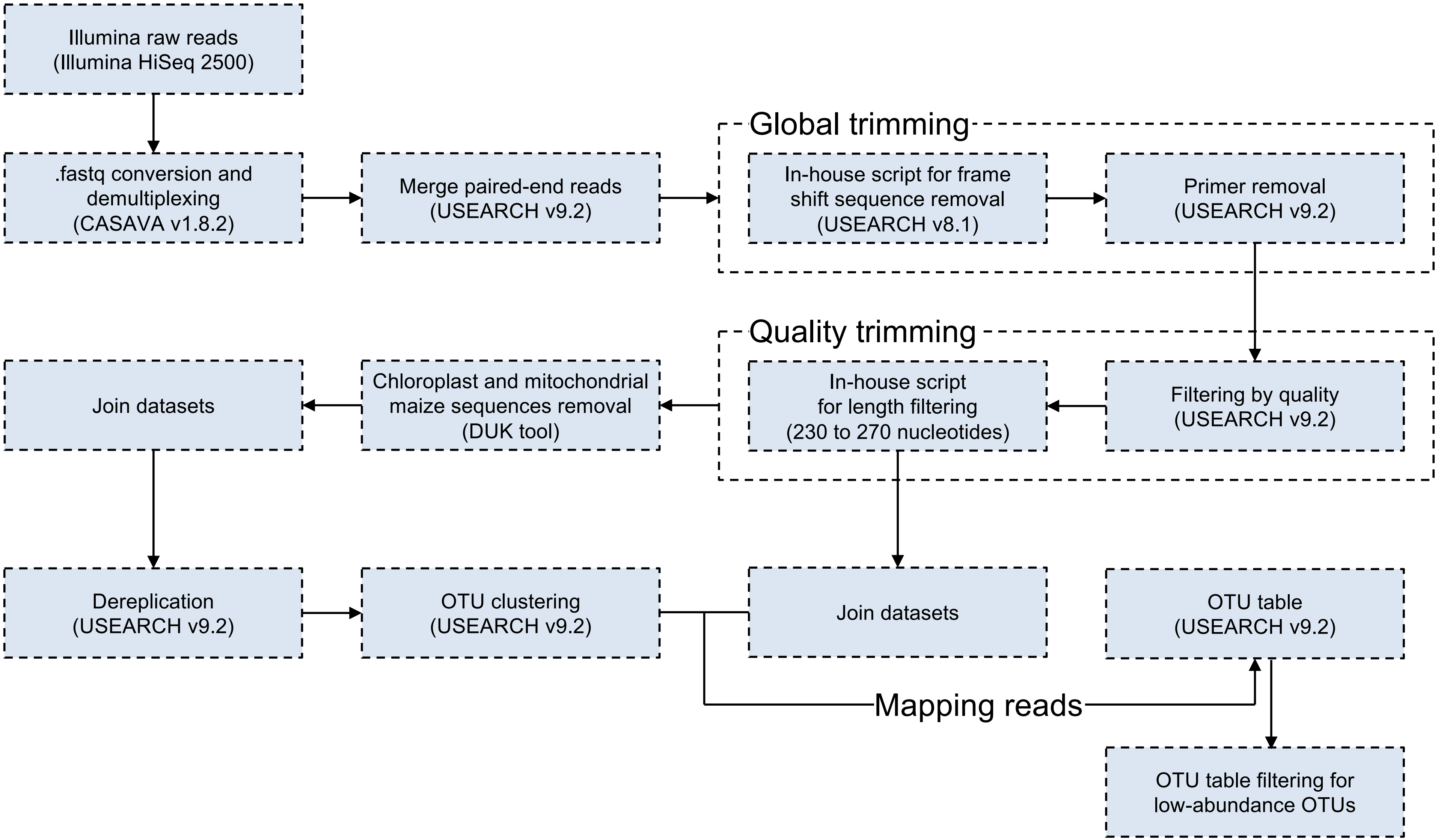

SUPPLEMENTARY FIGURE S4 | Schematic representation of bioinformatics pipeline for Illumina reads data processing. The raw sequences from the HiSeq 2500 were demultiplexed and used in an automated pipeline for read processing and OTU clustering.
