# Supplementary material for: A Community-Based Culture Collection for Targeting Novel Plant Growth-Promoting Bacteria from the Sugarcane Microbiome
Source: Front Plant Sci. 2018 Jan 4;8:2191. doi: 10.3389/fpls.2017.02191 (PMC5759035; doi:10.3389/fpls.2017.02191)
Supplement: Supplementary file 13 [file Image5.pdf]

doi: 10.3389/fpls.2017.02191

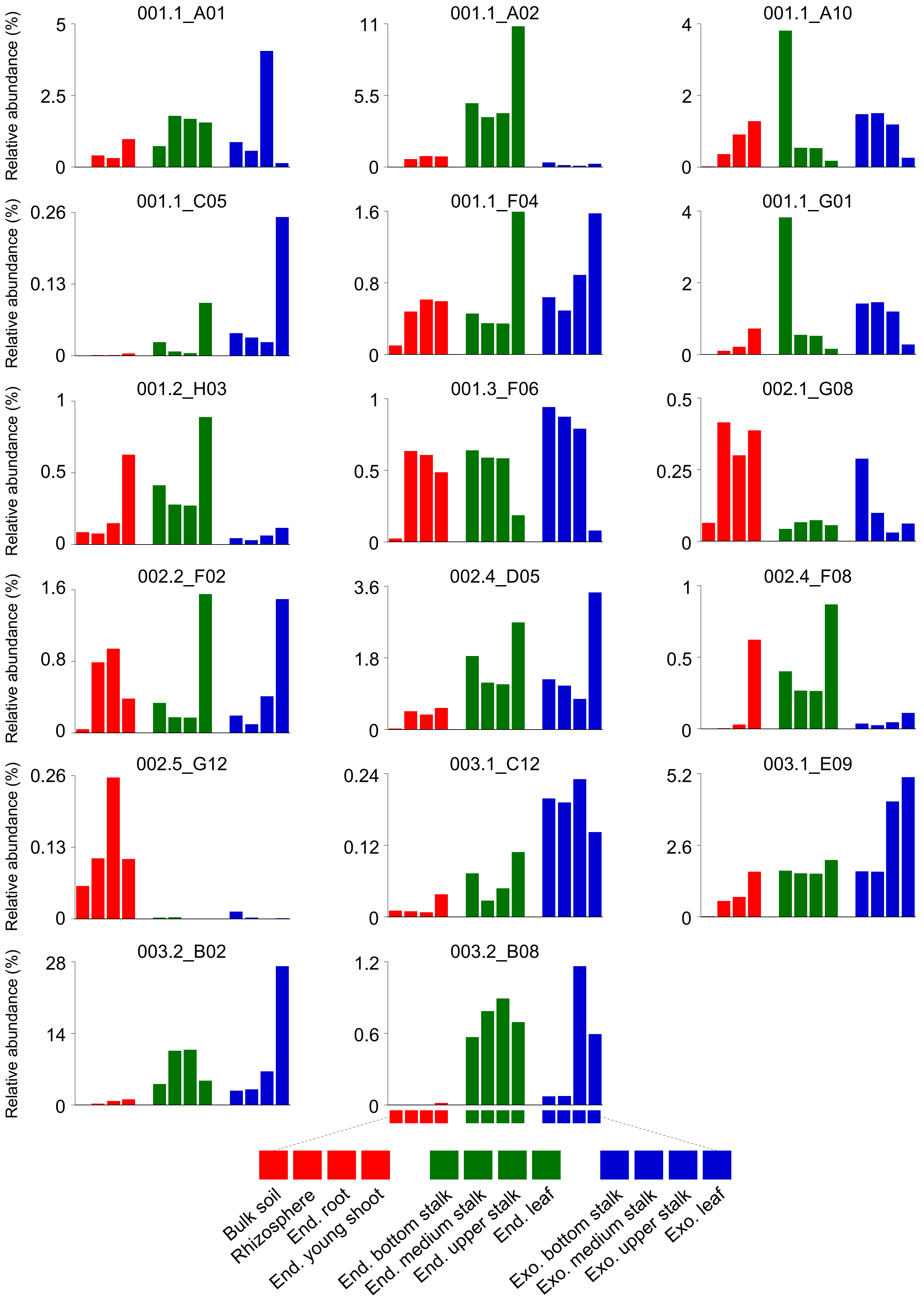

SUPPLEMENTARY FIGURE S5 | Relative abundance in sugarcane organs represented by each well of the the synthetic community. Graphs titles indicate well ID in the CBC. End, endophytic; Exo, exophytic.
