# Supplementary material for: A Community-Based Culture Collection for Targeting Novel Plant Growth-Promoting Bacteria from the Sugarcane Microbiome
Source: Front Plant Sci. 2018 Jan 4;8:2191. doi: 10.3389/fpls.2017.02191 (PMC5759035; doi:10.3389/fpls.2017.02191)
Supplement: Supplementary file 14 [file Image6.pdf]

doi: 10.3389/fpls.2017.02191

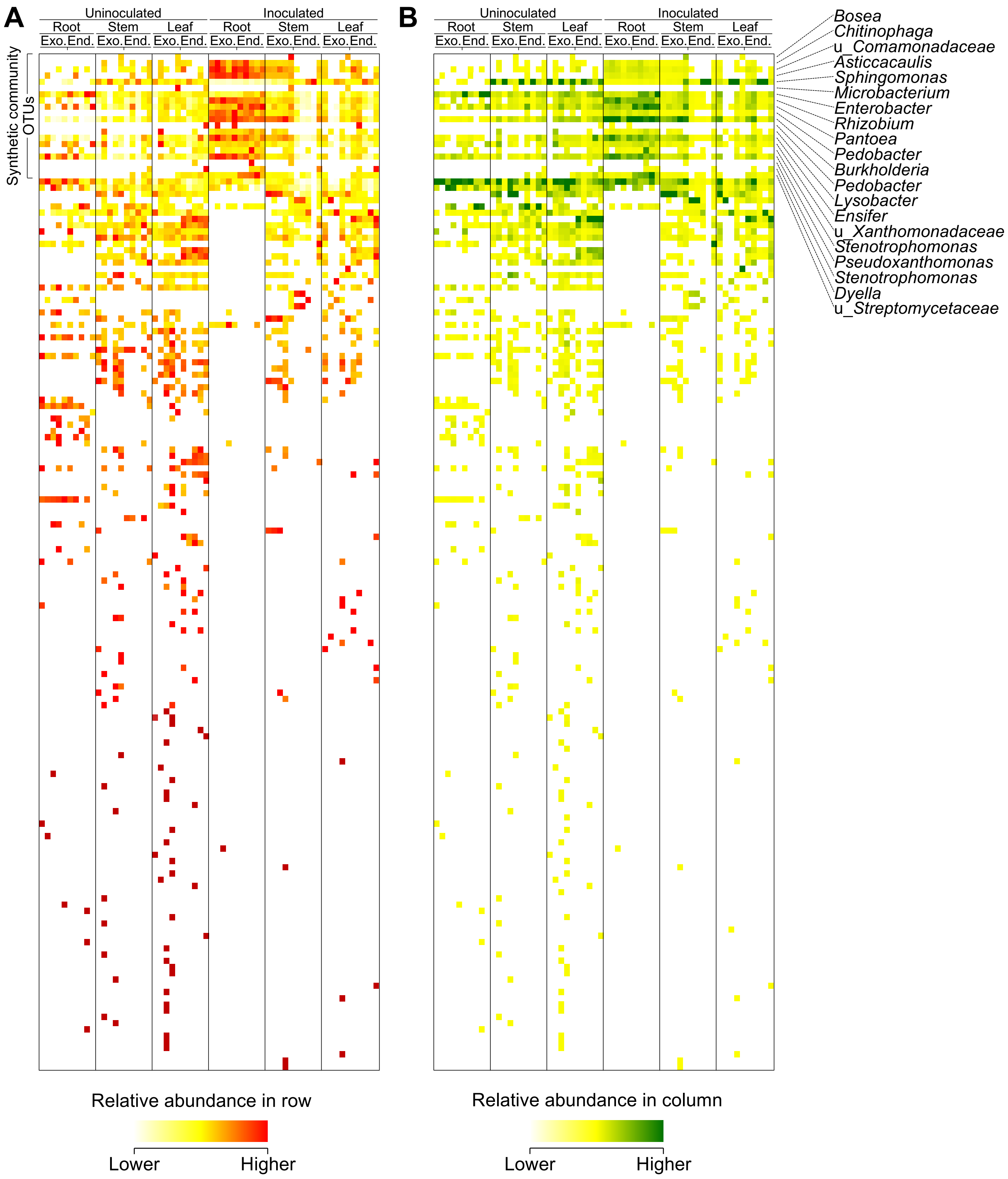

SUPPLEMENTARY FIGURE S6 | Heatmap of the relative abundances of OTUs in organs of the uninoculated and inoculated plants. OTUs considered present in the synthetic community are highlighted, and their taxonomic predictions are shown on the right. **(A)** Color range given by rows. **(B)** Color range given by columns. Exo, exophytic; End, endophytic; u_, unknown.
