# Supplementary material for: A Community-Based Culture Collection for Targeting Novel Plant Growth-Promoting Bacteria from the Sugarcane Microbiome
Source: Front Plant Sci. 2018 Jan 4;8:2191. doi: 10.3389/fpls.2017.02191 (PMC5759035; doi:10.3389/fpls.2017.02191)
Supplement: Supplementary file 15 [file Image7.pdf]

doi: 10.3389/fpls.2017.02191

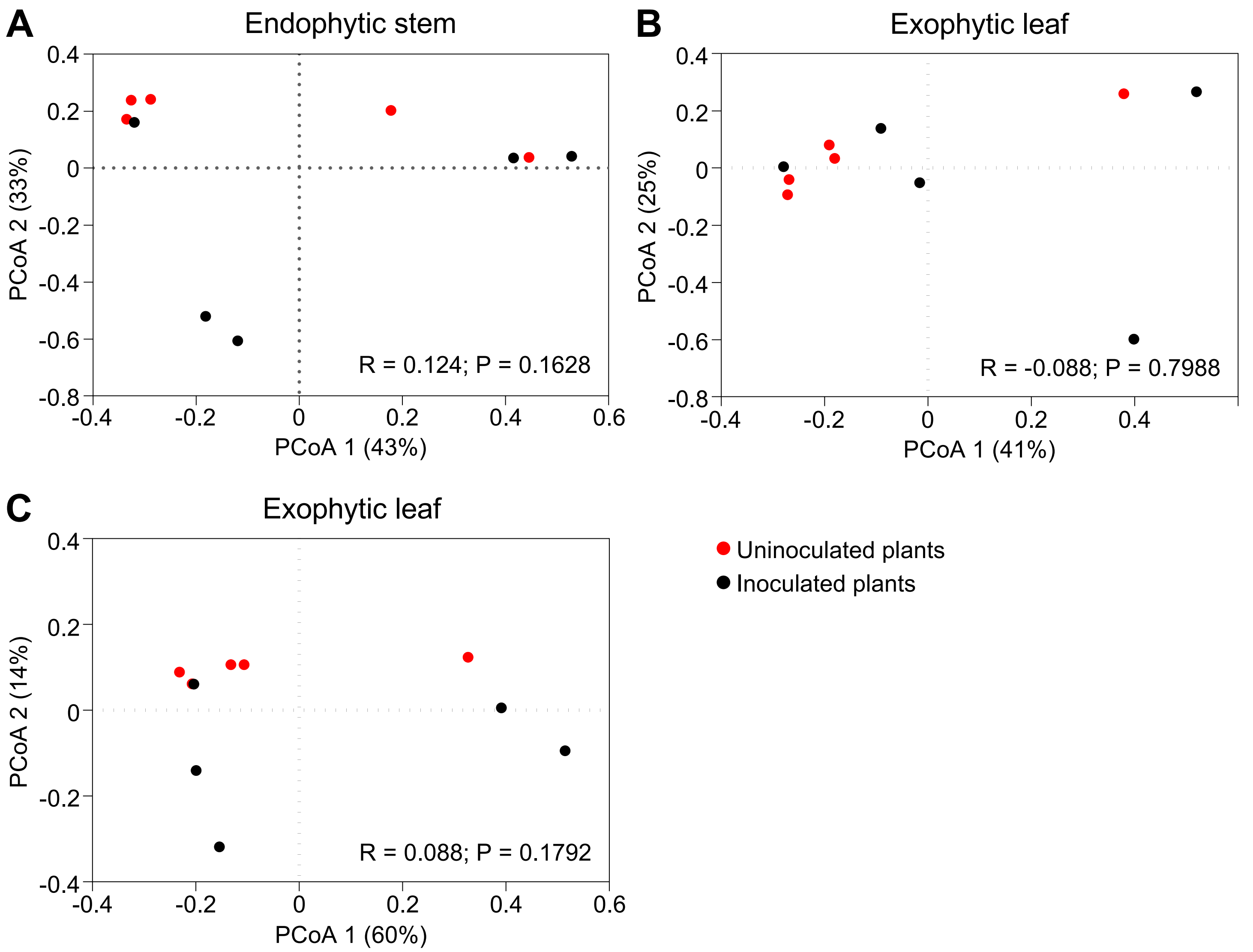

SUPPLEMENTARY FIGURE S7 | Principal coordinates analysis (PCoA) of Bray–Curtis dissimilarity matrix of the stems and leaves of the inoculated and uninoculated plants. Graphs show data of endophytic stem **(A)**, and exophytic and endophytic leaves (**B** and **C**, respectively) of the inoculated and uninoculated plants.
