# Supplementary material for: A Community-Based Culture Collection for Targeting Novel Plant Growth-Promoting Bacteria from the Sugarcane Microbiome
Source: Front Plant Sci. 2018 Jan 4;8:2191. doi: 10.3389/fpls.2017.02191 (PMC5759035; doi:10.3389/fpls.2017.02191)
Supplement: Supplementary file 16 [file SupplementaryMethodsS1.pdf]

A community-based culture collection for targeting novel plant growth-promoting bacteria from the sugarcane microbiome

Jaderson Silveira Leite Armanhi[1,2], Rafael Soares Correa de Souza[1,2], Natália de Brito Damasceno[1,2], Laura M. de Araújo[1,2], Juan Imperial[3,4] and Paulo Arruda[1,2*]

[1]Center for Molecular Biology and Genetic Engineering (CBMEG), University of Campinas (UNICAMP), Campinas, Brazil.
[2]Department of Genetics, Evolution and Bioagents, Institute of Biology, University of Campinas (UNICAMP), Campinas, Brazil.
[3]Centro de Biotecnología y Genómica de Plantas, Universidad Politécnica de Madrid (UPM), Instituto Nacional de Investigación y Tecnología Agraria y Alimentaria (INIA), Campus Montegancedo UPM, Madrid, Spain.
[4]Consejo Superior de Investigaciones Científicas, Madrid, Spain.

*** Correspondence:**

Paulo Arruda
parruda@unicamp.br

Supplementary Methods S1

Construction of the sugarcane CBC

Plant material

Stalks of field-grown, mature sugarcane plants (*Saccharum* sp.) variety SP 80-3280 were planted in the ground soil in the greenhouse of the Center for Molecular Biology and Genetic Engineering at the State University of Campinas, Brazil (22° 49' 8.558" S 47° 3' 32.497" W). Sugarcane plants were grown without fertilization for three harvesting cycles. At the fourth grown cycle, 5-month-old plants were harvested, and the microbial samples were prepared.

Sugarcane juice

The juice was provided by Amyris Biotechnology (Campinas, Brazil), obtained by pasteurization at proper conditions to preserve its chemical and physical integrity. Sugar composition was also provided, obtained by HPLC analysis. Juice total reducing sugars (TRS) were calculated by taking together glucose and fructose concentrations to the sucrose content, using a stoichiometric conversion factor for conversion of sucrose to reducing sugars, as follows: 1 × glucose (g l^{-1}) + 1 × fructose (g l^{-1}) + 1.0526 × sucrose (g l^{-1}).

Culture media

Half-strength Luria-Bertani (LB) medium contained 2.5 g of yeast extract, 5 g of tryptone and 5 g of NaCl per liter. Yeast-peptone-dextrose (YPD) medium contained 10 g of yeast extract, 20 g of peptone and 20 g of glucose per liter. 800 mL of distilled deionized water was added and pH adjusted with NaOH to 7 and 6.5 for half-strength LB and YPD media, respectively. Since half-strength LB medium was supplemented with sugarcane juice at specific concentrations, the broth was filled to the proper volume with distilled deionized water by lacking the volume of juice which was added to the autoclaved medium. YPD medium was filled with water to 1 liter. Solid media contained 15 g of agar per liter. Media were sterilized by autoclaving for 20 min at 1 atm and 120°C on liquid cycle. Autoclaved media were cooled down for 15 min at room temperature when sterile sugarcane juice was aseptically added whenever needed. Half-strength LB media were supplemented with 8 or 35 g per liter of TRS from sugarcane juice. Solid media were routinely poured into Petri dishes (150×20 mm; Sarstedt, Nümbrecht, Germany), solidified at room temperature and stored at 4°C prior to use. Liquid media were stored at room temperature.

Plating and colony picking

Dilution factors were previously determined by serial dilution for each targeted sugarcane organs in order to allow growth of an expected number of colonies on solid media. Enriched microbiotas were then plated at the dilutions of 1/32000 and 1/8000 (for rhizosphere), 1/8000 and 1/2000 (for endophytic roots), and 1/2000 and 1/500 (for endophytic stalk). Microbial samples were plated on 15-cm-diameter Petri dishes containing solidified culture media. Around eight plates were obtained from each sample, distributed to both dilution factors. Samples were plated with sterile glass beads (5 mm diameter; Sarstedt, Nümbrecht, Germany) and incubated for 3 to 14 d at specific temperatures (25 and 30°C, 30 and 37°C, and 30 and 37°C for half-strength LB supplemented with 8 and 35 g l^{-1} TRS, and YPD, respectively). All non-confluent colonies were

picked using sterile toothpicks. Each picked colony was transferred to a single well of a 96-well deep well plate containing 300 mL of liquid medium (same constitutions of solid media used). H10, H11 and H12 wells were used as processes control and lacked colony picking. Plates were shaken at 225 rpm at the same temperatures of solid media used. A total of 350 µL of culture media were added to the wells after 24 and 48 h of picking, and plates were aseptically resealed. After 3 d of shaking, wells contents were homogenized with the aid of micropipette and stored in 96-well plates for DNA extraction (200 µL) and microtiter plates for long-term storage in 27% glycerol at –80°C.

Sequencing and data processing

Library preparation

Briefly, sequencing amplicon libraries were prepared by a two-step PCR. The first step, for 16S rRNA gene amplification and plate barcoding, was performed for all 56 96-well plates of the sugarcane CBC using primers with unique barcodes per plate. The first-step PCR amplicons were pooled in 4- to 6-plate groups and purified using Agencourt AMPure XP Beads (Beckman Coulter, Brea, CA, USA) according to the manufacturer's instruction at a bead-to-DNA ratio of 0.6:1. The purified pooled amplicons were used as template for the second-step PCR, that barcoded row and column of pooled plates. The second-step PCR products were purified as above, validated in 1% agarose, quantified using Qubit dsDNA BR Assay Kit (Invitrogen, Carlsbad, CA, USA) and pooled at equimolar ratios. Each pool of plates led to one single tube and sequenced separately in 11 SMRTcells. Libraries were sequenced using P6-C4 chemistry of PacBio RS II at University of North Carolina (UNC) High-Throughput Sequencing Facility (HTSF, Chapel Hill, North Carolina, USA).

Data processing

A maximum of 3, 1 and 1 mismatches were accepted for plate, rows and columns barcodes in demultiplexing, respectively, evaluated by global alignment. To minimize discard of low-quality sequences, we applied a filter of reliability which retains sequences with at least one hit with the Greengenes 16S rRNA gene database (May 2013 release) and/or the CCS (circular consensus sequence) dataset. CCSs were firstly clustered into OTUs (operational taxonomic units) called well-OTUs (wOTUs, i.e., OTUs obtained after CCSs clustering within wells) which were reclustered into collection-OTUs (cOTUs, i.e., OTUs obtained after wOTU clustering among wells). cOTUs without minimum expected size (1,000 nucleotides) were discarded for OTU-counting but considered in abundances assessment. In the taxonomic assignment, taxon names were kept the same as described in the databases.

Cross-referencing

The microbiome-OTUs (mOTUs, i.e., OTUs obtained by community assemblage analysis of the sugarcane microbiome) were filtered to remove putative *Viridiplantae* mOTUs. Filtered mOTUs were aligned with the CBC CCSs using the command "usearch_global" in USEARCH and the minimum threshold of 90% identity. Two additional subsets of alignments were manually obtained considering 95 or 97% minimum identity.

Maize growth conditions

Endosperm-free seedlings were kept under moist prior to transferring to the greenhouse. After planting, pots were randomly placed on the greenhouse bench. Three germinated embryos were planted per pot, and after 7 d only the best-developed plant was kept per pot. We used 3 × modified Hoagland's solution and sterile distilled deionized water as a control. Hoagland's nutrient solution was composed by 15 mM KNO_3, 15 mM $Ca(NO_3)_2 \cdot 4H_2O$, 6 mM $MgSO_4 \cdot 7H_2O$, 3 mM NH_4NO_3, 3 mM KH_2PO_4 (pH 6.0), 3 mL of micronutrient solution (138.8 µM H_3BO_3, 27.4 µM $MnCl_2 \cdot 4H_2O$, 2.3 µM $ZnSO_4 \cdot 7H_2O$, 0.6 µM $CuSO_4 \cdot 5H_2O$, 1.5 µM $Na_2MoO_4 \cdot 2H_2O$) and 9 mL of 1 g l^{-1} Fe-EDTA.

Microbiota profiling

First, the plants had their leaves cut with a sterile scalpel blade. The remaining leaf roll was then considered as stem and cut from the root system by their root-stem interface. Roots were then carefully removed from the substrate and the excess of vermiculite was manually detached from the root system. The microbiota enrichment was proceeded based on the methodology already described. For exophytic samples, tissues were washed in 100 mL of ice-cold sterile 1 × phosphate-buffered saline (PBS) containing 0.05% Tween 20. The 1 × phosphate-buffered saline was composed by 137 mM NaCl, 2.7 mM KCl, 10 mM Na_2HPO_4, 2 mM KH_2PO_4, and pH 7.0. The leaves, stem and roots were then washed twice in distilled water to remove residual external microbiota. The tissues were then blended in 100 mL of ice-cold sterile 1 × PBS. Plant debris was removed by filtering exophytic and endophytic samples through four layers of sterile bandage. Processed rhizosphere was centrifuged at 200 × *g* for 10 min at 4°C to remove soil debris. Processed endophytic roots, exophytic and endophytic stems and leaves, and supernatant of processed rhizosphere were then centrifuged at 5,000 × *g* for 15 min at 4°C. Supernatant was discarded; the pellet was resuspended in 20 mL of ice-cold sterile 1× PBS and centrifuged at 5,000 × *g* for 15 min at 4°C. The supernatant was again discarded and the pellet was then frozen in liquid nitrogen and stored at –80°C prior to be used. Each of ten plants was processed individually.

Colonization analysis

Library preparation, amplicon sequencing, reads demultiplexing and processing

DNA of a microbial mock community (HM-783D) from BEI Resources (Manassas, VA, USA) was used as a control in library preparation and sequencing. Additionally, DNAs of the 17 wells in the synthetic community were individually extracted from aliquots of grown cultures and used for preparing 16S rRNA gene sequencing libraries. Libraries were also prepared from extracted DNA of the synthetic community (mix of 17 wells prior inoculation) to serve as a control of microbial composition right before the inoculation in plants. All libraries were sequenced twice by independent runs in the HiSeq 2500 Illumina sequencer to establish technical reproducibility thresholds as previously described. Sequencing was performed at the Life Sciences Core Facility (LaCTAD) of the University of Campinas (UNICAMP, Campinas, Brazil). The demultiplexing of reads was performed using CASAVA v1.8.2 (Illumina, San Diego, CA, USA) with 1 mismatch allowed for barcodes. All steps in the bioinformatics pipeline used commands from USEARCH v9.2 unless otherwise specified. Paired-end reads were then merged into extended

reads using “fastq_mergepairs” command parameters “-fastq_maxdiffs 10 -fastq_maxdiffpct 10”. The global trimming step comprised the frame-shift removal and used an in-house developed Perl script. The script uses the “search_oligodb” command from USEARCH v8.1 to find the primers positions without accepted mismatches. Primers were then removed using “fastx_truncate” command and the respective number of nucleotides for each primer as a truncation parameter. The quality trimming step used the “fastq_filter” command and the parameter “-fastq_maxee 0.25”. Then reads were filtered by size from 230 to 270 nucleotides. Maize chloroplast and mitochondrial 16S sequences were removed using DUK tool with parameters “-k 20 -c 2”. The maize chloroplast and mitochondrial sequences were retrieved from NCBI under the accession numbers KF241981.1 (the region from nucleotide 95,184 to 96,675) and DQ490951.2 (the region from nucleotide 43,856 to 45,823), respectively. All files containing data from inoculated and uninoculated plants, and from the wells selected for the inoculum preparation were put together. The UPARSE pipeline was performed, using the “fastx_uniques” command to obtain unique sequences followed by “cluster_otus” for OTU clustering, given by “-minsize 2 -otu_radius_pct 3”. Reads were mapped using “usearch_global” command parameters “-strand plus -id 0.97”.

OTU table construction and analysis

The OTU table was filtered to remove OTUs with relative abundance lower than 0.3%. The threshold for the relative abundance filter was determined by analyzing sequencing technical replicates and the microbial mock community (HM-783D) from BEI Resources (Manassas, VA, USA) using the UPARSE-REF algorithm in USEARCH. From 1,858 OTUs obtained by OTU clustering, 163 remained after filtering. For an OTU to be present in each inoculum selected well a minimum threshold of 1% relative abundance was considered. The OTU table was converted to .biom format and separated using the script “split_otu_table.py” from QIIME. The command “group_significance.py” from QIIME was used to calculate the significance of relative abundance increment of the inoculum OTUs between inoculated and uninoculated plants. Only OTUs with a significant (p-value < 0.05) increase in inoculated plants were considered and used to obtain the sum of relative abundance in each organ.

Graphs

All graphs were plotted and statistical analyses performed using GraphPad Prism version 6 for MacOS, GraphPad Software, La Jolla California USA, www.graphpad.com.
